# Multi-view deep learning of highly multiplexed imaging data improves association of cell states with clinical outcomes

**DOI:** 10.1093/bioadv/vbag010

**Published:** 2026-01-14

**Authors:** Shanza Ayub, Jennifer L Gorman, Edward L Y Chen, Hartland W Jackson, Alina Selega, Kieran R Campbell

**Affiliations:** Lunenfeld-Tanenbaum Research Institute, Sinai Health System, Toronto, ON M5G 1X5, Canada; Department of Molecular Genetics, University of Toronto, Toronto, ON M5S 1A8, Canada; Lunenfeld-Tanenbaum Research Institute, Sinai Health System, Toronto, ON M5G 1X5, Canada; Lunenfeld-Tanenbaum Research Institute, Sinai Health System, Toronto, ON M5G 1X5, Canada; Lunenfeld-Tanenbaum Research Institute, Sinai Health System, Toronto, ON M5G 1X5, Canada; Department of Molecular Genetics, University of Toronto, Toronto, ON M5S 1A8, Canada; Lunenfeld-Tanenbaum Research Institute, Sinai Health System, Toronto, ON M5G 1X5, Canada; Vector Institute, Toronto, ON M5G 0C6, Canada; Lunenfeld-Tanenbaum Research Institute, Sinai Health System, Toronto, ON M5G 1X5, Canada; Department of Molecular Genetics, University of Toronto, Toronto, ON M5S 1A8, Canada; Vector Institute, Toronto, ON M5G 0C6, Canada; Department of Statistical Sciences, University of Toronto, Toronto, ON M5G 1X6, Canada; Department of Computer Science, University of Toronto, Toronto, ON M5S 2E4, Canada; Ontario Institute of Cancer Research, Toronto, ON M5G 1M1, Canada

## Abstract

**Motivation:**

Analysis workflows for highly multiplexed imaging technologies typically summarize each cell in terms of its post-segmentation mean expression, but additional cellular information can be quantified including cell morphology, sub-cellular expression patterns, and spatial cellular context, ultimately giving a multi-modal view of each cell. While deep learning models such as variational autoencoders are well-established for other multi-modal single-cell assays, their ability to integrate these multiple views of a cell from highly multiplexed imaging data remains largely unknown.

**Results:**

Here, we explore the abilities of multi-modal variational autoencoders to learn unified latent cellular representations from multiple views of each single-cell quantified from highly multiplexed imaging, including mean expression, morphology, sub-cellular protein co-localization, and spatial cellular context, while conditioning on technical and batch specific effects. We show that the integrated multi-modal latent space is often more associated with patient-specific clinical outcomes compared to a set of existing baselines. In addition, we perform ablation analyses to understand which input views contribute to model performance, and explore the ability of these models to learn cellular representations that align with cellular phenotypes and enable integration across divergent datasets.

**Availability and implementation:**

hmiVAE is implemented as a python package and is available at https://github.com/camlab-bioml/hmiVAE

## 1 Introduction

Highly multiplexed imaging (HMI) technologies can quantify the expression of 10–100 proteins and their modifications within single cells while retaining their spatial locations. These include immunofluorescence based assays such as cyclic immunofluorescence which use optical imaging (Lin *et al.* 2018, Gut *et al.* 2018), and mass cytometry based assays such as Imaging Mass Cytometry (IMC) and Multiplexed Ion Beam Imaging (Giesen *et al.* 2014, Angelo *et al.* 2014). These technologies have revolutionized cancer research by elucidating spatial tumour heterogeneity with significant consequences for patient outcomes (Jackson *et al.* 2020, Ali *et al.* 2020). The standard analytical workflow of HMI data includes cell segmentation that identifies single cells within the image and generates a cell segmentation mask, containing cell IDs and their spatial locations. Most studies subsequently cluster cells based on the mean protein expression and use spatial information contained in these images to detect patterns in tissue architecture (Gut *et al.* 2018). However, cellular states and functions are additionally determined by protein subcellular localization, cellular morphology, and the influence of local spatial architecture within a tissue (Yousefi *et al.* 2021, Way *et al.* 2022, Li *et al.* 2021).

Deep learning methods are gaining popularity as analysis tools for HMI data but have been largely limited to image segmentation and cell type annotation (Zidane *et al.* 2023). Improved segmentation using deep learning in HMI data allows for accurate identification of cells within a tissue image which is crucial for cell type assignment and further downstream analysis has revealed distinct communities in the tumour microenvironment (TME) associated with clinical outcomes (van Maldegem *et al.* 2021, Greenwald *et al.* 2022, Rahim *et al.* 2023). Deep learning methods have also improved and automated identification of major cell types in tissues (Lee *et al.* 2017, Geuenich *et al.* 2021, Amitay *et al.* 2023), but these methods cannot be used to identify variations within these major cell types as they are limited to using single-cell protein expression alone and do not take into account the spatial context of the cells. Some studies incorporate spatial information and the tissue context to understand cell states (Zhang *et al.* 2022, Tanevski *et al.* 2022) but these ignore the impacts of protein subcellular localization on cell state and differences in cell morphology.

Spatial transcriptomics (ST) and single-cell RNA sequencing (scRNA-seq) have seen more application of deep learning methods, largely for batch correction, spot deconvolution, and spatial domain characterization with the aim to identify cell types and states (Lopez *et al.* 2018, 2022, Maseda *et al.* 2021, Min *et al.* 2025). Methods such as STG3Net (Fang *et al.* 2024a), STMGAC (Fang *et al.* 2024b), and SpaMask (Min *et al.* 2025), have coupled masked graph neural networks with either contrastive or adversarial learning to improve domain identification and gene denoising. Further, existing deep learning methods integrate different modalities such as totalVI, which integrates cell surface protein expression with transcriptomics data (Gayoso *et al.* 2021) and DeepST and SpatialDIVA which use data from histology and spatial transcriptomics images to identify spatial domains of clinical importance (Xu *et al.* 2022, Maan *et al.* 2025). Many of these models follow a Variational Autoencoder (VAE) framework and have shown state-of-the-art performance in integrating multi-modal data inputs, enabling the classification of cell states not identifiable by a single data type alone (Flores *et al.* 2022). VAEs are generative deep unsupervised models that learn a latent space—a compressed lower dimensional representation of the high dimensional input data—that tries to strike a balance between accurate data reconstruction and model generalizability (Kingma and Welling 2014, Higgins *et al.* 2016, Simidjievski *et al.* 2019). The lower dimensional latent representation learnt by a VAE captures relevant features in the data that can be used for downstream analysis such as clustering and cell state identification as well as associations with clinical variables using patient data (Lopez *et al.* 2020, Chow *et al.* 2022).

Here, we propose a multi-view VAE for highly multiplexed imaging data (hmiVAE) that learns combined and mode-specific latent representations from input mean expression, morphology, protein nuclear co-localization, and spatial cellular context, while conditioning on technical and batch specific effects. We apply this model to several IMC datasets of breast and melanoma cancers (Jackson *et al.* 2020, Ali *et al.* 2020, Hoch *et al.* 2022) and apply the integrated multi-view latent space to many common downstream tasks including: (i) associative modelling of clinical outcomes and patient survival across different clinical subgroups; (ii) cell type annotations; (iii) robustness to cell segmentation; (iv) batch correction; and (v) latent space projection between two IMC datasets. We quantify the extent to which the integrated multi-view latent space is associated with specific clinical outcomes compared to existing approaches. Next, we show that our expression-specific representation can identify cell clusters more closely aligned with known lymphocyte and epithelial subpopulations compared to established workflows. Finally, we test the ability of our model to perform consistent batch integration across different cohorts. Together, this study provides the first exploration of using multi-modal VAEs to learn meaningful cell representations from highly multiplexed imaging data and demonstrates its utility on several common downstream analysis tasks.

## 2 Methods

### 2.1 Datasets used

The Jackson-BC dataset comprised 358 images obtained from a Tissue Microarray (TMA) of tissue sections sourced from breast cancer patients, encompassing 802 591 cells and incorporating 36 proteins, including two DNA intercalators (Jackson *et al.* 2020). The Ali-BC dataset, derived from the METABRIC study (Curtis *et al.* 2012), featured 548 images from a TMA of tissue sections of patients with primary invasive carcinoma. This dataset included 536 883 cells and utilized 39 stains, including two DNA intercalators (Ali *et al.* 2020). Notably, both datasets shared an antibody panel designed to target epitopes specific to breast cancer, encompassing markers for cell cycle regulation, phosphorylation-based signalling, and distinctive markers for epithelial, endothelial, mesenchymal, and immune cell types. The Hoch-Melanoma dataset comprised 167 images extracted from a TMA of biopsies originating from patients with stage 4 or stage 3 melanoma. This dataset encompassed 989 404 cells and employed 46 stains, incorporating two DNA intercalators. The antibody panel for the Hoch-Melanoma dataset included markers for tumour cells, alongside those targeting various immune cell types and their activation states (Hoch *et al.* 2022). Each dataset was accompanied by comprehensive clinical and survival information, detailed in Table 1, available as supplementary data at *Bioinformatics Advances* online.

### 2.2 Feature derivation

Each dataset consists of a: (i) three-dimensional expression array which contains the pixel-level protein expression counts, (ii) cell segmentation mask array which contains cell ID assignments for each pixel and (iii) channel-to-protein identification dataframe which associates each z-stack in the expression array to a protein measured in the experiment. The mask and expression arrays have the same x and y coordinates, and the size of the z-axis in the expression array corresponds to the number of channels in the channel file.

Expression features: To create the protein mean expression features, we subset to pixels belonging to the same cell ID (as contained in the mask array) in the expression array for each protein. We average over the counts over these pixels for each protein to give the average count for expression within that individual cell. We do this for each cell creating a *N* x *P* matrix where *N* is the number of cells in the dataset and *P* is the number of proteins measured in the experiment. We denote this matrix **Y**.

Protein nuclear localization score features: Due to the resolution of IMC, we restricted protein subcellular localization to in-nucleus or not in-nucleus. Therefore, this should be treated as a protein nuclear localization score. We generate this score by first creating a ‘mean nuclear stain’ by taking the average of the two Iridium DNA stains for each pixel. We subset to pixels belonging to the same cell ID and then do a pixel-wise correlation between each protein and this mean nuclear stain. A high correlation means that the same pixels within a cell have high expression of a given protein and a high mean nuclear stain, suggesting that this protein is expressed in the nucleus. We do this for each cell creating a *N* x *P* matrix where *P* is the number of correlations (note here that the number of correlations equals the number of proteins measured in the experiment). We call this matrix **S**.

Morphology features: We use Python’s regionprops function from the scikit-image library (van der Walt *et al.* 2014) to create the morphology features: area, perimeter, and eccentricity. We also adopt the concavity and asymmetry features from DeepCell (Greenwald *et al.* 2022). We compute these features for each cell, thereby creating a *N* x *M* matrix where M is the number of morphology features. In our case, *M *= 5. We call this matrix **M**.

Spatial context features: For the spatial context features, we first compute the 10 nearest neighbours based on spatial coordinates for each cell using scanpy (Wolf *et al.* 2018, Cover and Hart 1967 ). Using these neighbours, we create a *N* x *N* sparse matrix **D** s.t. **D**_*ij*_ = 1 if cell *i* is the neighbour of cell *j* and 0 otherwise. We then scale **Y**, **S**, and **M** to be such that each feature in these matrices has a mean 0 and variance 1 and concatenate these together. The sparse matrix **D** is then multiplied with this concatenated matrix, with each component normalized by the number of each cell’s neighbours. This gives us a *N* x *L* matrix, where *L *=* P *+* C* + *M*. We call each row the spatial context of a cell, and denote this matrix by **C**.

Background features and one-hot encoding: We create a sample-wise one-hot encoding for each cell i.e. for a given cell, the vector is of length *R* for *R* number of samples and has a 1 in position *r* if the cell belongs to sample *r* and 0 otherwise. Along with the one-hot encoding, we create several background covariates. (i) When background stains such as ArAr80 were available, we computed the mean background channel staining per cell and correlated this mean background stain with the mean protein expression per cell for all proteins. A high correlation between the background stain and a protein’s expression across cells would suggest a non-specific staining for that protein. (ii) Since staining efficiency can vary between tissue samples (Milosevic 2023), we also computed the ‘mean sample intensity’ which we define as the average pixel intensity over all channels for an image. (iii) We also computed the average value of signal intensity across all the background channels per sample.

In the case where information about background stains was not available, we computed the sample intensity and the average background stain of each protein. These background stains were defined as the mean over a protein’s expression values for 0-labelled pixels (in the mask file, pixels belonging to a cell are denoted by a non-zero integer corresponding to that cell’s ID, whereas pixels that are labelled as ‘0’ are treated as background). We denote all background features and one-hot encoding covariates by *b*.

### 2.3 hmiVAE architecture

hmiVAE follows a Variational Autoencoder architecture (Kingma and Welling 2014). It consists of two neural networks: an encoder and a decoder. The encoder takes in a data matrix corresponding to each view, along with the additional covariates outlined in *Background features and one-hot encoding* and generates a separate embedding for each of the four views: protein mean expression, protein subcellular localization, cell morphology and spatial context. These view-specific embeddings are then concatenated, passed through an additional hidden layer and integrated to create a latent representation *z* for each cell informed by all four views. The decoder takes in the latent representation of each cell and any additional covariates input to the encoder, i.e. the input to the decoder is concatenated as *z + b*. The non-linear decoder follows a symmetric architecture to the encoder in which integrated hidden layers are followed by view-specific hidden layers that reconstruct each view separately. All layers are made using modules available in Pytorch (Paszke *et al.* 2019).

### 2.4 Model training

Hyperparameter tuning: We tuned the model by varying 6 hyperparameters: (i) initialization (random seed—0, 1, 42, 123, 1234), (ii) number of hidden layers (1 or 2), (iii) hidden layer size (i.e. number of nodes in each hidden layer—8, 32, 64), (iv) latent representation dimension (10 or 20), (v) beta scheme [i.e. warmup of the weight of the KL-term in the Evidence Lower Bound (ELBO) loss, starting at zero and incrementing by 0.1 after each epoch, or keeping the weight of the KL-term constant at 1.0], and (vi) batch size (i.e. 40 000, 16 000, 8000, 5333, 4000). We optimized the ELBO loss function to train our method as defined below:


$$\mathrm{ELBO}\;=\sum_{j\in \{E,NC,M,SC\}}\sum_{i=1}^{N}\lambda_{j}E_{q}[\log\;p(x_{i,j}|z)]-\beta \;\mathrm{KL}(q(z|X)||p(z))$$

In the above equation, the left-hand side of the expression is the reconstruction likelihood for all views summed, the right-hand side is the KL-divergence between the estimated distribution, *q*, and the true distribution, *p*. The feature vector for each view *j* and cell *i* is represented by *x_i_*_,_  _*j*_, *z* is the latent space feature vector, and *X* is the set of all feature vectors or data i.e. $X=\{x_{E},\;x_{NC},\;x_{M},\;x_{SC}\}$. λ_j_ denotes the weight of each view in the full reconstruction likelihood term. For the training with the full model, all weights are set to 1.

We created train and test splits for each dataset and used a grid search algorithm over the different combinations of these hyperparameters and selected the combination that resulted in the best reconstruction likelihood over test data. During all training runs, to guard against overfitting, we include early stopping from pytorch-lightning (Falcon and The PyTorch Lightning team 2019), which stops the model training when the loss on the validation dataset is minimized.

Run with best model: hmiVAE was run on each dataset with the combination of hyperparameters that resulted in the best test likelihood for that dataset. The optimal combinations for hyperparameters for each dataset are detailed in Table 2, available as supplementary data at *Bioinformatics Advances* online. The whole dataset is passed through the trained model to generate the latent representations as well as the view-specific embeddings for each cell in the dataset.

Clustering over view-specific embeddings: For creating clusters, first we computed distances between all cells in the integrated and view-specific latent spaces and found the 100 nearest neighbours within the corresponding latent space for each cell using the neighbours function from scanpy (Wolf *et al.* 2018). Next, we use the Leiden algorithm with resolutions 1.0, 0.5, 1.0, 0.1, and 0.5 for clustering over the latent space, expression, correlation, morphology and spatial context embeddings respectively. For the Ali-BC dataset, we clustered over the expression-only cell embedding using a Leiden resolution of 1.0.

Clustering over original and latent features with FlowSOM and Leiden: We used the Leiden algorithm (Traag *et al.* 2019), a popular community detection algorithm used in single-cell data analysis, and FlowSOM (Van Gassen *et al.* 2015, Quintelier *et al.* 2021, Couckuyt *et al.* 2024) to cluster cells using the original and latent features. To mimic the standard workflow in IMC cluster analysis, we use three choices for the number of nearest neighbours i.e. 10, 50 and 100, and find the neighbours for each cell over the original and latent feature spaces. We then tried different values for the resolution parameter in Leiden i.e. 1.0, 0.5, and 0.1 and store the resulting cluster assignments to an AnnData object.

FlowSOM, on the other hand, uses self-organizing maps to sort single cells into clusters (Van Gassen *et al.* 2015) and requires users to set the number of expected clusters. It does so iteratively until the cell membership of the clusters stop changing or max iterations are reached. For the SOM grid, we use three different sizes: 6 × 6, 10 × 10 and 20 × 20. We use two settings for the number of neurons for clustering over the original and latent feature set: (i) the number of clusters that were found using Leiden clustering with 100 nearest neighbours and a resolution of 1.0 and (ii) the number of clusters that were found using Leiden clustering with 10 nearest neighbours and resolution of 0.1 over the latent space.

### 2.5 Ranking of feature drivers per cluster and cell type annotation

Ranking features for integrated clusters: To identify the features driving the clustering in the integrated latent space of hmiVAE, we applied the rank_gene_groups function from scanpy (Wolf *et al.* 2018) using the features from each view separately. For each cluster, we selected the top three features which had a positive t-value and an adjusted *P* value of less than .05. We combined all the features across all clusters and as this list would contain lots of repeated features, we subset the list to only unique features across the clusters and then selected their values for each cluster. For the final list of feature drivers across all clusters, we relaxed our criteria for statistical significance and t-value to include the values for those features which were not significantly different for some clusters among the list of unique features.

Annotation of cell types: For our cell type annotation we used the rank_gene_groups function from scanpy (Wolf *et al.* 2018) and used violin plots for visual confirmation after applying a StandardScaler scaling of the expression features. For the Ali-BC and Jackson-BC datasets, within the epithelial cell lineage, we used CK8/18, CK19, CK7, and panCK as markers for luminal epithelial cells and CK5 and CK14 for basal epithelial cells. CD31/vWF was used as a marker for endothelial cells, and Fibronectin was used to determine fibroblasts and general stromal cells along with Vimentin and SMA. For immune cell lineage, we used CD45 and CD20 for B cells, CD45 and CD3 for T cells and CD45 and CD68 for macrophages. Some cell state markers were also included e.g. Ki67 for proliferation and CAIX for hypoxia.

For the Hoch-Melanoma dataset, the panel consisted of tumour cell markers (e.g. MiTF, SOX10, -Catenin) and immune cell markers (e.g. CD3, CD8, CD303, etc.) and so, the cell types correspond largely to these lineages. The panel also consisted of markers for T-cell activation such as CD45RO for memory T cells and CD45RA for naive T cells. Further details of which markers were used in assigning each cell type label are described in Table 3, available as supplementary data at *Bioinformatics Advances* online.

Any clusters that did not show any significant expression for the lineage markers as described above were annotated as ‘unknown’ or ‘none’. Clusters showing significant expression of belonging to different lineages or in cases where we could not assign a single cell type label were annotated as ‘mix’. We carried out the same workflow to annotate cell types identified by FlowSOM and Leiden by clustering over the expression-only features.

### 2.6 Benchmarking cell type annotations

Comparisons using published dataset annotations: For the Ali-BC and Jackson-BC datasets, the single-cell assignments were retrieved from the relevant publications. For the Ali-BC dataset, we converted our annotations from running Leiden on hmiVAE’s expression-only embedding features, and annotations from running Leiden and FlowSOM on the original expression features, into pandas dataframes, and merged it with the dataframe containing the Ali-BC publication labels using the sample ID and cell ID. Similarly, for the Jackson-BC dataset, we first matched each single-cell to its published meta cluster ID and created sequential cell IDs for our cell labels to match those from the publication as the labels were not sequential in the original masks which generated the cell IDs, however, it was a simple one-to-one matching to create the sequential labels. The dataframes containing the published cell labels and our annotations from hmiVAE’s expression-only embedding and original features were then merged as described earlier. The labels were compared using the Balanced Adjusted Rand Index function available from balanced-clustering package (Maan *et al.* 2024).

Comparisons using manual ground truth annotation of 500 cells: This was only performed for the Jackson-BC dataset as this was the only dataset that had any manually labelled ground truth annotations. These annotations were made by two independent annotators for a subset of 500 cells, further details on how the annotations were done are described elsewhere (Geuenich *et al.* 2021). Briefly, annotators selected 500 random cell IDs and assigned them cell type labels by visually inspecting them using cell type markers such as those described in Table 3, available as supplementary data at *Bioinformatics Advances* online. We edited the format of our cell IDs and merged the dataframes as described earlier and computed the Balanced Adjusted Rand Index using the balanced-clustering package as before (Maan *et al.* 2024). We also computed the precision, recall, specificity and F1-score for the cell type classifications over these 500 cells using the classification report and confusion matrix functions from the scikit-learn package (Pedregosa *et al.* 2011).

### 2.7 Association with clinical variables

We did so by using two methods to define *cluster prevalence*: (i) We summarized the proportion of cells belonging to a sample from each cluster ID and (ii) the prevalence of cells from each cluster per mm^2^ of patient tissue. We did this analysis for cluster IDs which resulted from clustering over the full original feature set and the latent feature space for FlowSOM and Leiden clustering run with different nearest neighbour and resolution parameters.

To get the proportion of cells belonging to a cluster ID, for each sample ID, we counted the number of cells that belonged to each cluster ID and divided that number by the total number of cells within a sample. This resulted in a number between 0 and 1 for each cluster ID and these numbers all summed up to 1. We call this *cluster proportion*.

To get the prevalence of each cluster as an instance per mm^2^, for each sample ID, we counted the number of cells that belonged to each cluster ID and divided it by the dimensions of the image belonging to the sample ID. To make computation easier, we multiplied the resulting number with 1e6 (assuming an image of size 1000 × 1000 pixels). We call this *cluster instances per mm^2^ of tissue*.

Association with clinical variables: To understand and compare the association between the resulting clusters, we conducted an association analysis between cluster prevalence and clinical variables for all clustering methods using both original and latent features across all three datasets.

Association of latent space with clinical variables: For association between the latent space and clinical variables, for each sample in the dataset, we first calculated the median cell embedding for each dimension of the latent space. Next, we selected patients belonging to the same category for each clinical variable and ran a logistic regression with all the median latent space values to find the associations of each latent dimension with each category for a given clinical variable.

During this analysis, we found that the inclusion of some latent dimensions for a few clinical variables resulted in a singular matrix during computation, so we excluded them and re-ran a logistic regression with the remaining latent dimensions. For some categories of clinical variables, there were not enough samples, so these were removed from the analysis.

Association of cluster proportion with clinical variables: Based on our definition of cluster proportion; in order to prevent a degenerate matrix in our analysis, we ran a logistic regression one cluster ID at a time using a 1 vs. rest approach. We did this for all cluster IDs and all categories present within all clinical variables. Categories that did not have enough samples, we removed or recorded with a ‘NA’ t-value.

Association of cluster prevalence as instances per mm^2^ of tissue: For the association between cluster instances per mm^2^ of tissue, we did not have the same issue of a degenerate matrix as in the case of cluster proportion and found that the scaling by 1e6 aided in this. Therefore, we ran a logistic regression over all the cluster IDs for all categories within every clinical variable. Similarly, as before, categories that did not have enough samples were removed or recorded with a ‘NA’ t-value.

### 2.8 Survival analysis using cox proportional hazards models

To find the relationship between patient survival outcome and cluster prevalence, we used the python lifelines package (Davidson-Pilon 2019) to fit a Cox proportional hazards model including disease stage and grade (where available) as a covariate. We carried out this analysis for each cluster ID at a time, for both cluster proportions and cluster instances per mm^2^ of tissue. *P* values for hazard ratios were adjusted by Benjamini-Hochberg correction.

### 2.9 Ablation study

As described in the Section 2.4, a weight is assigned to each view in the reconstruction likelihood of the full loss function. Therefore, to ablate each view, we set the weight of the ablated view to 0 and set all else to 1, for example if we ablated the correlation view then λ_C_ = 0, while λ_E_, λ_M_, and λ_SC_ are set to 1. For the expression only run, we set all λ’s to 0 and only set λ_E_ to 1. We train ablation models for each dataset using the combination of hyperparameters determined during the hyperparameter tuning step of the full model (Table 2, available as supplementary data at *Bioinformatics Advances* online).

To cluster over the latent features resulting from each ablation run, we used FlowSOM and Leiden clustering with 100 nearest neighbours and resolution parameter set to 1.0. We then summarized cluster prevalence for each ablation run as before (described in the *Association with clinical variables* section) and carry out survival and clinical association analysis as before.

### 2.10 Generation of the PDAC dataset

#### Ethical approval

The COMPASS trial (NCT02750657) was approved by the Research Ethics Boards at the University Health Network (REB # 15-9596) and Mount Sinai Hospital (REB # 20-0170-E).

#### Imaging mass cytometry

Antibody conjugation: All antibody conjugations were performed using the Maxpar X8 Multi-metal Labeling Kit according to the manufacturer’s instructions (Standard BioTools, 201300). In addition to metals supplied with the kit, yttrium-89 (Sigma, 217239-10G) and platinum-194 and -195 (Standard BioTools, 201194 and 201195) were purchased separately. Following conjugation, antibodies were diluted to 500 µg/mL and stored in PBS-based antibody stabilizing buffer (CANDOR Bioscience, 131–125) at 4°C.

Tissue processing and staining: Specimens from the COMPASS trial were collected, processed, and sectioned at the University Health Network and then transferred to the Lunenfeld-Tanenbaum Research Institute for IMC staining and acquisition.

Formalin-fixed, paraffin-embedded 5 µm tissue sections were baked at 60°C for 45 minutes, deparaffinized in xylene, and then rehydrated in a reagent alcohol series as follows: 100% × 2, 96%, 90%, 80%, and 70%, for 5 minutes each. Heat-induced antigen retrieval was performed at 95°C in Tris-EDTA pH 9.2 buffer within a decloaking chamber for 30 minutes (Biocare Medical). Following incubation, buffer was allowed to cool to room temperature before slides were removed. Tissue was encircled in hydrophobic pen and incubated in blocking buffer containing 3% BSA (Sigma, A3059-50G) and 5% horse serum (Sigma, H0146) in 0.1% TBS-Tween (TBS-T) at room temperature for 1 hour. Unconjugated primary antibodies (CD3 and CD45; see Table 4, available as supplementary data at *Bioinformatics Advances* online, for clone and vendor information) were diluted to 5 µg/mL in a single cocktail with blocking buffer as diluent in sufficient volume to cover the section and incubated overnight at 4°C in a humidified chamber. The following day, tissue samples were washed in 0.1% TBS-T and TBS and then incubated with a cocktail of metal-conjugated secondary antibodies (1 µg/mL final concentration) for 1 hour. Sections were again washed with TBS-T and TBS before incubation overnight at 4°C in a cocktail of conjugated primary antibodies. The next day, slides were incubated with 500 hM iridium intercalator for 5 minutes (Standard BioTools) to visualize nuclei, followed by additional TBS washes. Finally, slides were briefly rinsed in DI water and dried with pressurized air ahead of IMC acquisition. The final antibody panel is described in Table 4, available as supplementary data at *Bioinformatics Advances* online.

Acquisition: IMC acquisition was performed on a Hyperion Imaging System with 400 Hz ablation after successful instrument QC (Standard BioTools). Multiple ROIs were acquired at a resolution of 1 µm for each tissue with data preprocessing completed using the commercial acquisition software (Standard BioTools).

Cell segmentation: Cell were segmented using the Mesmer model in DeepCell v0.12.2 (Greenwald *et al.* 2022). Mesmer expects an image with two channels as input—a ‘nuclear’ channel and a ‘cytoplasm’ channel—therefore, we used an average of the two DNA channels as the ‘nuclear’ channel and we used an average of seven cell lineage and membrane markers (CK19, SMA/Vimentin, CD45, CD3, CD15, HLA-ABC, and HLA-DR) for the ‘cytoplasm’ channel.

### 2.11 Segmentation quality check

To perform segmentation quality check, we selected five pairs of non-co-expressed proteins. CD3 (T cell) and CD20 (B cell); CK8/18 (luminal epithelial) and CK5 (basal epithelial); CD45 (pan-immune) and CK8/18; SMA (myoepithelial) and PanCK; GATA3 (luminal epithelial); and CD14 (basal epithelial).

We calculate the median expression for each protein over all the cells from each segmentation method, Ilastik + CellProfiler and Mesmer/DeepCell. We then count the number of cells from each segmentation method that shows expression greater than the protein median for both proteins in the protein pair. Finally, we express this count as a percentage over the whole dataset.

### 2.12 Batch correction study

We use the implementations of Scanorama and Harmony available from the scanpy external package (Wolf *et al.* 2018). We apply PCA on the full feature set and apply both methods on the top 50 principal components. The embeddings from both methods are used to cluster the cells using Leiden clustering with 100 nearest neighbours and resolution 1.0. Since it is important to ensure that not much of the biological information is lost during the batch correction process, we compare the methods to each other using a combination of batch correction and biology conservation metrics, available from the python package scIB (Luecken *et al.* 2022).

### 2.13 Projection on latent space and comparison with baseline

Only the Jackson-BC and Ali-BC datasets were included for this analysis as these share an antibody panel. We did this analysis in two ways: use the latent space learnt from the cells in Jackson-BC to generate low-dimensional cellular representations for cells in Ali-BC and vice versa. We will call the dataset that the latent space belonged to as the “reference dataset” and the dataset that is projected as the “query dataset”.

While carrying out this analysis, we needed to be mindful of the different numbers of background stains and samples present in both datasets as this would change the number of covariates expected by the model. In the case where the query dataset had more background stains than the reference dataset, we selected the stains that matched between the two. While in the case where the reference dataset had more background stains than the query dataset, we selected all the stains present in the query dataset while setting the value for all ‘missing’ stains to zero. In both scenarios, we used a random one-hot encoding for samples similar to Gayoso *et al.* (2021). This ensured that the number of total covariates input to the model was the same between the reference and query datasets. Details of how this analysis was carried out is outlined in Section 3.7.

## 3 Results

### 3.1 Exploring hyperparameter effects and unveiling cellular heterogeneity using multi-view integration with hmiVAE

**Figure 1:**
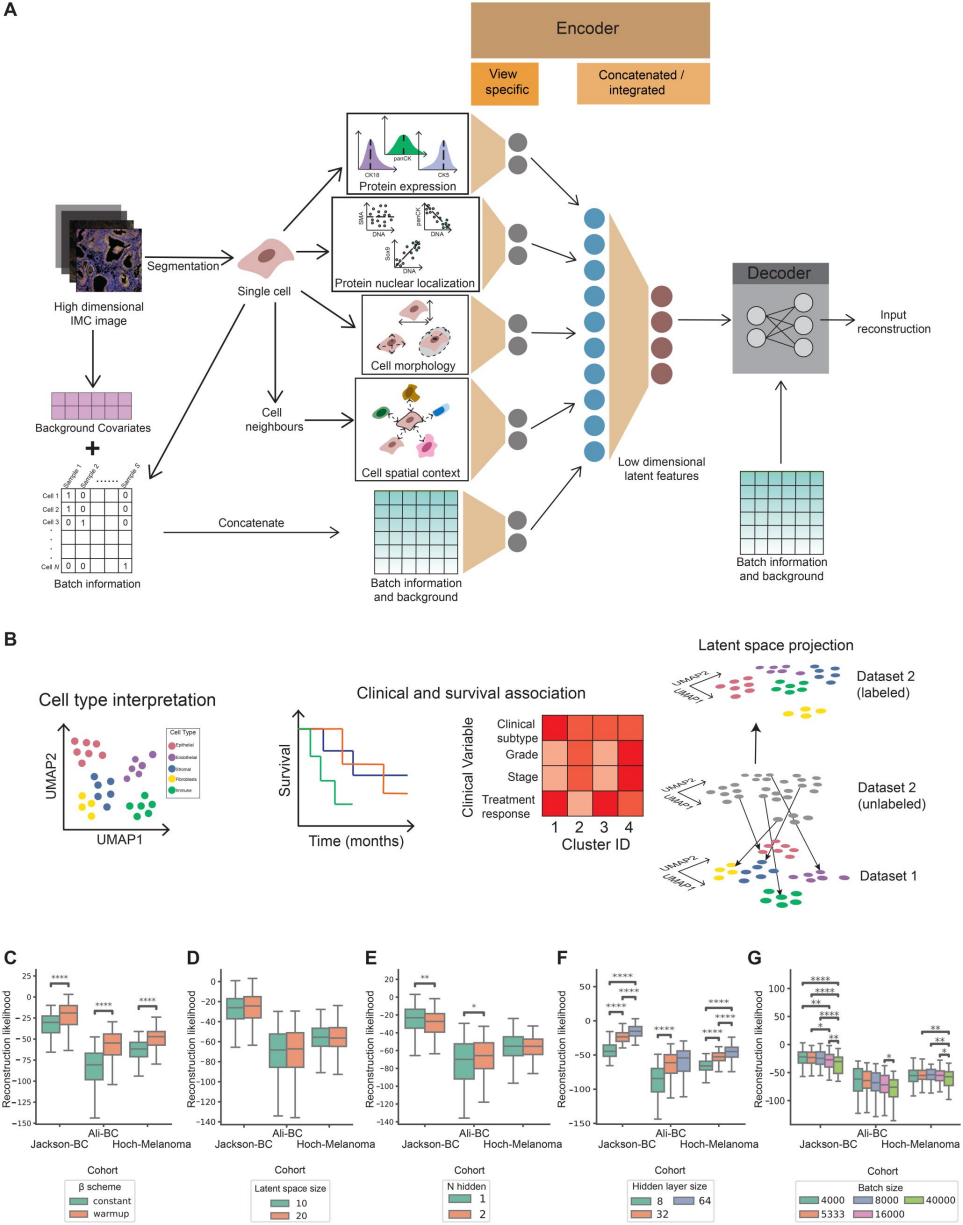
Multi-view integration of highly multiplexed imaging data using multi-modal variational autoencoders. (A) hmiVAE architecture. hmiVAE takes in inputs from four cell-specific views: protein expression, protein-nuclear co-localization, cell morphology, and cell spatial context. At the encoder stage, it first learns separate embeddings for each view which are then concatenated to learn a combined latent space. The decoder samples from this latent space to reconstruct the inputs. (B) Downstream tasks. The latent space and view-specific embeddings can be used for tasks such as cell type interpretation and association with clinical variables. We can also project cells from different datasets into the learnt latent space to find representations for these cells. (C–G) Hyperparameter tuning. Hyperparameter tuning results across datasets for beta scheme for KL-divergence weighting (constant or warm-up) (C), latent space size (D), number of hidden layers (E), size of hidden layers (F), and batch size (G). T-test statistical testing for hyperparameter comparisons and *P* values multiple tests corrected using Benjamini-Hochberg correction (*n = *600). *P*-value annotation: *0.01; *P* = .05; **0.001; *P* = .01; ***0.0001; *P* = .001; ****P = .0001. All n.s. comparisons are omitted.

We first developed a data processing pipeline to extract four views from IMC data: (i) mean protein expression, (ii) protein nuclear co-localization, (iii) cell morphology, and (iv) cell spatial context. We extract these views for each cell within an image. First, we extract mean protein cellular expression by taking the mean of the protein expression across all the pixels belonging to a cell. Second, we use the per-pixel protein expression counts and find the correlation between these and the mean nuclear score as a proxy for nuclear co-localization. Third, we compute the cell morphology features using image processing libraries in Python (Section 2). Finally, we summarize the expression counts, nuclear co-localization, and morphology for the *k* nearest neighbours of each cell to get the spatial context. Furthermore, highly multiplexed imaging data suffers from technical artefacts such as speckles resulting from non-specific antibody binding and uneven signal intensities across samples (Milosevic 2023). To control for such batch effects, we condition on sample specific identifiers and likely technical artefacts by including a one-hot encoding of cells belonging to each sample and computing covariates such as per-sample mean image intensity (Section 2). These views and covariates form the input to our highly multiplexed imaging variational autoencoder (hmiVAE). hmiVAE builds on a standard VAE architecture consisting of two neural networks, an encoder and decoder. The encoder takes in each view (expression, nuclear co-localization, morphology, and spatial context) and batch-specific information to first learn a separate representation for each view and then concatenates them to learn an integrated latent space. The decoder then samples from this integrated latent space to reconstruct each view (Fig. 1A and Section 2). The integrated latent space, as well as the view-specific embeddings can then be used for downstream tasks such as cell type interpretation and associations with clinical variables (Fig. 1B).

As tuning hyperparameters is a key aspect of successfully training any deep learning method (Yang and Shami 2020),we considered how varying a set of parameters affected the validation loss. Specifically, we selected (i) the number of hidden layers, (ii) the size of the hidden layers (i.e. hidden layer dimension), (iii) latent space dimensionality, (iv) batch size, and (v) the weight of the KL divergence term with a warmup and constant schedule (Section 2) and applied grid search over these to test how each impacted the reconstruction likelihood. During training, we separate our dataset into train and validation sets and implement an early stopping scheme to guard against overfitting (Section 2). We found that implementing a schedule on the weight resulted in better reconstruction than keeping it constant throughout training (Fig. 1C), a result also observed in other studies (Sønderby *et al.* 2016, Gayoso *et al.* 2021). We also noticed that while the dimensionality of the latent space and the number of hidden layers did not have a large effect on the reconstruction likelihood (Fig. 1D and E), a larger hidden layer size resulted in better reconstruction (Fig. 1F). Similarly, having a smaller batch size during training also resulted in better reconstruction (Fig. 1G). We trained the models with the best-performing hyperparameters for each dataset, Jackson-BC and Ali-BC which are breast cancer IMC datasets from Jackson *et al.* (2020) and Ali *et al.* (2020) respectively with epithelial and stromal marker panel, and Hoch-Melanoma which is a melanoma IMC dataset from Hoch *et al.*(2022) with an immune marker panel (Section 2, Table 1, available as supplementary data at *Bioinformatics Advances* online). The resulting latent spaces from hmiVAE can be used for downstream analysis (Gayoso *et al.* 2021, Lopez *et al.* 2022, Xu *et al.* 2022). Using Leiden clustering from the single-cell analysis Python library, scanpy (Wolf *et al.* 2018) (Section 2), we clustered the resulting latent spaces, we call these *integrated clusters* below and always refer to them as such throughout the text.

**Figure 2:**
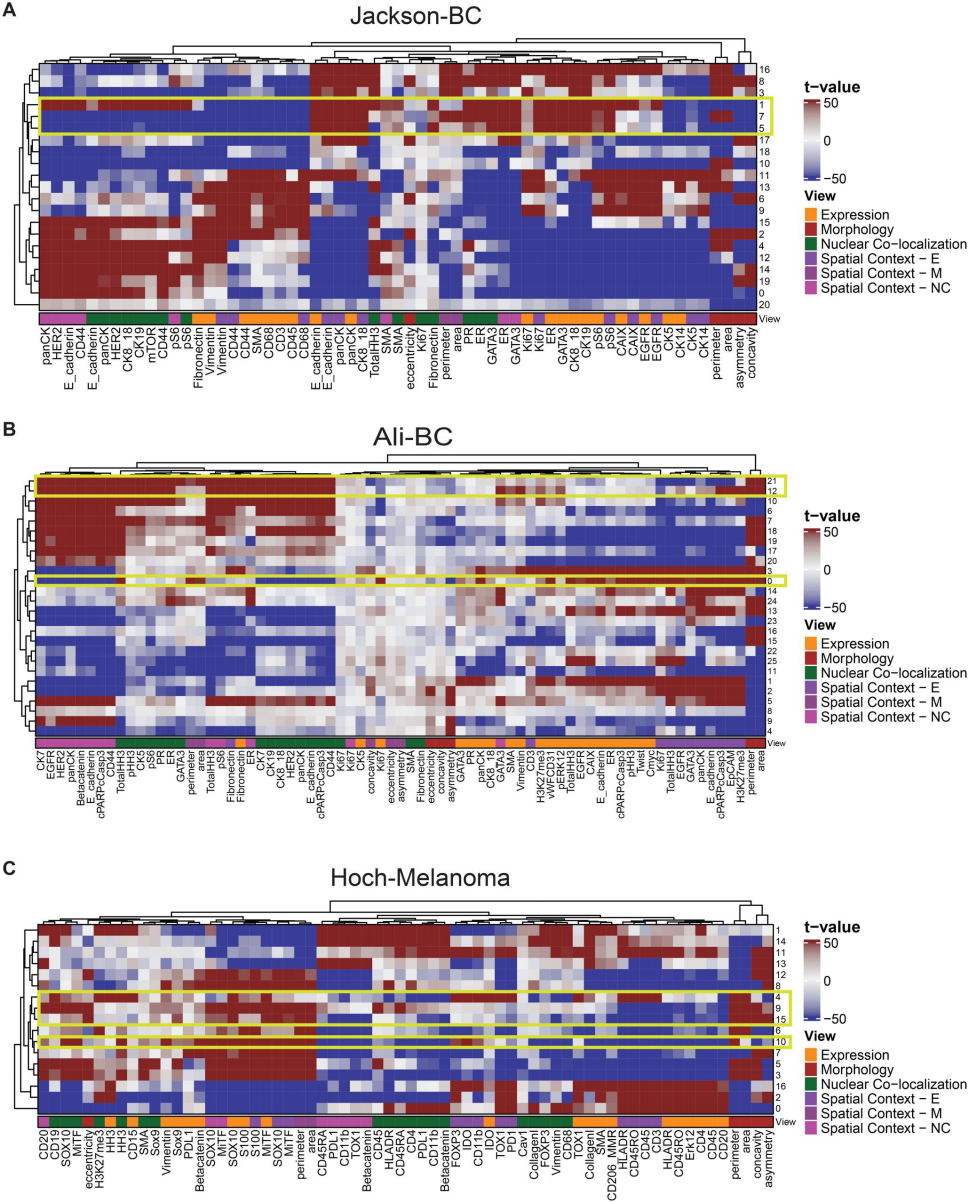
Ranking features across each view for integrated space clusters. Heatmaps showing the t-values for each feature representing their association with each integrated cluster in the dataset. Clusters in each dataset show differing patterns of within-cell protein expression, nuclear colocalization and morphology, and neighbourhood-cell protein expression, nuclear colocalization, and morphology. (A) Association of clusters found from the integrated latent space of hmiVAE run on Jackson-BC. (B) Association of clusters found from the integrated latent space of hmiVAE run on Ali-BC. (C) Association of clusters found from the integrated latent space of hmiVAE run on Hoch-Melanoma. Spatial context—E (neighbourhood-cell protein expression), Spatial context—NC (neighbourhood-cell protein nuclear co-localization), Spatial context—M (neighbourhood-cell morphology).

To quantify the influence of each view on the resulting cell clusters, we individually ranked the features corresponding to each view (Section 2) by the magnitude of their association with each integrated cluster (Section 2). Visualizing the top three features per cluster shows that for each dataset many clusters had similar average expression profiles but differed in the localization of different proteins or the context of their neighbouring cells (Fig. 2). In the Jackson-BC dataset, we find integrated clusters 1, 7, and 5, which represent epithelial cells as indicated by expression but cells in cluster 1 differ in the self and neighbourhood expression of the hypoxia marker CAIX and Epidermal growth factor receptor (EGFR) as well as the nuclear co-localization of HER2 (Fig. 2A, Fig. 19, available as supplementary data at *Bioinformatics Advances* online). In the Ali-BC dataset (Fig. 2B, Fig. 20, available as supplementary data at *Bioinformatics Advances* online), integrated clusters 12 and 21 both represent stromal cell clusters based on expression of Vimentin and Smooth muscle actin (SMA). However, they differ in terms of their spatial context: cluster 12 indicates stromal cells surrounded by apoptotic epithelial cells, given by cluster 12’s association with the expression of cPARP/cCasp3 and EpCAM in the neighbourhood, whereas cells in cluster 21 show a negative association with neighbourhood expression of cPARP/cCasp3 and EpCAM. Similarly, in the Hoch-Melanoma dataset, integrated clusters 4, 9, and 15 represent tumour cells, with cells in clusters in 9 and 15 having atypical morphology. Cluster 4 differs from others by the presence of a tumour suppressive environment indicated by the neighbourhood expression of markers for regulatory T cells (CD3, FOXP3, and TOX1) and macrophages (CD11b) (Fig. 2C, Fig. 21, available as supplementary data at *Bioinformatics Advances* online).

### 3.2 Expression-only embeddings improve cell type interpretation

Cell type annotation represents an important step in IMC data analysis workflow to provide insights into the cellular compositions of the tissues. Since this is commonly performed using the mean expression of proteins, we aimed to evaluate the efficacy of using the expression-specific cell representations from hmiVAE for this task by clustering over them using a standard community detection algorithm Leiden (Traag *et al.* 2019) and compared it to cell types found by clustering over the original mean protein expression values using the same algorithm, Leiden and the latest python implementation of a popular clustering method, FlowSOM (Van Gassen *et al.* 2015, Couckuyt *et al.* 2024) (Section 2).

**Figure 3:**
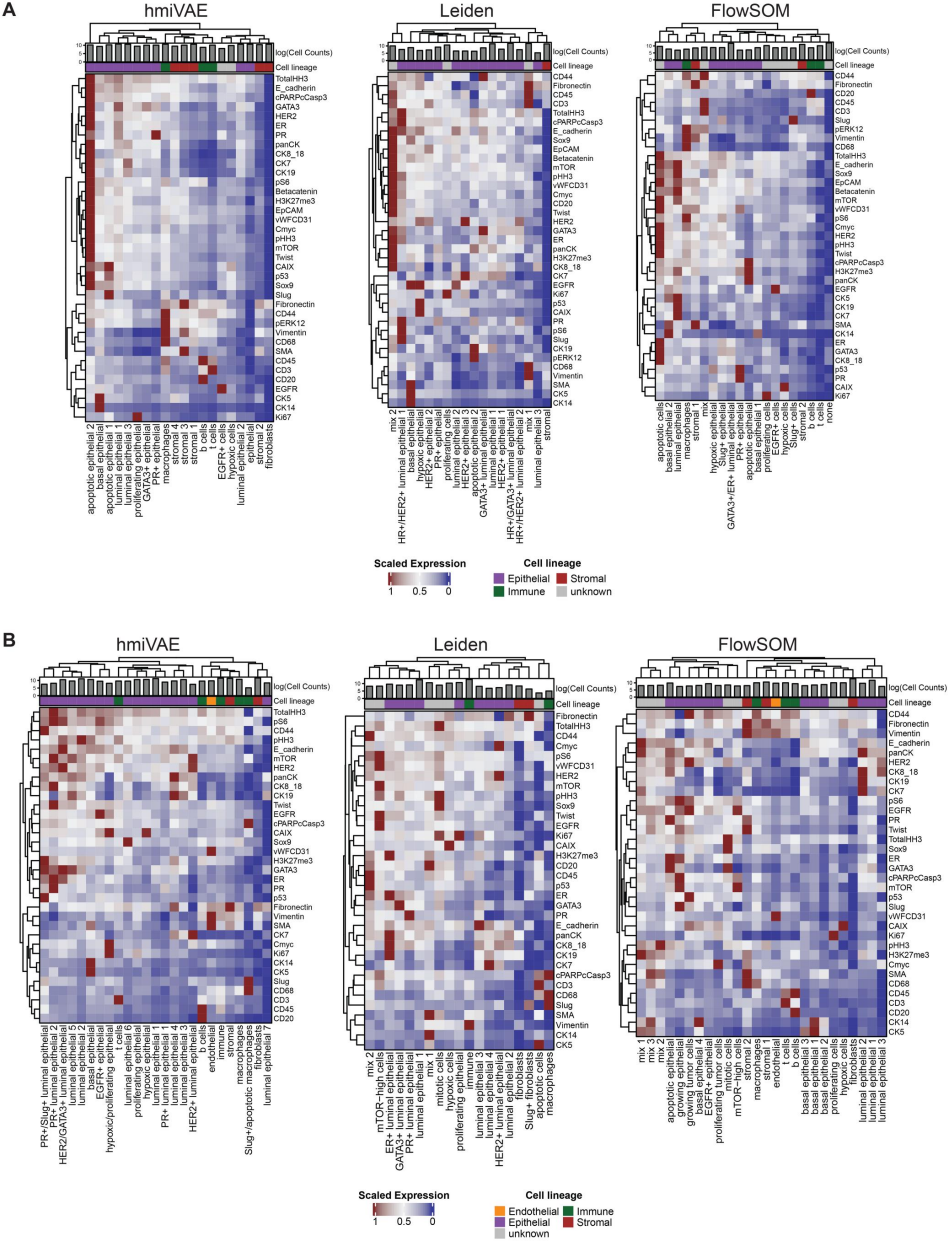
Cell type interpretation using expression-specific embeddings from hmiVAE and comparisons with Leiden and FlowSOM. Heatmaps showing expression values of expression-only features scaled to be between 0 and 1. Cell counts within each cluster are shown as logarithmized cell counts. (A) Cell types identified using hmiVAE vs. identified by Leiden and FlowSOM in Ali-BC using only the expression features set (*n = *536 883). Leiden finds basal epithelial and different epithelial and luminal epithelial clusters but no immune cell clusters. FlowSOM can find immune cell clusters corresponding to T cells, B cells, and macrophages, as well as luminal and basal epithelial cell clusters, however, one cluster from FlowSOM did not show high expression from any markers. Clusters from the expression-only embeddings from hmiVAE finds specific immune cell clusters corresponding to B cells, T cells and macrophages, as well as luminal and epithelial cell clusters. (B) Cell types identified using hmiVAE vs. those identified by Leiden and FlowSOM in Jackson-BC using only the expression features set (*n = *802 591). Like Ali-BC, majority of Leiden clusters correspond to luminal epithelial cells and two immune cell clusters, one corresponding to macrophages and one corresponding to a general immune subtype. FlowSOM finds multiple clusters showing a mix of markers, but it is also able to find specific immune clusters corresponding to B cells, T cells, and macrophages as well as clusters corresponding to endothelial cells, basal and luminal epithelial cells. As before, clusters from the expression-only embeddings from hmiVAE finds clusters corresponding to B cells, T cells and macrophages, as well as clusters corresponding to endothelial cells, basal and luminal epithelial cells.

For the Ali-BC and Jackson-BC datasets that contain markers for major cell type lineages within epithelial, stromal and immune compartments, hmiVAE and FlowSOM identified distinct clusters corresponding to CD3+ T cells, CD20+ B cells, and CD68+ macrophages (Fig. 3A and B). For Ali-BC, Leiden only found one cluster which shows expression of immune markers CD45, CD3, CD68 but also Fibronectin which indicates that this is a mixed cluster with largely immune cells, however, it was also able to find multiple clusters with high HER2 expression that were not found by either FlowSOM or hmiVAE (Fig. 3A). For Jackson-BC, Leiden found two clusters that corresponded to macrophages and general immune cells (Fig. 3B). Majority of clusters found by all methods represent luminal epithelial cells. In both datasets, hmiVAE identified a distinct basal epithelial cell cluster with clear expression of CK5 and CK14. Leiden clustering found one basal epithelial cell clusters in the Ali-BC while FlowSOM found two basal epithelial cell clusters, one of which shows a mix of other stromal and epithelial markers like SMA and E-Cadherin while the other only shows expression of CK14. In the Jackson-BC dataset, Leiden does not find any clusters corresponding to basal epithelial cells, whereas FlowSOM finds multiple basal epithelial cell clusters showing varying expression of CK14 and CK5 (Fig. 3). For both datasets, using original expression features, FlowSOM and Leiden, both resulted in many clusters which showed a mix of expression of multiple lineage markers and therefore could not be assigned to any one cell type. For Ali-BC, FlowSOM resulted in one cluster that did not show high expression of any markers and therefore could not be assigned to any cell types represented by the marker panels.

**Figure 4:**
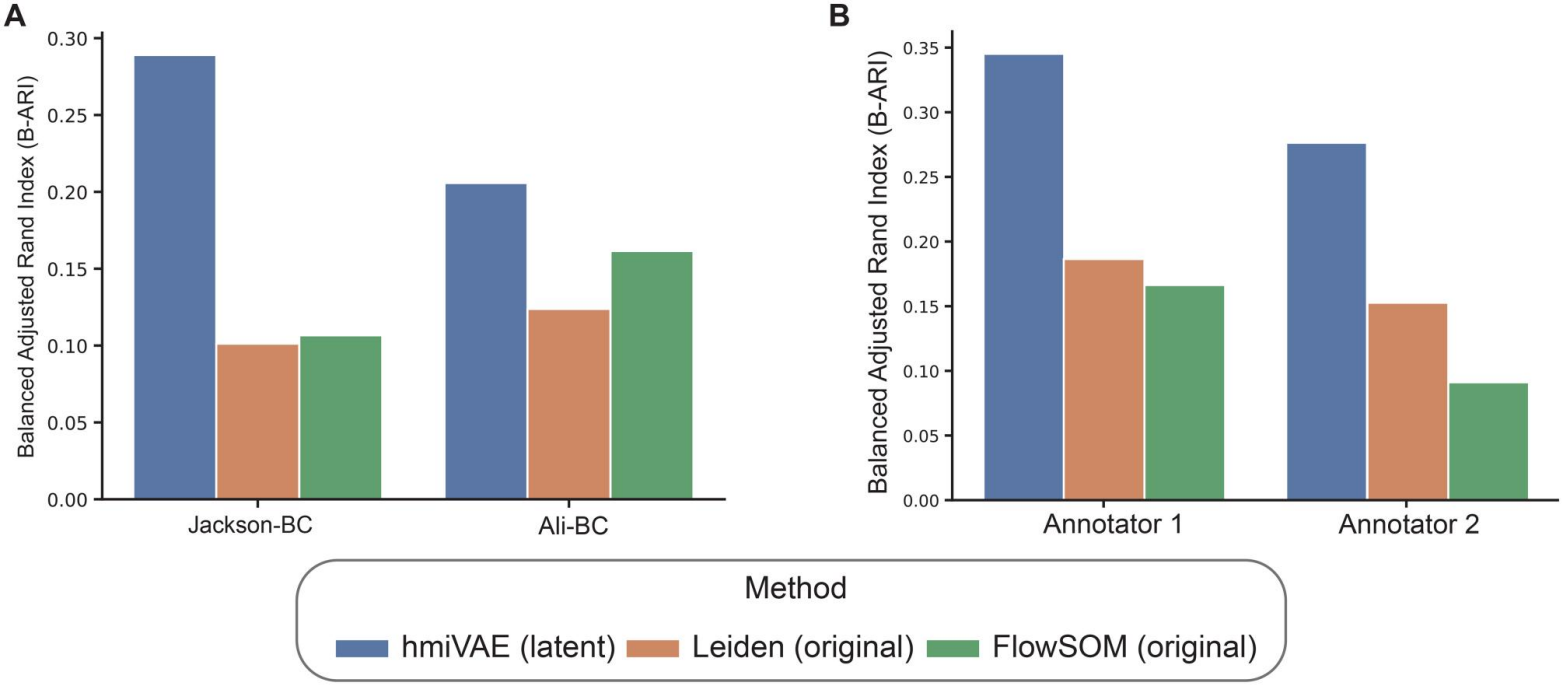
Comparisons of cell type clusters found by clustering on hmiVAE expression-only embedding features versus clustering on the original expression features. (A) Comparison to published annotations. When comparing to single-cell annotations from publications, clusters from hmiVAE’s expression-only embedding features were best in recapitulating the annotations for both datasets when compared to applying FlowSOM or Leiden on the original expression features. (B) Using manual ground truth annotations for Jackson-BC from two independent annotators, clusters found using the expression embedding of hmiVAE had higher balanced ARI scores indicating higher agreement of cell type label assignment with the annotators.

To further compare the clusters found by clustering on hmiVAE’s expression-only embedding features to those found by using FlowSOM and Leiden clustering on the original expression features, we treated the single-cell type assignments from Jackson *et al.* (2020) and Ali *et al.* (2020) as the ground truth labels for the Jackson-BC and Ali-BC datasets. For the Jackson-BC datasets, we also retrieved hand-annotated, manual single-cell annotations by two independent annotators for a subset of 500 cells from Geuenich *et al.* (2021) (Section 2). We contrasted clusters with ground truth using the Balanced Adjusted Rand Index (bARI), which is an improvement over the standard Adjusted Rand Index (Maan *et al.* 2024, Warrens and van der Hoef 2022). The bARI quantifies the similarity between two clusterings of data points and takes into account imbalances in cell type labels within the dataset (Maan *et al.* 2024). We found that for both datasets, hmiVAE achieves the highest bARI when compared to Leiden and FlowSOM indicating that clusters from the expression-only embedding features were better able to recapitulate the original, published cell type assignments than clusters from the original expression features (Fig. 4A). This is more interesting to note for the Ali-BC dataset since FlowSOM was used in the original publication for clustering (Ali *et al.* 2020). Similarly, in the comparisons with the manual, hand-annotated single cell labels, clusters using the expression embedding from hmiVAE, had a much higher bARI with both annotators as compared to using Leiden or FlowSOM clustering on the original expression features (Fig. 4B). Using the expression embedding clusters also resulted in higher F1-score across majority of cell type labels included in the subset of 500 cells (Figs 9 and 10). This highlights that the expression-only cell representations learnt by hmiVAE may allow for more accurate discernment of subtle immune and epithelial cell subtypes.

### 3.3 Clusters from integrated space are associated with clinical variables and patient survival

**Figure 5:**
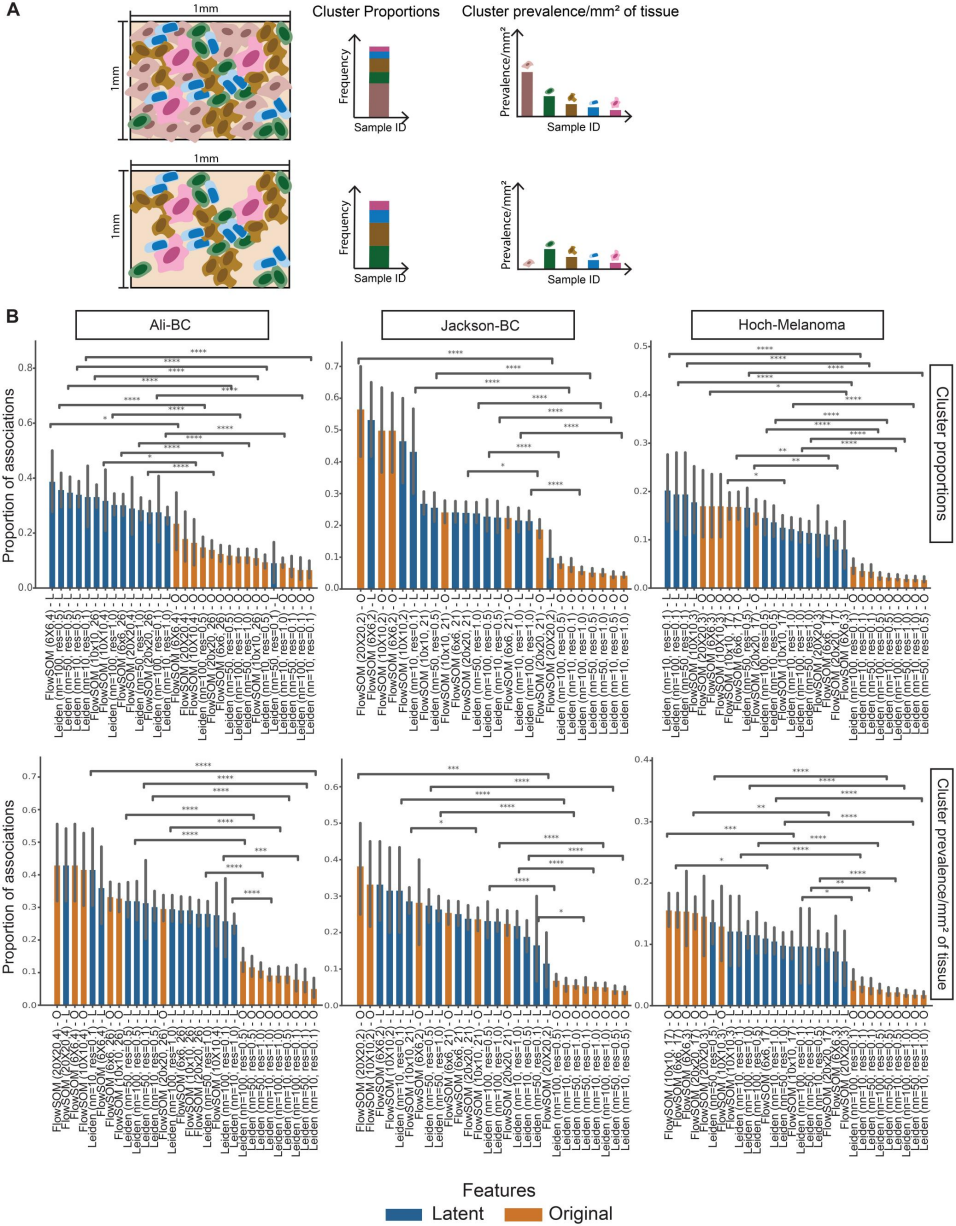
Clinical association with hmiVAE latent space vs original feature space. (A) Measures of cluster prevalence within a patient sample. We used two methods to describe cluster prevalence. (i) Cluster proportion—proportion of cells belonging to a given cluster within a patient and (ii) Cluster prevalence per mm^2^ of tissue or *tissue prevalence—*the abundance of cells from one cluster relative to the overall size of the tissue image. (B) Associations over all clinical variables using latent and original feature spaces across all clustering methods for all datasets. Parentheses next to FlowSOM show SOM grid size and number of clusters, respectively. L denotes the clustering method applied on the latent space, O denotes clustering method applied on the original feature space. Across all datasets, clustering over hmiVAE’s latent space resulted in a higher proportion of the resulting number of clusters to be associated with clinical variables regardless of clustering method. For Jackson-BC, Leiden (*n = *100, res = 0.1) and Leiden (*n = *50, res = 0.1) clustering using latent features resulted in only one cluster and so these were removed from this analysis. T-test statistical testing for clinical variable association and comparison (*n = *30 for Jackson-BC, *n = *18 for Ali-BC and *n = *41 for Hoch-Melanoma). Error bars indicate a 95% confidence interval. *P*-values are multiple tests corrected using Benjamini-Hochberg correction. *P*-value annotation: *0.01; *P* = .05; **0.001; *P* = .01; ***0.0001; *P* = .001; ****P = .0001. All n.s. comparisons are omitted.

Existing approaches have used the latent space of a deep unsupervised models to encode spatial information and marker expression to derive patterns that provide clinically meaningful differences between patient groups (Lopez *et al.* 2020, Bao *et al.* 2022, Tanevski *et al.* 2022, Paul and Madhumita 2022). Therefore, we examined whether clusters from the integrated latent space were associated with survival and clinical variables, such as stage, grade and cancer subtype (Table 1, available as supplementary data at *Bioinformatics Advances* online), as compared to clusters from the original, full feature space. We first established two metrics quantifying the presence of cells from a specific cluster within a patient’s tissue sample: (i) cluster proportions, which can be described as the relative abundance of a cluster compared to others, and (ii) cluster prevalence per mm^2^ of tissue, which can be seen as a measure of a cluster’s density within the tissue (Fig. 5A, Section 2). To ensure the differences in associations were due to selection of feature space instead of clustering method, we clustered using features from the integrated latent space as well as the full, original features using FlowSOM and Leiden. For Leiden, we used three different choices of nearest neighbours (i.e. 10, 50, 100) and three different choices for the resolution parameter (i.e. 1.0, 0.5, 0.1) (Section 2). For FlowSOM, we used three different sizes for the SOM grid (i.e. 6 × 6, 10 × 10, 20 × 20) and two different settings for the number of clusters (Section 2). We calculated both cluster prevalence metrics for clusters from each clustering method and using both prevalence measures, we computed their association with all clinical variables for each dataset. We also applied a Cox proportional hazards model to identify the clusters that were associated with survival after stratifying on cancer stage and grade (Section 2).

Across all datasets, we observed that for 90% of clustering choices a greater proportion of the clusters from the integrated latent space exhibited associations with clinical variables (Fig. 5B). The only exception is applying FlowSOM to the Hoch-Melanoma dataset, which showed that a greater proportion of clusters from the original feature space were associated with clinical variables than those from the integrated latent space (Fig. 5B). An investigation into the association of clinical variables with the latent features learnt by hmiVAE revealed multiple features across datasets that were associated with different clinical variables especially disease grade and subtype (Figs 11 and 12, available as supplementary data at *Bioinformatics Advances* online). In the Ali-BC dataset, we found a latent feature, 19, which was associated with the invasive lobular carcinoma subtype of breast cancer (Fig. 11A, available as supplementary data at *Bioinformatics Advances* online). We noticed that independent latent variables were associated with the BRAF and NRAS mutations in the Hoch-Melanoma dataset (Fig. 11B, available as supplementary data at *Bioinformatics Advances* online). In the Jackson-BC dataset, latent features 7, 10, and 12 showed associations with Tamoxifen treatment response (Fig. 12B, available as supplementary data at *Bioinformatics Advances* online). Overall, our findings suggest that the integrated latent space learned by hmiVAE may provide a more robust representation of cellular phenotypes associated with clinical variables compared to the raw, original features.

**Figure 6:**
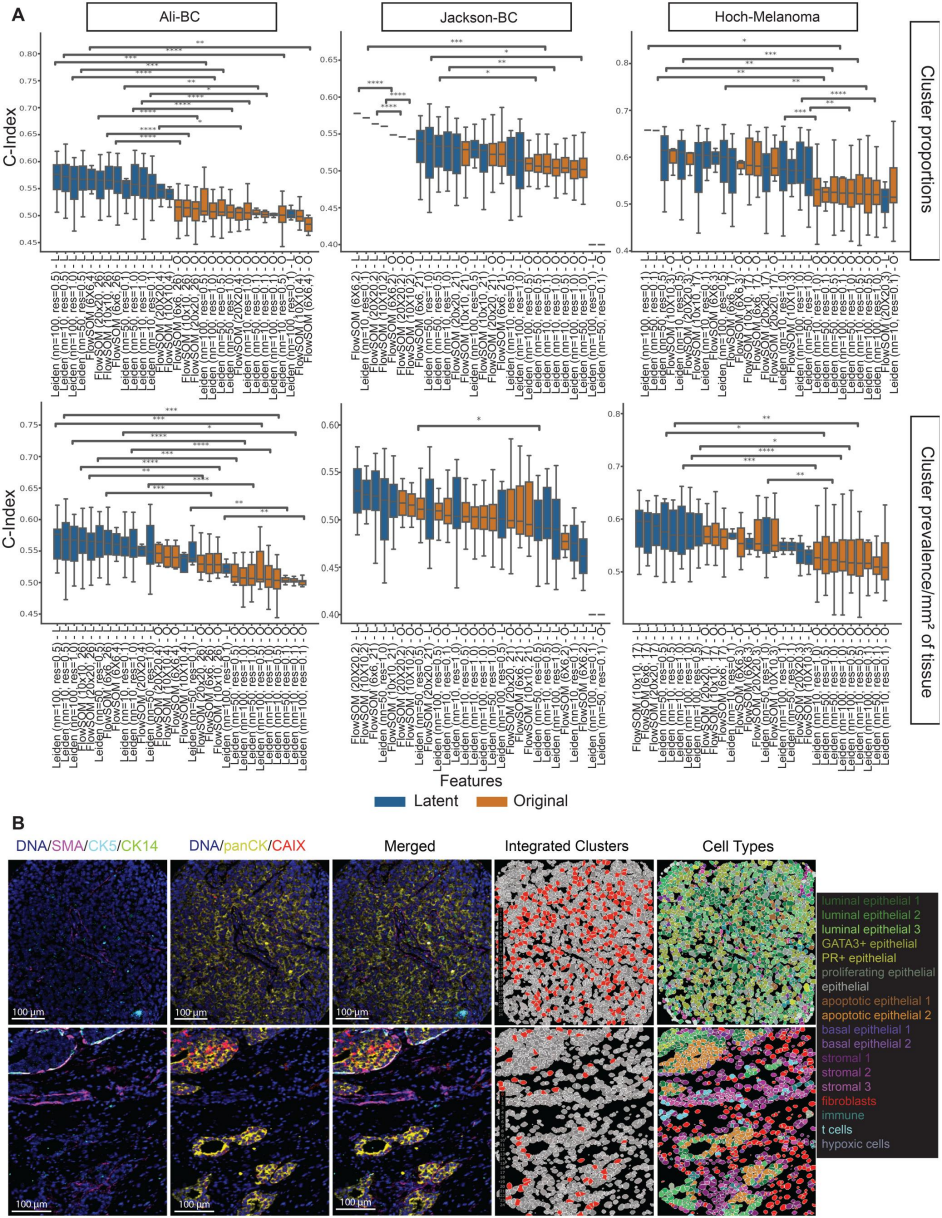
**Survival analysis with hmiVAE latent space vs original feature space**. (A) Concordance index from CoxPH models for all clustering methods using latent space vs original features. Parentheses next to FlowSOM show SOM grid size and number of clusters, respectively. L denotes the clustering method applied on the latent space, O denotes clustering method applied on the original feature space. Clusters from the latent space learned by hmiVAE show higher concordance in comparison to clusters from the original feature space using both metrics of cluster prevalence across all datasets. For Jackson-BC, clustering using latent features with Leiden (*n = *100, res = 0.1) and Leiden (*n = *50, res = 0.1) resulted in only one cluster and so these were removed from this analysis. T-test for statistical comparisons and p-values are multiple tests corrected using Benjamini-Hochberg correction. *P*-value annotation: *0.01; *P* = .05; **0.001; *P* = .01; ***0.0001; *P* = .001; ****P = .0001. All n.s. comparisons are omitted. (B) IMC images from the Ali-BC dataset. Images showing DNA, panCK, CAIX, SMA, CK5 and CK14 expression, as well as cell masks coloured by cell type and hmiVAE integrated space cluster ID for a patient with low number of cells belonging to integrated space cluster 0 (bottom) and a patient with high number of cells belonging to integrated space cluster 0 (top).

To quantify the association between cluster prevalence and patient survival outcomes, we evaluated the concordance indices of Cox proportional hazards (CoxPH) models applied to clusters obtained as described above while controlling for disease stage and grade. We observed that the clusters from hmiVAE’s integrated latent space consistently showed the highest concordance indices across all datasets regardless of choice of clustering method, indicating better associative performance in survival analysis (Fig. 6A). Furthermore, the improved performance of clusters from hmiVAE’s integrated latent space was statistically significant compared to directly clustering the original feature set, for both metrics of cluster prevalence. This significance was consistent across all datasets, suggesting that the integrated latent space learned by hmiVAE captures meaningful biological variation associated with patient outcomes, further supporting the robustness of our model.

**Figure 7:**
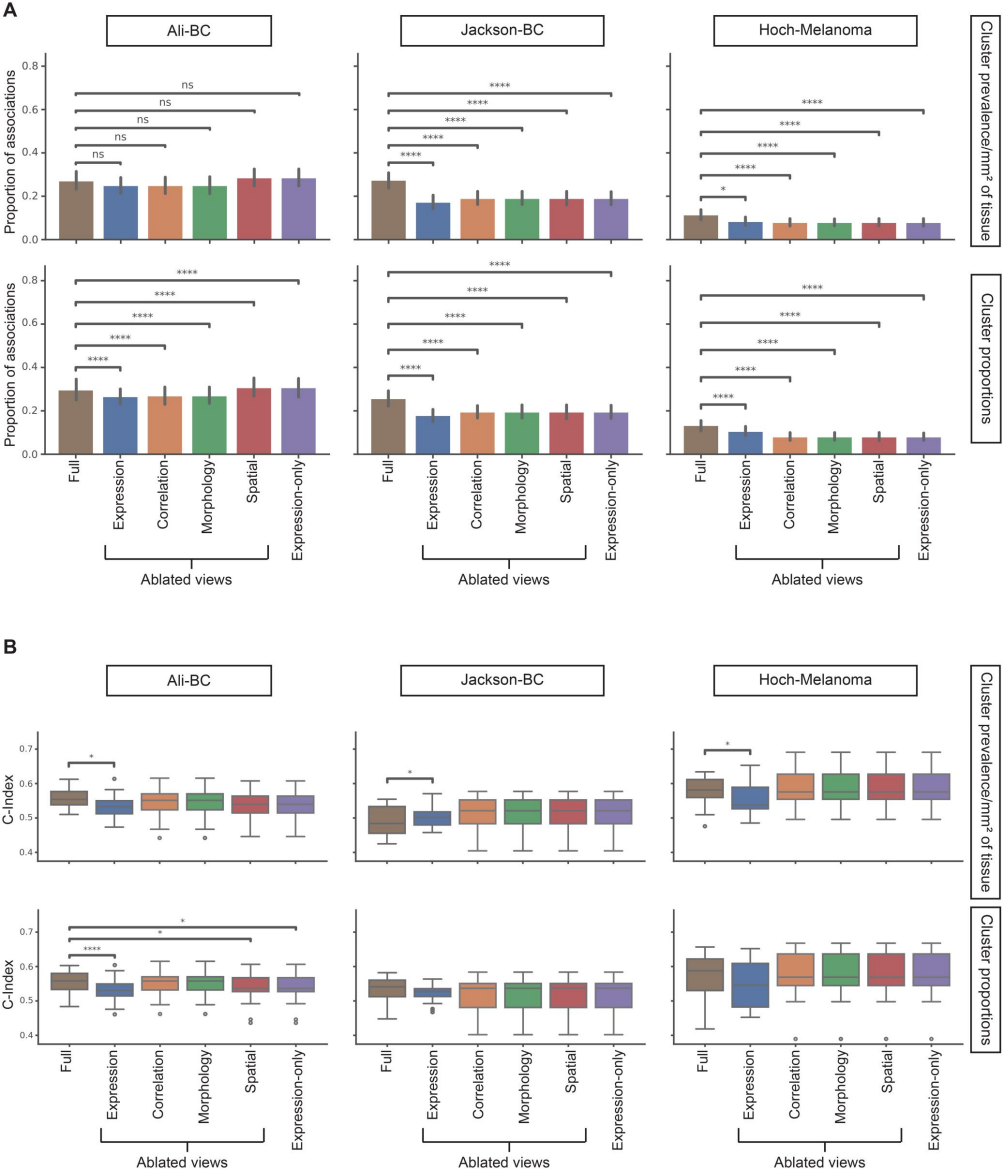
Ablation study. Associations of clinical variables (A) and concordance index for CoxPH models (B) using clusters from the latent space of models with each view—expression, nuclear co-localization, morphology, and spatial context—ablated and comparing with training hmiVAE with only expression features. T-test for statistical comparison and *P*-values are multiple tests corrected using Benjamini-Hochberg correction. For clinical variables, *n = *30 for Jackson-BC, *n = *18 for Ali-BC and *n = *41 for Hoch-Melanoma. Error bars indicate a 95% confidence interval. *P*-value annotation: *0.01; *P* = .05; **0.001; *P* = .01; ***0.0001; *P* = .001; ****P = .0001. All n.s. comparisons in (B) are omitted.

We next interpreted the biological features of the clusters with significant hazards ratios for both cluster prevalence measures in Ali-BC and Hoch-Melanoma (Figs 4–7, available as supplementary data at *Bioinformatics Advances* online). In the Ali-BC dataset, hmiVAE found a grade-associated cluster (*integrated cluster 0*) whose higher prevalence was significantly associated with higher risk based on a Cox proportional hazards model for both cluster proportion and tissue prevalence when stratified on cancer stage only (Fig. 13B, available as supplementary data at *Bioinformatics Advances* online). For both cluster prevalence metrics, this cluster showed high t-values for associations with clinical variables such as grade 3, IDC histological type and negative ER status. According to marker expression, this cluster consists of luminal epithelial cells expressing the hypoxia marker, CAIX, and the proliferation marker, Ki67. Given the depletion of myoepithelial cells (expressing SMA, CK5, or CK14) in this cluster and its spatial context (Fig. 2B), we hypothesized it may represent the hypoxic tumour core (Gorringe and Fox 2017). To investigate this further, we examined images from patients with high and low presence of integrated cluster 0 in their tissue sample (Fig. 6B;  Fig. 12A, available as supplementary data at *Bioinformatics Advances* online). We found that samples with a high presence of integrated cluster 0 had high expression of the tumour cell marker panCK and hypoxia marker CAIX, with little to no expression of SMA, CK5, or CK14. Also, in the few regions that showed expression of SMA, the luminal epithelial cells were not surrounding the tumour cells. On the other hand, for patients with a low number of integrated cluster 0 cells, samples had high expression of SMA, CK5 and CK14, low expression of panCK or CAIX, and the luminal epithelial cells were surrounded by myoepithelial cells (Fig. 6B). This spatial pattern supports our hypothesis that cells belonging to integrated cluster 0 found by hmiVAE might belong to the tumour core.

**Figure 8:**
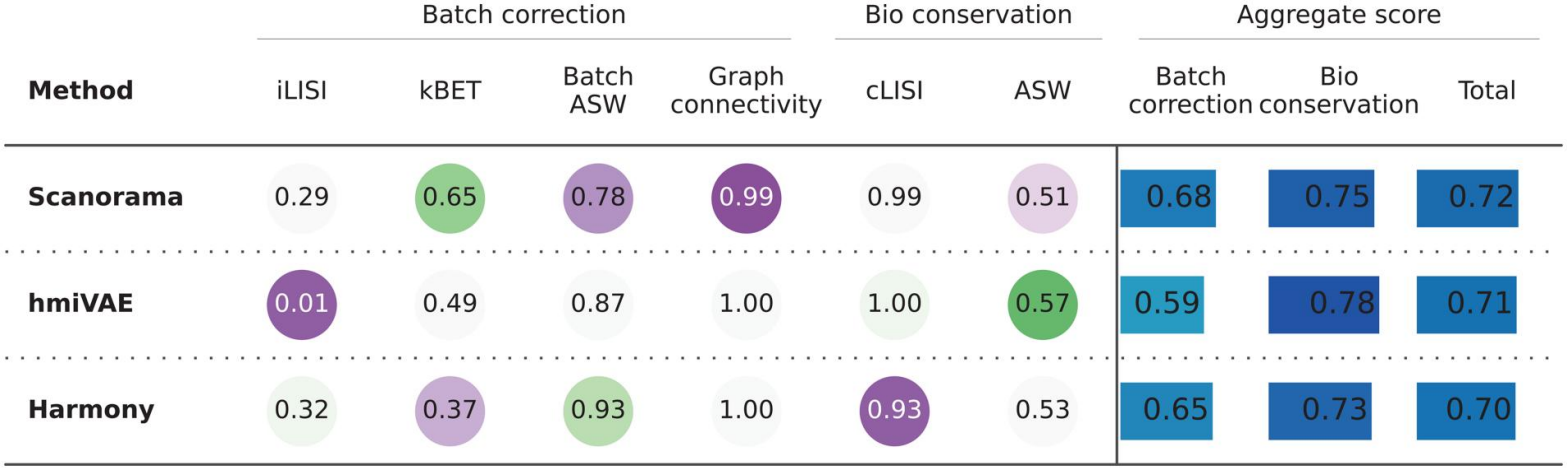
Batch correction and biological conservation benchmarking comparison between hmiVAE, Scanorama, and Harmony.

In the Hoch-Melanoma dataset, *integrated cluster 10* was significantly associated with survival for both measures whereas the hazard ratio for *integrated cluster 15* was only significant using cluster proportions (Figs 5 and 7, available as supplementary data at *Bioinformatics Advances* online). Using both metrics of cluster prevalence, integrated cluster 10 high t-values for association with clinical variables such as partial disease treatment response at 3 months and stage 4 melanoma, while integrated cluster 15 showed high associations with clinical variables such as acral lentiginous melanoma subtype and NRAS mutation. We found that integrated cluster 10 represented tumour cells expressing and surrounded by cells expressing IDO, which has been implicated in degrading T cell function (Uyttenhove *et al.* 2003). These tumour cells were also surrounded by T regulatory cells (given by the neighbourhood expression of FOXP3) and macrophages (denoted by the neighbourhood expression of CD11b), both of which are canonical markers of a immunosuppressive microenvironment (Tie *et al.* 2022). In contrast, integrated cluster 15 represented tumour cells without such markers in their neighbourhood (Fig. 2C). A patient sample that had a high number of cells belonging to both clusters exhibited a mixed pattern with cells closer to IDO expressing cells belonging to integrated cluster 10 and those away from these cells belonging to integrated cluster 15 (Fig. 8, available as supplementary data at *Bioinformatics Advances* online). We note that such spatially informed patterns would be difficult to identify using mean protein expression alone, which highlights the importance of incorporating neighbourhood information to identify cell states.

Our analysis demonstrates that the integrated clusters derived from the latent space learnt by hmiVAE framework exhibit significant associations with various clinical variables and patient survival outcomes across multiple datasets, highlighting their potential as robust biomarkers for clinical stratification. Further investigations with larger datasets and additional statistical tests may provide deeper insights into the significance of these findings. Our results demonstrate the value of incorporating multi-view information and leveraging deep learning approaches for more accurate and clinically relevant insights into cellular phenotypes and their associations with patient outcomes in complex biological systems.

### 3.4 Quantifying the contribution of each view to model performance

We next investigated how each view impacts hmiVAE’s integrated latent space and subsequent associations with clinical outcomes. We ablated each view from training by setting the weight of the ablated view in our loss function to zero (Section 2) and trained as before using the best combination of hyperparameters determined from the full model (Table 2, available as supplementary data at *Bioinformatics Advances* online). We also trained a variant that used only the expression features by setting all weights except the expression weight to 0 (Section 2). The reconstruction of the ablated view is greatly impacted in the respective models indicating successful ablation (Fig. 15, available as supplementary data at *Bioinformatics Advances* online). We then clustered the latent space from each ablation model and expression-only model, as before. Using the two cluster prevalence metrics described earlier (proportion and prevalence), we associated each with clinical and survival data stratifying on disease stage and grade as before (Section 2).

Across the Hoch-Melanoma and Jackson-BC datasets, we observed that the inclusion of all four views resulted in a greater proportion of clusters showing significant associations with clinical variables using both metrics for cluster prevalence (Fig. 7A). The ablated view models and expression-only models resulted in a similar proportion of clusters with associations to clinical variables for these two datasets. This demonstrates that the full multi-view feature set and latent space contribute the most information for finding associations with clinical outcomes. However, for the Ali-BC dataset, the expression-only and the spatial context ablated models resulted in a similar proportion of clusters with significant associations with clinical variables as the full model, indicating that for this dataset, the expression features alone may be the most informative for finding relationships with clinical variables (Fig 7A).

Next, we investigated how view ablation affects the association of the latent spaces to patient survival. For the Ali-BC dataset, we observed that the clusters from the latent space of the full model had higher concordance indices for both cluster prevalence metrics (Fig. 7B). In contrast, for the Jackson-BC and Hoch-Melanoma datasets, clusters from the integrated latent space had approximately the same concordance index as when all views except expression were ablated. There was a large decrease in concordance index when expression was ablated, indicating that the expression features contain the most signal for survival outcomes in these datasets (Fig. 7B). Overall, these ablation experiments show on average improved association with clinical and survival when using an integrated latent space across all views, but that this may be dataset and/or task dependent, requiring future practitioners to also perform ablation experiments to assess optimal model design.

### 3.5 Robustness of feature space to different segmentation algorithms

The features we include in our study are summarized to the level of single-cells using masks from the segmentation algorithm used in the respective original publications of the Jackson-BC, Ali-BC, and Hoch-Melanoma datasets. This raises the question of how the choice of segmentation algorithm impacts the features used as input to the model and subsequent results on embeddings downstream. To investigate this, we segmented the Ali-BC dataset with two segmentation algorithms and repeated the clinical and survival association analysis for both.

Specifically, we took the original segmentation masks from Ali *et al.* (2020) which were generated using the pixel classification tool Ilastik (Berg *et al.* 2019) and the cell image analysis tool CellProfiler (Carpenter *et al.* 2006, McQuin *et al.* 2018). We then generated a second set of single-cell masks using the Mesmer model from DeepCell (Greenwald *et al.* 2022), as previously performed by Lee *et al.* (2025). We call the dataset with the original cell segmentations, ‘Ali-BC’ and the dataset with the DeepCell cell segmentations, ‘Ali-BC-DC’, in text and figures. To compare the difference in segmentation quality between these two methods, we follow a strategy performed by STARLING (Lee *et al.* 2025) by comparing the expression of five non-co-expressed proteins pairs (i.e. CD3-CD20, CK8/18-CK5, CD45-CK8/18, SMA-PanCK, GATA3-CK14) over the whole dataset (Section 2). We observed no significant differences between these two segmentation methods using this strategy (Fig. 22, available as supplementary data at *Bioinformatics Advances* online). We trained hmiVAE on the new Ali-BC-DC dataset as above to find optimal hyperparameters (Table 2, available as supplementary data at *Bioinformatics Advances* online) then applied Leiden clustering and summarized cluster prevalence using the two metrics described above. We then associated each cluster’s prevalence with clinical variables and survival outcomes as before.

We observed that the Mesmer segmentation results in a slightly higher proportion of clusters with significant associations with clinical variables. However, this result was only statistically significant for the cluster prevalence per mm^2^ of tissue metric. For survival outcomes, the Ilastik + CellProfiler segmentation had a slightly higher association, however, the median concordance index for both segmentations was similar (Fig. 16, available as supplementary data at *Bioinformatics Advances* online). This result shows that the method of segmentation does not impact relationships found between the prevalence of the resulting clusters and clinical variables and survival outcomes, suggesting that the learnt latent features are robust to different segmentation algorithms.

### 3.6 Evaluating batch correction with a multi-slide IMC dataset

Many studies using highly multiplexed imaging, including the datasets used above, use tissue microarrays that collate multiple tissue regions onto a single slide to minimize batch effects. This in turn precludes our ability to accurately assess the batch correction ability of different algorithms, which we do expect to contribute significant variation when samples are placed on different slides and acquired at different times (Milosevic 2023). Such a dataset would require careful batch effect removal to ensure that the latent space does not learn technical artefacts. Therefore, we generated Imaging Mass Cytometry for five biopsy samples from patients with pancreatic adenocarcinoma (PDAC), using an immune/stromal/tumour focussed panel. Each image is acquired separately on a different slide and at different times (Section 2), resulting in true slide-to-slide batch variation.

We tested batch effect removal in the latent space learnt by hmiVAE and compared it to two batch correction methods for single-cell data: Scanorama (Hie *et al.* 2024) and Harmony (Korsunsky *et al.* 2019) (Section 2). We quantified both batch correction and biology conservation in line with previous studies (Gayoso *et al.* 2021, Luecken *et al.* 2022, Cao and Gao 2022) across a range of metrics. We observed that no method performs the best across all metrics, although Scanorama achieved the highest total score (Fig. 8). hmiVAE scored the highest for biological conservation but the lowest for batch correction performing particularly poorly for the iLISI metric but similarly to Scanorama and Harmony for all other metrics. Overall, our results indicate that there is always some loss of biological information during batch correction, so no method can achieve the highest score for both.

### 3.7 Latent space projection with hmiVAE

**Figure 9:**
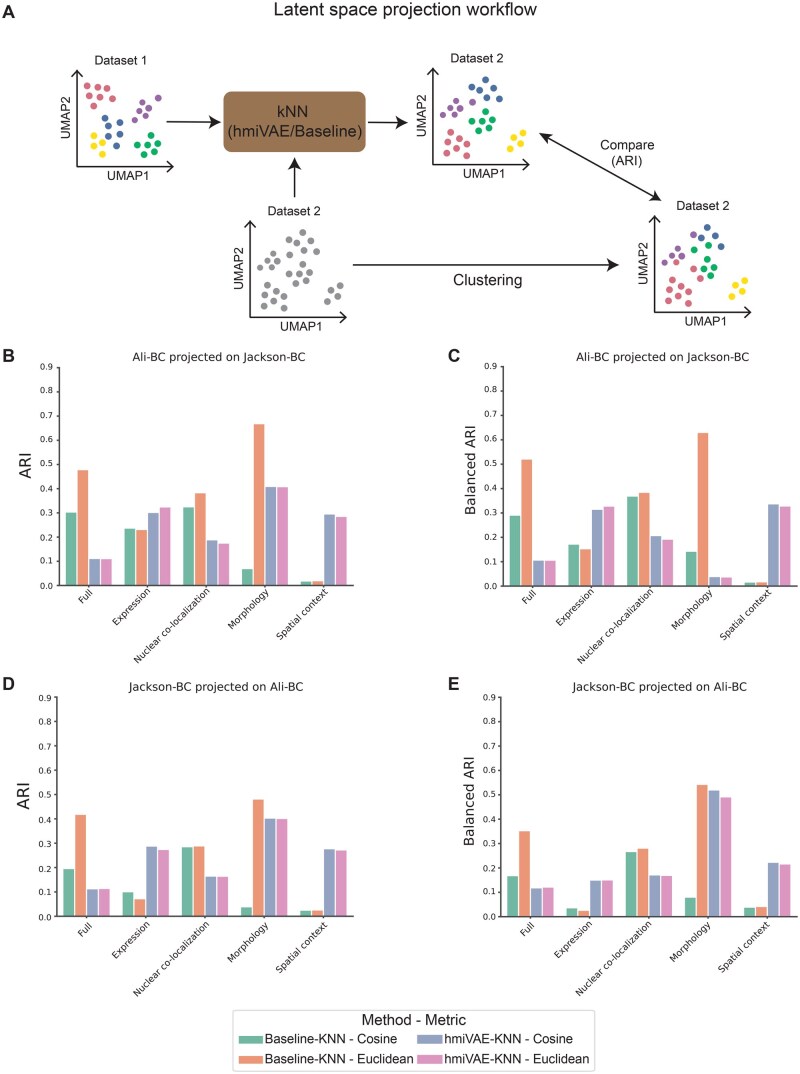
Latent space projection analysis with hmiVAE and comparisons with baseline KNN. (A) Schematic showing latent space workflow. A KNN model is trained using (i) labels from latent space or view-specific embeddings of hmiVAE trained on Dataset 1 (hmiVAE-KNN) or (ii) labels from clustering directly on the full or view-specific features set from Dataset 1 (baseline-KNN). Labels for an independent dataset, Dataset 2, are generated from this trained KNN model. The KNN clustering results are compared to cluster labels from ground truth Dataset 2 using adjusted rand index (ARI) or balanced ARI. (B and C) Projection results for Jackson-BC as Dataset 1 and Ali-BC as Dataset 2 using both cosine and Euclidean distance metrics for KNN reported as ARI (B) and balanced ARI (C). D and E, Projection results for Ali-BC as Dataset 1 and Jackson-BC as Dataset 2 using both cosine and Euclidean distance metrics for KNN reported as ARI (D) and balanced ARI (E). Full, integrated space or full features set.

The latent space of a VAE captures the salient features that explain the variation in high dimensional data (Kingma and Welling 2014, Suzuki and Matsuo 2022). Therefore, given shared original features in the high dimensional space, we sought to investigate how similar and transferable the learnt latent space and feature embeddings were between two IMC datasets. To do so we used the Ali-BC and Jackson-BC datasets, which share an antibody panel. We trained hmiVAE on one dataset (reference dataset) and used it to generate latent space and view-specific cell representations for the other dataset (query dataset). We did this by first clustering cells from the reference dataset using their latent space representations or view-specific embeddings and assigning them to a cluster ID. These latent cell representations or view-specific embeddings and their associated cluster IDs were then used to train a K Nearest Neighbour model (KNN) (Cover and Hart 1967), we call this the hmiVAE-KNN model. We then used the hmiVAE model trained on the reference dataset to generate latent space representations and view-specific embeddings for cells in the query dataset and these were then fed into the trained KNN model to generate cluster IDs. This approach was compared to a baseline model where we clustered the cells from the reference dataset using their original features—full (including features from all views), expression, nuclear co-localization, morphology, and spatial context—and trained the KNN model using the resulting cluster IDs, we call this the baseline-KNN model. We then used this baseline-KNN model to generate cluster IDs for cells in the query dataset using their original features (Section 2). For ground truth comparisons in the case of using the latent space learnt by hmiVAE, we compared the labels for the query dataset from the hmiVAE-KNN model to the cluster IDs resulting from training hmiVAE on the query dataset and clustering the cells to assign cluster IDs. In the baseline-KNN case, we used cluster IDs generated by directly clustering the cells from the query dataset using their original features for comparison. We applied KNN using both cosine and Euclidean distance metrics and compared clustering results to ground truth (hmiVAE or baseline) using the Adjusted rand index (ARI) and a balanced ARI score which adjusts the ARI score to weight underrepresented and overrepresented classes similarly (Maan *et al.* 2024) (Fig. 9A).

Our analysis revealed distinct performance trends based on the feature sets used. ARI and balanced ARI also did not differ much except in the case of morphology-only features for hmiVAE-KNN especially when projecting cells from Ali-BC onto the morphology-only embedding learnt from Jackson-BC (Fig. 9B and C). Since balanced ARI tries to weigh all groups present in the dataset similarly, this indicates that there may be some rare morphologies present in the Ali-BC dataset, not seen in the Jackson-BC dataset, since the Ali-BC dataset comprises of cells from patients with primary invasive carcinoma (Curtis *et al.* 2012, Ali *et al.* 2020) while patients in the Jackson-BC dataset were not selected for any clinical or histological features (Jackson *et al.* 2020). We notice that the baseline-KNN model consistently achieved higher ARI and balanced ARI scores when applied to the full feature set and nuclear co-localization features in both datasets. While hmiVAE exhibited superior performance in clustering when leveraging expression and spatial context features (Fig. 9). Furthermore, the choice of distance metrics did not influence the projection capability of the features learnt by hmiVAE but did when using the original features, especially for the full feature set and morphology features, which is expected as the latent space of a VAE is regularized and therefore the effect of the changes in magnitude is mitigated between features. This delineation underscores the importance of considering which features are being used for projection. Latent space projection is considered a beneficial potential extension for applications in biology (Ramirez Flores *et al.* 2023), but our study demonstrates that this might not be so straightforward when projecting cells from a different dataset onto a latent space or view-/modality-specific embedding.

## 4 Discussion

Here, we have investigated the use of VAEs for multi-view modelling of IMC data. We follow the standard analysis pipeline of other IMC studies, where clustering is performed on all cells of the dataset using expression features, and the prevalence of these clusters are subsequently associated with clinical outcomes (van Maldegem *et al.* 2021, Danenberg *et al.* 2022, Moldoveanu *et al.* 2022). Our approach revealed integrated clusters corresponding to diverse cellular phenotypes characterized by subtle variations in protein expression, subcellular localization, and microenvironmental context. Notably, our findings highlight the necessity of incorporating nuclear co-localization, morphological features, and spatial information to capture the complexity of cellular states more accurately than from using expression alone. We show that the view-specific embeddings and integrated latent representations for each cell led to the identification of clusters which had strong associations with clinical variables and patient survival, as well as improved performance for common downstream tasks such as cell type annotation. We note that while this investigation followed the standard approach of extracting single cells and identifying cell-level phenotypes, other studies have improved the performance on downstream tasks by including pixel-level annotations and thereby increasing robustness of the features extracted (Liu *et al.* 2023). This approach could be used to adapt our input features, such as pixel-level protein expression and spatial context, and to investigate the effects on the learnt representations and the performance on downstream tasks.

Further, in this study, our exploration of hyperparameters in the hmiVAE architecture unveiled effects on reconstruction performance, emphasizing the importance of dynamically adjusting the weight of the KL-Divergence term and optimizing hidden layer size and batch size for superior reconstruction. Leveraging these insights, we identified optimal hyperparameter configurations for each dataset, maximizing reconstruction likelihood to construct integrated clusters representative of the underlying cellular heterogeneity. However, we considered only a subset of possible hyperparameters that could be expanded in future work to contain the learning rate known to have a big impact on model performance (He *et al.* 2019). In our case, it could also be beneficial to vary the size of the view-specific embeddings given the differences in the number of features for each input view. We note that although differences in the number of cells between datasets, e.g. Hoch-Melanoma (989 404) versus Ali-BC (536 883), did not significantly impact the time taken by each epoch, a full hyperparameter tuning run can take 1–2 days depending on how many hyperparameters are considered. We also incorporate training schemes such as early stopping which stops training once there is no further improvement in the validation loss which saves training time, therefore, if a higher number of epochs is needed to reach this point, then training time would also increase.

Additionally, we only investigate the application of VAEs for multi-view integration given their popularity for this task (Stark *et al.* 2020, Gayoso *et al.* 2021, Minoura *et al.* 2021, Lynch *et al.* 2022, Cao and Gao 2022, Athaya *et al.* 2023). However, studies could also investigate the application of deep learning architectures that incorporate graph neural networks (Scarselli *et al.* 2009) and graph masked autoencoders (Hou *et al.* 2022), that have seen applications in scRNA-seq and spatial transcriptomics (Wen *et al.* 2022, Min *et al.* 2025) but not in spatial proteomics.

In our comparisons, for all clustering methods, using the integrated latent space of hmiVAE resulted in clusters with higher associations with clinical variables than clustering on the original feature space. Further, hmiVAE was able to find distinct spatial patterns with poor survival outcomes without any prior knowledge, indicating that this information is contained in IMC data and can be learned with deep learning approaches in an integrated way. This shows the importance of incorporating views beyond per-cell mean protein expression to learn cell states associated with disease. We expect that spatial patterns and novel cell states learned by hmiVAE can be related to transcriptome or genome data where there exist match patient samples. Such a study could reveal whether different genomic programs are present in patients exhibiting higher or lower prevalence of specific cell states or spatial patterns. Further, methods such as DMGN improved survival outcome prediction by using graph neural networks to integrate image features extracted from IMC data with patient variables (Fu *et al.* 2023). Incorporating such approaches with multiple views of IMC data shows promise of further increasing our understanding of patient heterogeneity in disease.

All datasets used in this study were generated using IMC, therefore limiting the generalizability of the results to other highly multiplexed imaging technologies such as CODEX and t-CyCIF (Lin *et al.* 2018, Goltsev *et al.* 2018). However, all these technologies produce a similar set of outputs including pixel-level expression data along with a cell segmentation mask. Therefore, all the views we describe here can be quantified for these technologies. Further, we note that optical imaging-based technologies such as 4i (Gut *et al.* 2018) have much higher resolution than laser ablation-based technologies like IMC (McCarthy and Birtwistle 2019), meaning the quality of some features such as protein nuclear co-localization are likely superior. However, these technologies have additional sources of background noise, such as auto-fluorescence, imperfect overlap between each acquisition run and incomplete antibody deactivation (Giesen *et al.* 2014, Gut *et al.* 2018, Lin *et al.* 2018) which may require further refinement of which technical features to condition on in such models. We also highlight that in our study, we conducted an association analysis between latent features and integrated clusters with clinical variables and patient survival. Further studies could also investigate the predictive performance of these latent features and integrated clusters on clinical variables and patient survival using held-out data which would enable us to gain insights into their prognostic potential.

Our analysis demonstrated that while hmiVAE performed well on integrating the different views of HMI data and a variety of downstream analysis tasks, the applicability of such a method was not without its limitations. The batch correction comparison showed hmiVAE struggles to remove batch effects in a dataset with strong technical differences between images. This could be due to only conditioning on the batch ID which may be insufficient to remove batch effects in such a dataset and there could be room to add methods like Scanorama (Hie *et al.* 2024) into the pipeline to mitigate those effects while maintaining biological information. However, we note that most IMC datasets comprise of images from TMAs and so we only see this issue arising in a dataset where all images come from different slides with vastly different acquisition times. Further, although we show that the clinical associations we find are not impacted by the choice of segmentation algorithm, this does not fully address the impact of segmentation on the features extracted. Quality of cell segmentation is difficult to assess as there are many sources of errors and algorithms often correct for these errors during cell clustering (Lee *et al.* 2025). Also, methods developed to detect segmentation errors using images, such as the ESQmodel (Lee *et al.* 2024), rely on prior knowledge of cell type composition within the dataset, which is often unknown in IMC data. This presents a challenge when correcting for segmentation artefacts during feature extraction. However, given cell segmentation models like Mesmer (Greenwald *et al.* 2022) provide whole-cell versus nuclear masks and employing strategies of comparing these two types of masks, similar to STARLING (Lee *et al.* 2025), segmentation error probabilities could be added as covariates to future models.

The latent projection analysis showed that the learnt expression and spatial context embedding generalized well over datasets with similar panel designs, but this was not the case for the integrated latent space and the nuclear co-localization embedding, underlying the need to investigate batch effects during projection for the different views. It may also be beneficial to understand how network architecture affects this projection of new cells onto the latent space or view-specific embeddings. Further work is also required to determine how well the learnt latent space generalizes over datasets with different panel designs, tissue types, and disease conditions as well as which dataset characteristics may impact generalization.

However, it is to be noted that latent space projection in these settings would require more careful architecture design, paying closer attention to missing proteins and batch correction. Missing protein imputation is an area of active research and is often done in settings where protein data is paired with transcriptomic data such as in the case of totalVI which uses CITE-seq which has paired scRNA-seq and surface protein data (Gayoso *et al.* 2021). Therefore, for such studies with IMC data, additional data modalities would be required and this would be a case of diagonal data integration where an attempt is made to integrate data coming from different cells belonging to different modalities, which is known to be a very challenging task (Argelaguet *et al.* 2021). Further, using a KNN model for projection analysis, especially of the latent space may not be suitable as it relies on distance metrics such as Euclidean distance or cosine similarity to determine closeness of points. These metrics for similarity have been seen to lead to arbitrary results when applied to learned latent space vectors (Steck *et al.* 2024). It is worth noting that finding effective quantitative benchmarks and metrics for deep learning methods using HMI data is challenging since there is a lack of ground truth annotations as it involves time and expertise which might not be readily available (Carstens *et al.* 2024).

Overall, our investigation shows that deep learning methods such as VAEs can successfully integrate different views of HMI data to learn cell state representations associated with clinically relevant variables in cancer. Deep learning methods for HMI data are still in their infancy, and our work highlights their potential to improve understanding of patient-level disease presentation by leveraging rich information captured by HMI technologies.

## Supplementary Material

vbag010_Supplementary_Data

## Data Availability

Code required to reproduce this study can be found at https://github.com/camlab-bioml/hmiVAE_manuscript. The data underlying this article are available in zenodo and can be accessed with https://doi.org/10.5281/zenodo.17048786.
