# Supplementary material for: Multi-view deep learning of highly multiplexed imaging data improves association of cell states with clinical outcomes
Source: Bioinform Adv. 2026 Jan 14;6(1):vbag010. doi: 10.1093/bioadv/vbag010 (PMC12955845; doi:10.1093/bioadv/vbag010)
Supplement: vbag010_Supplementary_Data [file vbag010_supplementary_data.pdf]

Supplementary Materials

Table S1. Description for all datasets used in this study.

Cohort	# of Images	# of cells	Panel design	Clinical variables	Tissue	Segmentation method	# of markers
Jackson-BC	358	802591	Stromal/Epithelial	HR/HER2 status, Clinical subtype, Cancer stage, Treatment response	Breast	CellProfiler + Ilastik	34
Ali-BC	548	536883	Stromal/Epithelial	ER status, Cancer grade and stage	Breast	CellProfiler + Ilastik	37
Hoch-Melanoma	167	989404	Immune	Cancer stage, Mutation, Primary type, Treatment response	Skin	CellProfiler +Ilastik	44
PDAC	5	77661	Immune	N/A	Pancreas, NOS	DeepCell	37

Table S2. Optimal hmiVAE model hyperparameters for each dataset.

Cohort	Initialization (random seed)	# of hidden layers	Hidden layer dim.	Latent representation dim.	Beta scheme	Batch size
Jackson-BC	123	1	64	20	Warm-up	8000
Ali-BC	1234	2	32	20	Warm-up	4000
Hoch-Melanoma	123	1	64	20	Warm-up	16000
Ali-BC-DC	0	1	64	20	Warm-up	4000
PDAC	42	2	8	20	Warm-up	466

Table S3. Markers used for assigning cell type labels to clusters in this study.

Marker	Cell type	Note
SMA / Vimentin / Fibronectin	Stromal / Fibroblasts	"Fibroblasts" if only Fibronectin is highly expressed. If all or multiple expressed, labelled as "stromal"
PanCK / CK8_18 / CK7 / CK19	Luminal epithelial	
E_Cadherin	Epithelial	If more specific markers expressed, such as CK8_18 etc. then those makers are given precedence
CK5 / CK14	Basal epithelial	

vWF / CD31 / Caveolin	Endothelial	
CD45 / CD3	Immune / T cells	For cases where there are multiple immune markers or only CD45, labelled as “immune”<br><br>For Hoch-Melanoma, there are further activation markers present which are included as well (**Methods**)(Hoch et al. 2022)
CD45 / CD20	Immune / B cells	For cases where there are multiple immune markers or only CD45, labelled as “immune”<br><br>For Hoch-Melanoma, there are further activation markers present which are included as well (**Methods**)(Hoch et al. 2022)
CD45 / CD68 / Vimentin	Immune / Macrophages	These are difficult to label as macrophages could also be “expressing” different markers due to their role as phagocytes.<br><br>For cases where there are multiple immune markers or only CD45, labelled as “immune”
CD303 / MPO / MMR<br><br>(Hoch-Melanoma only)	Myeloid / Dendritic cells	
Multiple	Mix	Multiple lineage markers expressed

None	None / Unknown	No lineage markers seem to be highly expressed
Other		If some marker is highly expressed but no lineage markers are expressed, then they are given general labels e.g. cluster showing high expression of Slug is labelled “Slug+ cells”

Table S4. Metal and marker panel used in generating the PDAC dataset including vendor and antibody details.

Metal	Target	Antibody clone	Vendor	Catalogue #	Lot #
Y89	SMA & Vimentin	1A4 and EPR3776	Thermo and Abcam	14-9760-82 and ab193555	2288516 and GR3396611
Pr141	HLA-DR	TAL 1B5	Abcam	ab176408	GR3384096-1
Nd142	CD303	Polyclonal	R&D Systems	AF1376	IDL0218101
Nd143	PD-L1	73-10	Abcam	ab226766	GR3337035-18
Nd144	CD28	EPR22076	Abcam	ab243557	GR3358395-2
Nd145	CD15	HI98	Bio-Rad	MCA4610T	156536
Nd146	Cytokeratin 19	Troma III	Sigma	MABT913	3379915
Sm147	CD66b	G10F5	Novus Biologicals	NB100-77808	A-8

Nd148	ICOS	D1K2T	Cell Signaling Technology	89601BF	9
Sm149	CD20	L26	Thermo	14-0202-82	2172592
Nd150	CD68	KP1	Thermo	14-0688-82	2265228
Eu151	CD4	EPR6855	Abcam	ab181724	GR3352909-4
Sm152	CD8a	C8/144B	Thermo	14-0085-82	2247491
Eu153	CD127	EPR23747-333	Abcam	ab282011	GR3389947-1
Sm154	CD11c	EP1347Y	Abcam	ab216655	GR3357092-4
Gd155	CD141	EPR4051	Abcam	ab271880	GR3370279-2
Gd156	FOXP3	221D	Abcam	ab255942	GR3351299-1
Gd158	GATA3	L50-823	BD Biosciences	558686	9346413
Tb159	Tbet	E4I2K	Cell Signaling Technology	97135BF	2
Gd160	Anti-Rabbit for CD45	Polyclonal	Thermo	A27033	RL246119A
Dy161	Perforin	B-D48	Abcam	ab47225	GR3333562-7
Dy162	CD45RO	UCHL1	Cell Signaling Technology	55618BF	2

Dy163	Anti-Rat for CD3	Polyclonal	Thermo	A18873	61-172-060320
Dy164	Granzyme B	D6E9W	Cell Signaling Technology	46890BF	4
Ho165	CTLA-4	CAL49	Abcam	ab251599	GR3344009-8
Er166	CD73	D7F9A	Cell Signaling Technology	13160	2
Er167	iNOS	SP126	Abcam	ab239990	GR3288157-2
Er168	Arginase	EPR6672(B)	Abcam	ab211961	GR3231162-14
Tm169	HLA-ABC	EMR8-5	BD Biosciences	565292	1117370
Er170	CD31	RM1006	Abcam	ab282746	GR3399060-2
Yb171	CD39	EPR20461	Abcam	ab232537	GR3281156-3
Yb172	CCR7	EPR23192-57	Abcam	ab272938	GR3391275-2
Yb173	CD56	EPR2566	Abcam	ab214436	GR3317476-5
Yb174	S100A9	MAC387	Thermo	MA1-81381	VJ3101852
Lu175	CD1c	3G1B3	Novus Biologicals	NBP2-61725	170831
Yb176	PD-1	D4W2J	Cell Signaling Technology	86163BF	7

Pt194	CD45	D9M8I	Cell Signaling Technology	13917BF	11
Pt195	PNAd	MECA-79	BioLegend	120802	B305020
Unconjugated	CD3	CD3-12	Thermo	MA5-16622	WG3333396

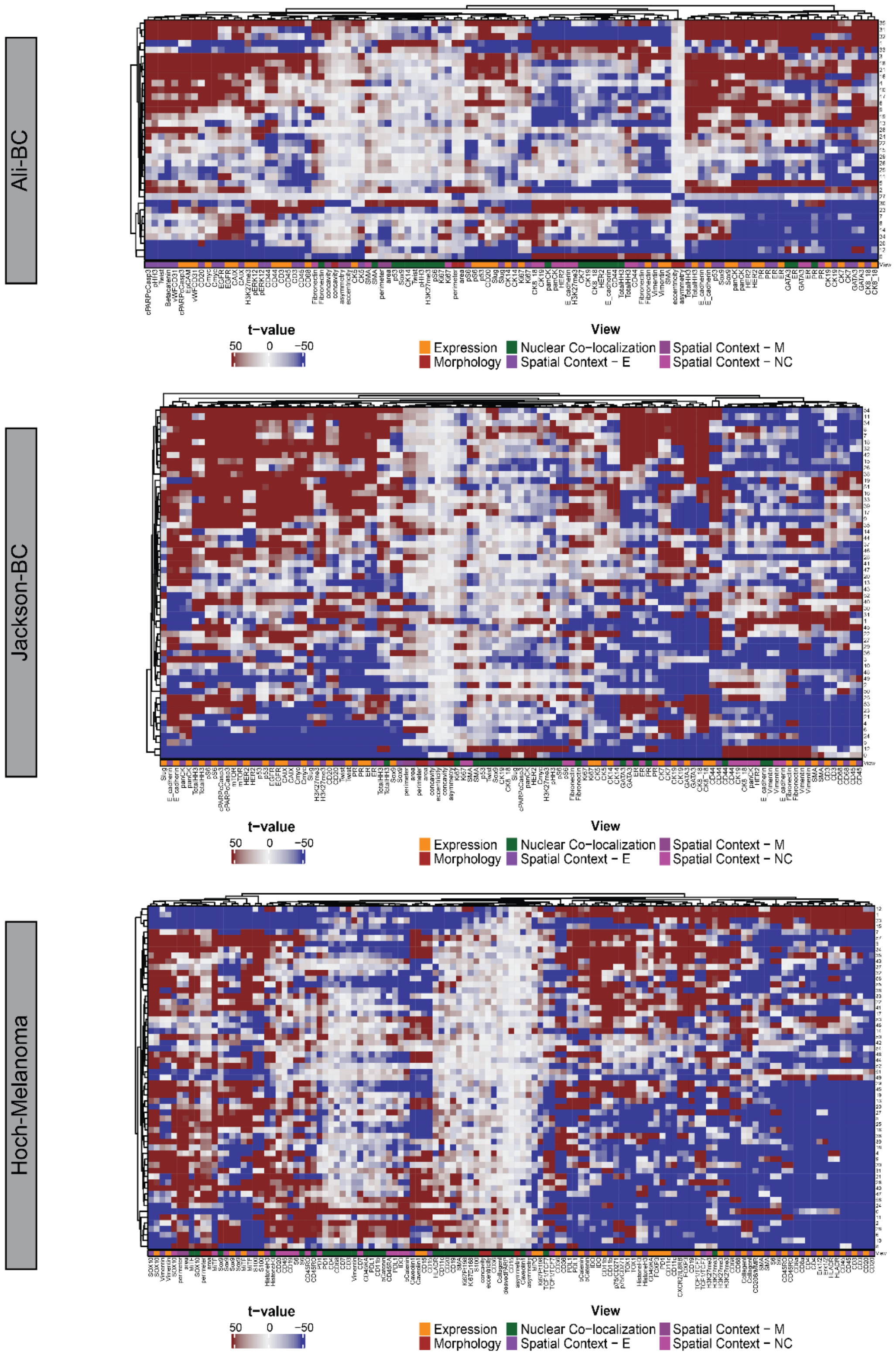

Supplementary Figure 1. Ranking of all features for Leiden across datasets.

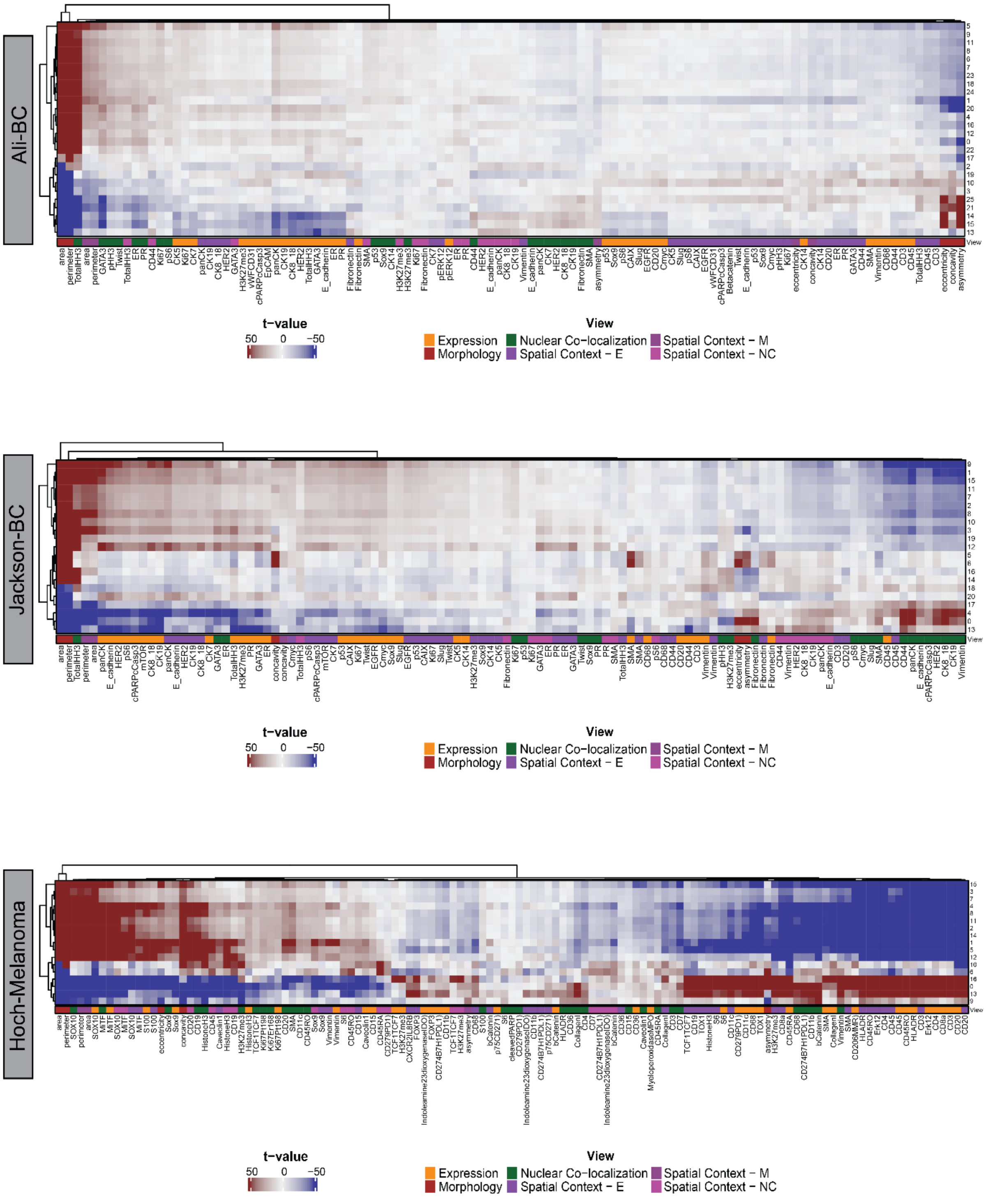

Supplementary Figure 2. Ranking of all features for FlowSOM across datasets.

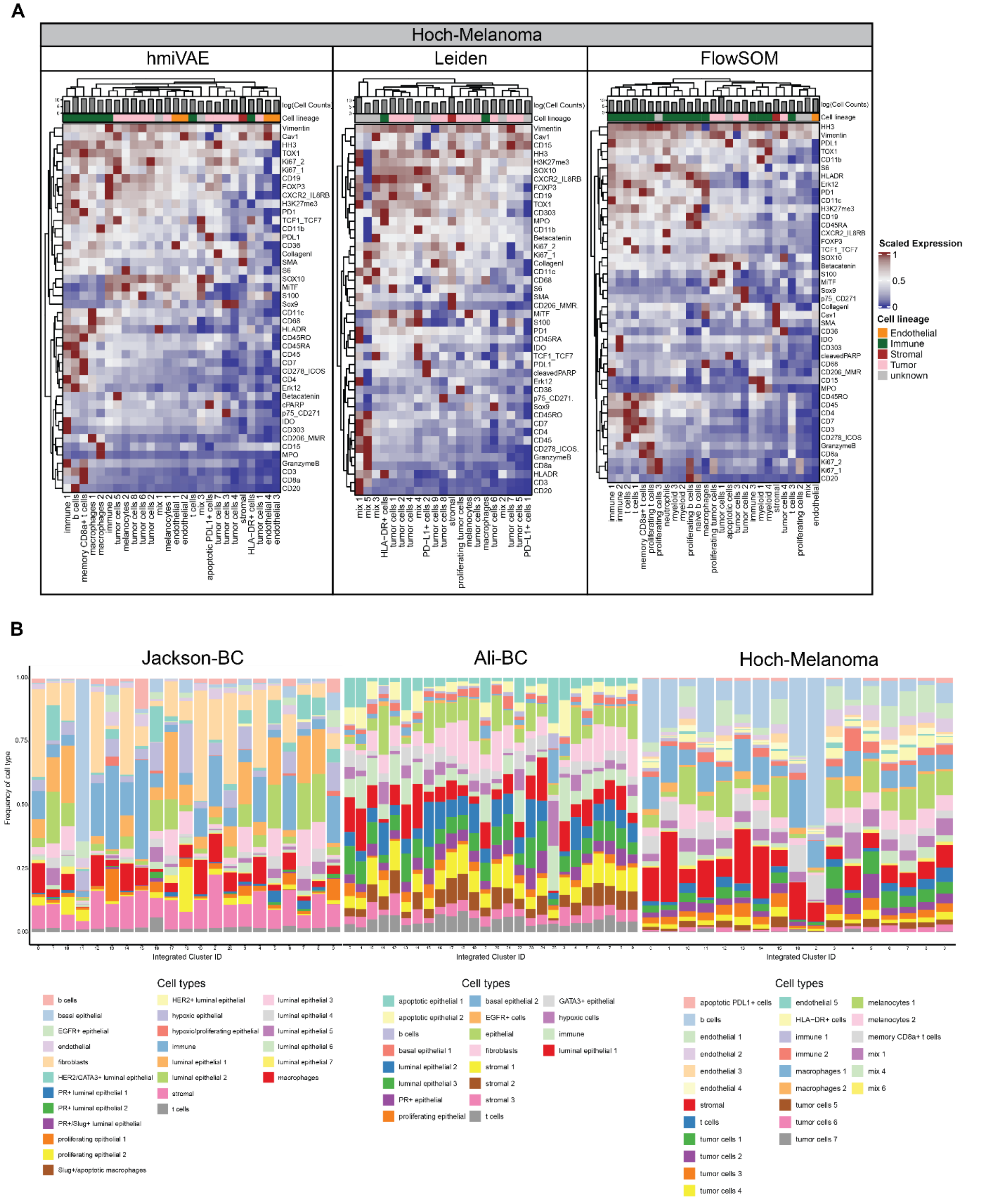

Supplementary Figure 3. A Cell types identified using hmiVAE vs identified by Leiden and FlowSOM in Hoch-Melanoma. **B** Proportion of cell types occurring in each integrated cluster from hmiVAE.

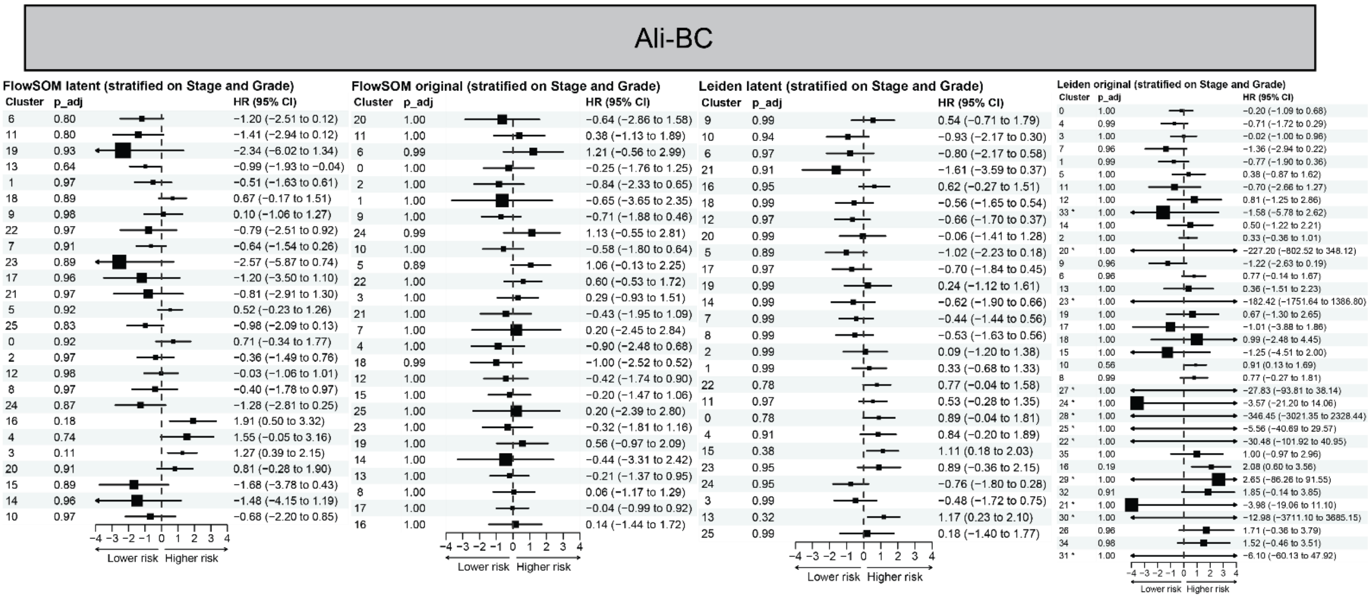
Ali-BC
FlowSOM latent (stratified on Stage and Grade)
Cluster p_adj HR (95% CI)
6 0.80 −1.20 (−2.51 to 0.12)
11 0.80 −1.41 (−2.94 to 0.12)
19 0.93 −2.34 (−6.02 to 1.34)
13 0.64 −0.99 (−1.93 to −0.04)
1 0.97 −0.51 (−1.63 to 0.61)
18 0.89 0.67 (−0.17 to 1.51)
9 0.98 0.10 (−1.06 to 1.27)
22 0.97 −0.79 (−2.51 to 0.92)
7 0.91 −0.64 (−1.54 to 0.26)
23 0.89 −2.57 (−5.87 to 0.74)
17 0.96 −1.20 (−3.50 to 1.10)
21 0.97 −0.81 (−2.91 to 1.30)
5 0.92 0.52 (−0.23 to 1.26)
25 0.83 −0.98 (−2.09 to 0.13)
0 0.92 0.71 (−0.34 to 1.77)
2 0.97 −0.36 (−1.49 to 0.76)
12 0.98 −0.03 (−1.06 to 1.01)
8 0.97 −0.40 (−1.78 to 0.97)
24 0.87 −1.28 (−2.81 to 0.25)
16 0.18 1.91 (0.50 to 3.32)
4 0.74 1.55 (−0.05 to 3.16)
3 0.11 1.27 (0.39 to 2.15)
20 0.91 0.81 (−0.28 to 1.90)
15 0.89 −1.68 (−3.78 to 0.43)
14 0.96 −1.48 (−4.15 to 1.19)
10 0.97 −0.68 (−2.20 to 0.85)
−4 −3 −2 −1 0 1 2 3 4
Lower risk Higher risk
FlowSOM original (stratified on Stage and Grade)
Cluster p_adj HR (95% CI)
20 1.00 −0.64 (−2.86 to 1.58)
11 1.00 0.38 (−1.13 to 1.89)
6 0.99 1.21 (−0.56 to 2.99)
0 1.00 −0.25 (−1.76 to 1.25)
2 1.00 −0.84 (−2.33 to 0.65)
1 1.00 −0.65 (−3.65 to 2.35)
9 1.00 −0.71 (−1.88 to 0.46)
24 0.99 1.13 (−0.55 to 2.81)
10 1.00 −0.58 (−1.80 to 0.64)
5 0.89 1.06 (−0.13 to 2.25)
22 1.00 0.60 (−0.53 to 1.72)
3 1.00 0.29 (−0.93 to 1.51)
21 1.00 −0.43 (−1.95 to 1.09)
7 1.00 0.20 (−2.45 to 2.84)
4 1.00 −0.90 (−2.48 to 0.68)
18 0.99 −1.00 (−2.52 to 0.52)
12 1.00 −0.42 (−1.74 to 0.90)
15 1.00 −0.20 (−1.47 to 1.06)
25 1.00 0.20 (−2.39 to 2.80)
23 1.00 −0.32 (−1.81 to 1.16)
19 1.00 0.56 (−0.97 to 2.09)
14 1.00 −0.44 (−3.31 to 2.42)
13 1.00 −0.21 (−1.37 to 0.95)
8 1.00 0.06 (−1.17 to 1.29)
17 1.00 −0.04 (−0.99 to 0.92)
16 1.00 0.14 (−1.44 to 1.72)
−4 −3 −2 −1 0 1 2 3 4
Lower risk Higher risk
Leiden latent (stratified on Stage and Grade)
Cluster p_adj HR (95% CI)
9 0.99 0.54 (−0.71 to 1.79)
10 0.94 −0.93 (−2.17 to 0.30)
6 0.97 −0.80 (−2.17 to 0.58)
21 0.91 −1.61 (−3.59 to 0.37)
16 0.95 0.62 (−0.27 to 1.51)
18 0.99 −0.56 (−1.65 to 0.54)
12 0.97 −0.66 (−1.70 to 0.37)
20 0.99 −0.06 (−1.41 to 1.28)
5 0.89 −1.02 (−2.23 to 0.18)
17 0.97 −0.70 (−1.84 to 0.45)
19 0.99 0.24 (−1.12 to 1.61)
14 0.99 −0.62 (−1.90 to 0.66)
7 0.99 −0.44 (−1.44 to 0.56)
8 0.99 −0.53 (−1.63 to 0.56)
2 0.99 0.09 (−1.20 to 1.38)
1 0.99 0.33 (−0.68 to 1.33)
22 0.78 0.77 (−0.04 to 1.58)
11 0.97 0.53 (−0.28 to 1.35)
0 0.78 0.89 (−0.04 to 1.81)
4 0.91 0.84 (−0.20 to 1.89)
15 0.38 1.11 (0.18 to 2.03)
23 0.95 0.89 (−0.36 to 2.15)
24 0.95 −0.76 (−1.80 to 0.28)
3 0.99 −0.48 (−1.72 to 0.75)
13 0.32 1.17 (0.23 to 2.10)
25 0.99 0.18 (−1.40 to 1.77)
−4 −3 −2 −1 0 1 2 3 4
Lower risk Higher risk
Leiden original (stratified on Stage and Grade)
Cluster p_adj HR (95% CI)
0 1.00 −0.20 (−1.09 to 0.68)
4 0.99 −0.71 (−1.72 to 0.29)
3 1.00 −0.02 (−1.00 to 0.96)
7 0.96 −1.36 (−2.94 to 0.22)
1 0.99 −0.77 (−1.90 to 0.36)
5 1.00 0.38 (−0.87 to 1.62)
11 1.00 −0.70 (−2.66 to 1.27)
12 1.00 0.81 (−1.25 to 2.86)
33 * 1.00 −1.58 (−5.78 to 2.62)
14 1.00 0.50 (−1.22 to 2.21)
2 1.00 0.33 (−0.36 to 1.01)
20 * 1.00 −227.20 (−802.52 to 348.12)
9 0.96 −1.22 (−2.63 to 0.19)
6 0.96 0.77 (−0.14 to 1.67)
13 1.00 0.36 (−1.51 to 2.23)
23 * 1.00 −182.42 (−1751.64 to 1386.80)
19 1.00 0.67 (−1.30 to 2.65)
17 1.00 −1.01 (−3.88 to 1.86)
18 1.00 0.99 (−2.48 to 4.45)
15 1.00 −1.25 (−4.51 to 2.00)
10 0.56 0.91 (0.13 to 1.69)
8 0.99 0.77 (−0.27 to 1.81)
27 * 1.00 −27.83 (−93.81 to 38.14)
24 * 1.00 −3.57 (−21.20 to 14.06)
28 * 1.00 −346.45 (−3021.35 to 2328.44)
25 * 1.00 −5.56 (−40.69 to 29.57)
22 * 1.00 −30.48 (−101.92 to 40.95)
35 1.00 1.00 (−0.97 to 2.96)
16 0.19 2.08 (0.60 to 3.56)
29 * 1.00 2.65 (−86.26 to 91.55)
32 0.91 1.85 (−0.14 to 3.85)
21 * 1.00 −3.98 (−19.06 to 11.10)
30 * 1.00 −12.98 (−3711.10 to 3685.15)
26 0.96 1.71 (−0.36 to 3.79)
34 0.98 1.52 (−0.46 to 3.51)
31 * 1.00 −6.10 (−60.13 to 47.92)
−4 −3 −2 −1 0 1 2 3 4
Lower risk Higher risk

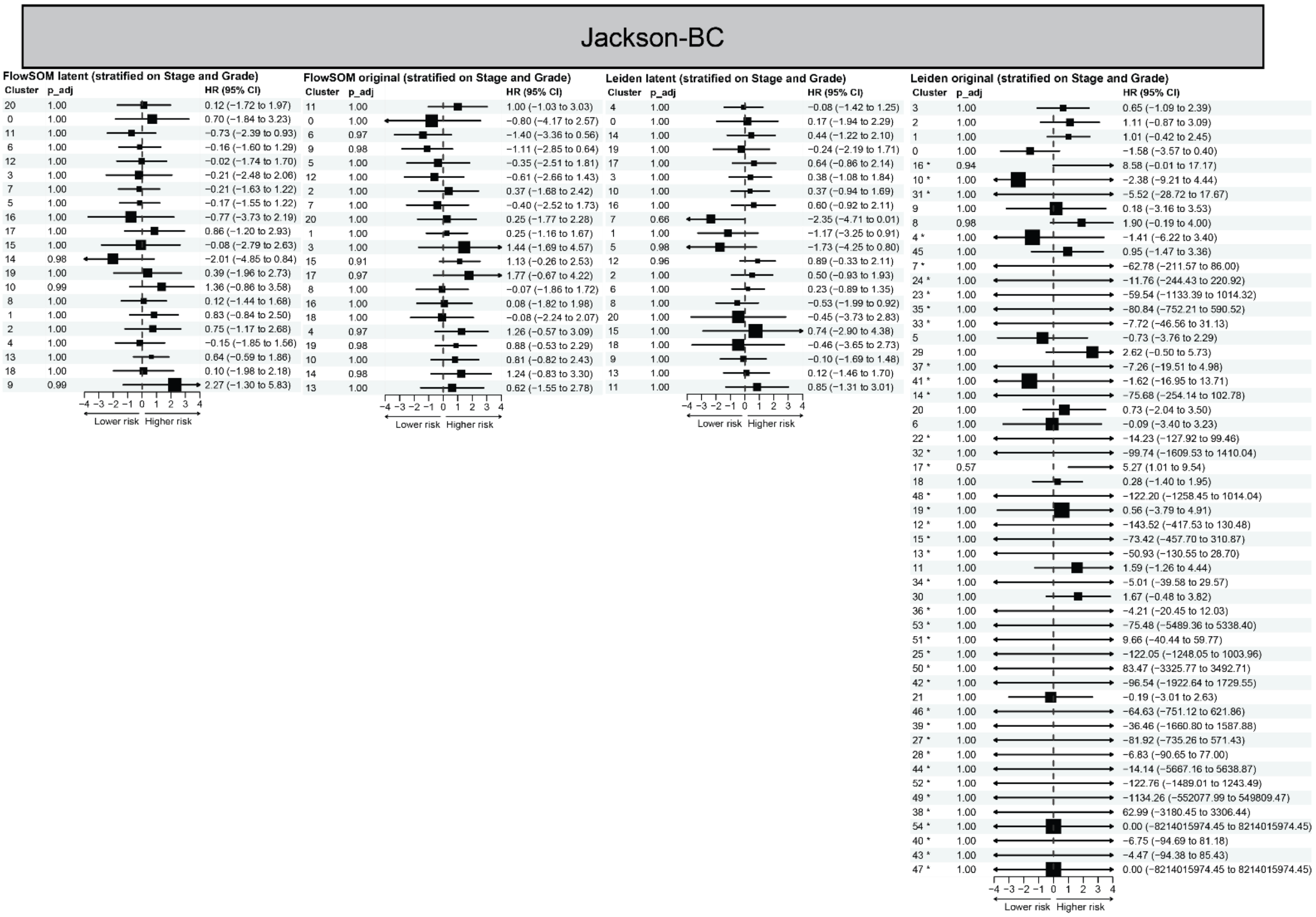
Jackson-BC
FlowSOM latent (stratified on Stage and Grade)
Cluster p_adj HR (95% CI)
20 1.00 0.12 (−1.72 to 1.97)
0 1.00 0.70 (−1.84 to 3.23)
11 1.00 −0.73 (−2.39 to 0.93)
6 1.00 −0.16 (−1.60 to 1.29)
12 1.00 −0.02 (−1.74 to 1.70)
3 1.00 −0.21 (−2.48 to 2.06)
7 1.00 −0.21 (−1.63 to 1.22)
5 1.00 −0.17 (−1.55 to 1.22)
16 1.00 −0.77 (−3.73 to 2.19)
17 1.00 0.86 (−1.20 to 2.93)
15 1.00 −0.08 (−2.79 to 2.63)
14 0.98 −2.01 (−4.85 to 0.84)
19 1.00 0.39 (−1.96 to 2.73)
10 0.99 1.36 (−0.86 to 3.58)
8 1.00 0.12 (−1.44 to 1.68)
1 1.00 0.83 (−0.84 to 2.50)
2 1.00 0.75 (−1.17 to 2.68)
4 1.00 −0.15 (−1.85 to 1.56)
13 1.00 0.64 (−0.59 to 1.86)
18 1.00 0.10 (−1.98 to 2.18)
9 0.99 2.27 (−1.30 to 5.83)
−4 −3 −2 −1 0 1 2 3 4
Lower risk Higher risk
FlowSOM original (stratified on Stage and Grade)
Cluster p_adj HR (95% CI)
11 1.00 1.00 (−1.03 to 3.03)
0 1.00 −0.80 (−4.17 to 2.57)
6 0.97 −1.40 (−3.36 to 0.56)
9 0.98 −1.11 (−2.85 to 0.64)
5 1.00 −0.35 (−2.51 to 1.81)
12 1.00 −0.61 (−2.66 to 1.43)
2 1.00 0.37 (−1.68 to 2.42)
7 1.00 −0.40 (−2.52 to 1.73)
20 1.00 0.25 (−1.77 to 2.28)
1 1.00 0.25 (−1.16 to 1.67)
3 1.00 1.44 (−1.69 to 4.57)
15 0.91 1.13 (−0.26 to 2.53)
17 0.97 1.77 (−0.67 to 4.22)
8 1.00 −0.07 (−1.86 to 1.72)
16 1.00 0.08 (−1.82 to 1.98)
18 1.00 −0.08 (−2.24 to 2.07)
4 0.97 1.26 (−0.57 to 3.09)
19 0.98 0.88 (−0.53 to 2.29)
10 1.00 0.81 (−0.82 to 2.43)
14 0.98 1.24 (−0.83 to 3.30)
13 1.00 0.62 (−1.55 to 2.78)
−4 −3 −2 −1 0 1 2 3 4
Lower risk Higher risk
Leiden latent (stratified on Stage and Grade)
Cluster p_adj HR (95% CI)
4 1.00 −0.08 (−1.42 to 1.25)
0 1.00 0.17 (−1.94 to 2.29)
14 1.00 0.44 (−1.22 to 2.10)
19 1.00 −0.24 (−2.19 to 1.71)
17 1.00 0.64 (−0.86 to 2.14)
3 1.00 0.38 (−1.08 to 1.84)
10 1.00 0.37 (−0.94 to 1.69)
16 1.00 0.60 (−0.92 to 2.11)
7 0.66 −2.35 (−4.71 to 0.01)
1 1.00 −1.17 (−3.25 to 0.91)
5 0.98 −1.73 (−4.25 to 0.80)
12 0.96 0.89 (−0.33 to 2.11)
2 1.00 0.50 (−0.93 to 1.93)
6 1.00 0.23 (−0.89 to 1.35)
8 1.00 −0.53 (−1.99 to 0.92)
20 1.00 −0.45 (−3.73 to 2.83)
15 1.00 0.74 (−2.90 to 4.38)
18 1.00 −0.46 (−3.65 to 2.73)
9 1.00 −0.10 (−1.69 to 1.48)
13 1.00 0.12 (−1.46 to 1.70)
11 1.00 0.85 (−1.31 to 3.01)
−4 −3 −2 −1 0 1 2 3 4
Lower risk Higher risk
Leiden original (stratified on Stage and Grade)
Cluster p_adj HR (95% CI)
3 1.00 0.65 (−1.09 to 2.39)
2 1.00 1.11 (−0.87 to 3.09)
1 1.00 1.01 (−0.42 to 2.45)
0 1.00 −1.58 (−3.57 to 0.40)
16 * 0.94 8.58 (−0.01 to 17.17)
10 * 1.00 −2.38 (−9.21 to 4.44)
31 * 1.00 −5.52 (−28.72 to 17.67)
9 1.00 0.18 (−3.16 to 3.53)
8 0.98 1.90 (−0.19 to 4.00)
4 * 1.00 −1.41 (−6.22 to 3.40)
45 1.00 0.95 (−1.47 to 3.36)
7 * 1.00 −62.78 (−211.57 to 86.00)
24 * 1.00 −11.76 (−244.43 to 220.92)
23 * 1.00 −59.54 (−1133.39 to 1014.32)
35 * 1.00 −80.84 (−752.21 to 590.52)
33 * 1.00 −7.72 (−46.56 to 31.13)
5 1.00 −0.73 (−3.76 to 2.29)
29 1.00 2.62 (−0.50 to 5.73)
37 * 1.00 −7.26 (−19.51 to 4.98)
41 * 1.00 −1.62 (−16.95 to 13.71)
14 * 1.00 −75.68 (−254.14 to 102.78)
20 1.00 0.73 (−2.04 to 3.50)
6 1.00 −0.09 (−3.40 to 3.23)
22 * 1.00 −14.23 (−127.92 to 99.46)
32 * 1.00 −99.74 (−1609.53 to 1410.04)
17 * 0.57 5.27 (1.01 to 9.54)
18 1.00 0.28 (−1.40 to 1.95)
48 * 1.00 −122.20 (−1258.45 to 1014.04)
19 * 1.00 0.56 (−3.79 to 4.91)
12 * 1.00 −143.52 (−417.53 to 130.48)
15 * 1.00 −73.42 (−457.70 to 310.87)
13 * 1.00 −50.93 (−130.55 to 28.70)
11 1.00 1.59 (−1.26 to 4.44)
34 * 1.00 −5.01 (−39.58 to 29.57)
30 1.00 1.67 (−0.48 to 3.82)
36 * 1.00 −4.21 (−20.45 to 12.03)
53 * 1.00 −75.48 (−5489.36 to 5338.40)
51 * 1.00 9.66 (−40.44 to 59.77)
25 * 1.00 −122.05 (−1248.05 to 1003.96)
50 * 1.00 83.47 (−3325.77 to 3492.71)
42 * 1.00 −96.54 (−1922.64 to 1729.55)
21 1.00 −0.19 (−3.01 to 2.63)
46 * 1.00 −64.63 (−751.12 to 621.86)
39 * 1.00 −36.46 (−1660.80 to 1587.88)
27 * 1.00 −81.92 (−735.26 to 571.43)
28 * 1.00 −6.83 (−90.65 to 77.00)
44 * 1.00 −14.14 (−5667.16 to 5638.87)
52 * 1.00 −122.76 (−1489.01 to 1243.49)
49 * 1.00 −1134.26 (−552077.99 to 549809.47)
38 * 1.00 62.99 (−3180.45 to 3306.44)
54 * 1.00 0.00 (−8214015974.45 to 8214015974.45)
40 * 1.00 −6.75 (−94.69 to 81.18)
43 * 1.00 −4.47 (−94.38 to 85.43)
47 * 1.00 0.00 (−8214015974.45 to 8214015974.45)
−4 −3 −2 −1 0 1 2 3 4
Lower risk Higher risk

Supplementary Figure 4. CoxPH model hazard ratios with cluster proportions for clusters from FlowSOM and Leiden using original and latent spaces for Ali-BC and Jackson-BC datasets. P-values are multiple tests corrected using Benjamini-Hochberg correction. *: Circle size capped due to high standard error (se.coef > 2).

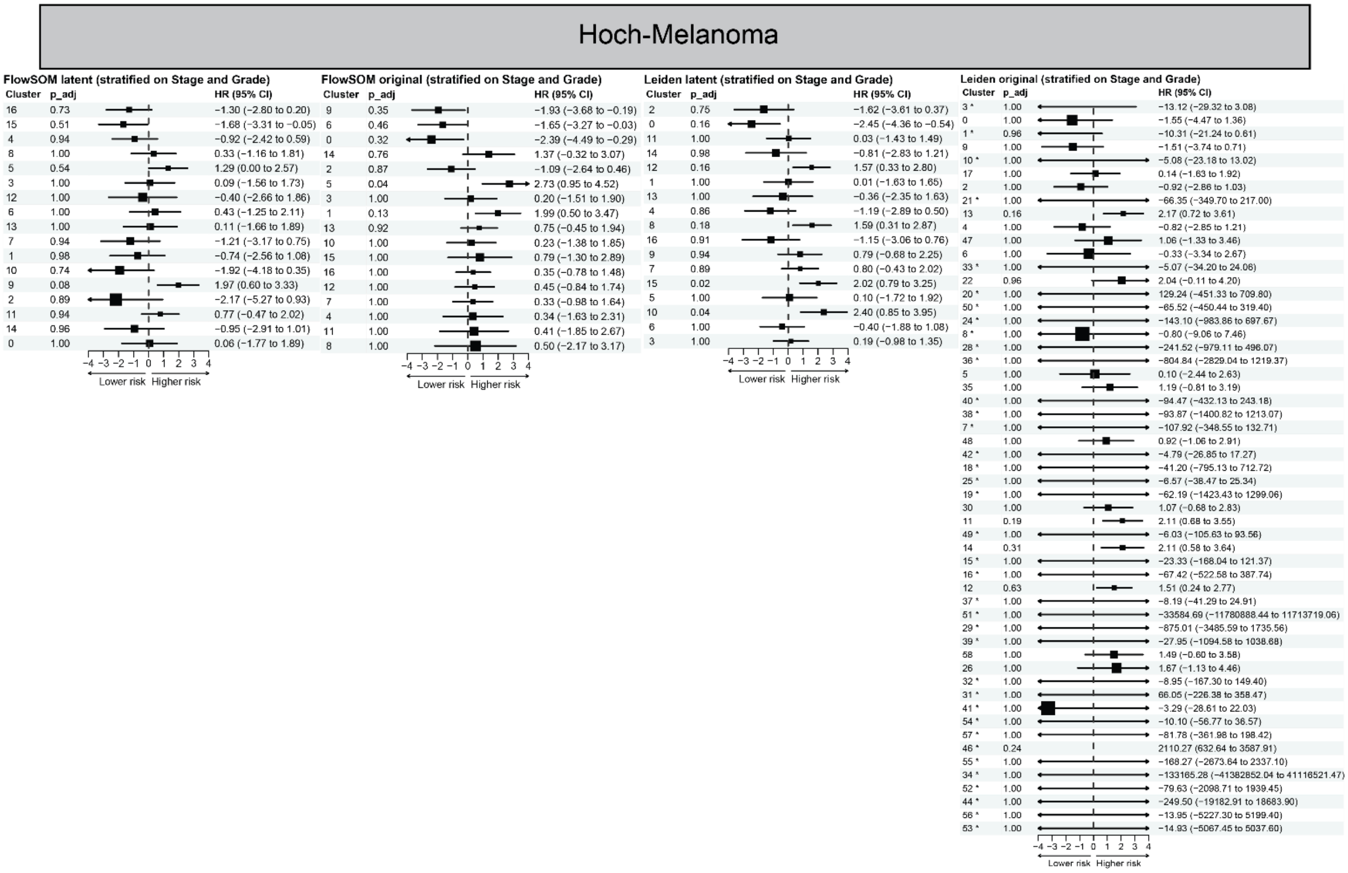

Supplementary Figure 5. CoxPH model hazard ratios with cluster proportions for clusters from FlowSOM and Leiden using original and latent spaces for Hoch-Melanoma dataset. P-values are multiple tests corrected using Benjamini-Hochberg correction. *: Circle size capped due to high standard error (se.coef > 2).

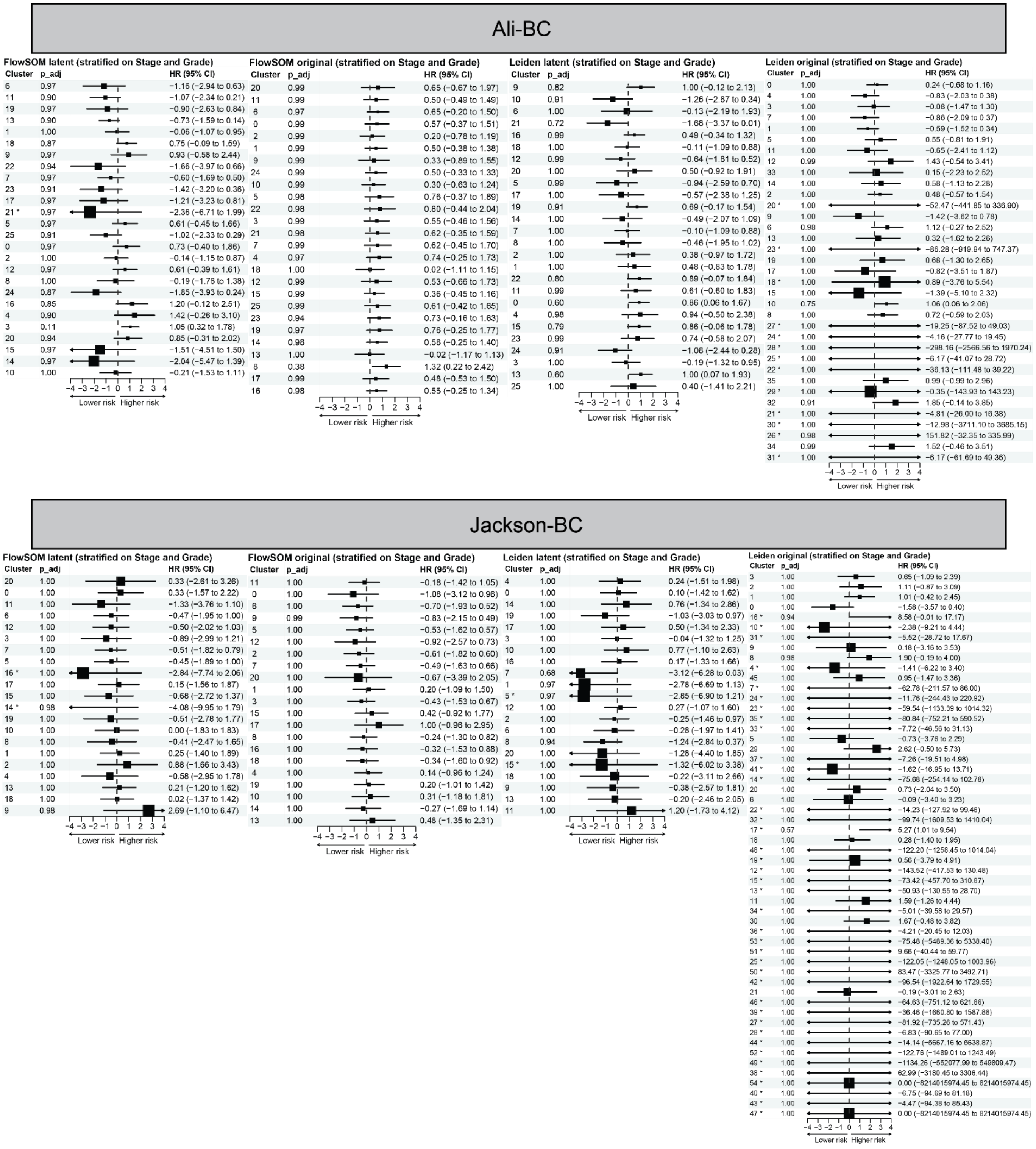
Ali-BC
FlowSOM latent (stratified on Stage and Grade)
Cluster p_adj HR (95% CI)
6 0.97 −1.16 (−2.94 to 0.63)
11 0.90 −1.07 (−2.34 to 0.21)
19 0.97 −0.90 (−2.63 to 0.84)
13 0.90 −0.73 (−1.59 to 0.14)
1 1.00 −0.06 (−1.07 to 0.95)
18 0.87 0.75 (−0.09 to 1.59)
9 0.97 0.93 (−0.58 to 2.44)
22 0.94 −1.66 (−3.97 to 0.66)
7 0.97 −0.60 (−1.69 to 0.50)
23 0.91 −1.42 (−3.20 to 0.36)
17 0.97 −1.21 (−3.23 to 0.81)
21 * 0.97 −2.36 (−6.71 to 1.99)
5 0.97 0.61 (−0.45 to 1.66)
25 0.91 −1.02 (−2.33 to 0.29)
0 0.97 0.73 (−0.40 to 1.86)
2 1.00 −0.14 (−1.15 to 0.87)
12 0.97 0.61 (−0.39 to 1.61)
8 1.00 −0.19 (−1.76 to 1.38)
24 0.87 −1.85 (−3.93 to 0.24)
16 0.85 1.20 (−0.12 to 2.51)
4 0.90 1.42 (−0.26 to 3.10)
3 0.11 1.05 (0.32 to 1.78)
20 0.94 0.85 (−0.31 to 2.02)
15 0.97 −1.51 (−4.51 to 1.50)
14 0.97 −2.04 (−5.47 to 1.39)
10 1.00 −0.21 (−1.53 to 1.11)
−4 −3 −2 −1 0 1 2 3 4
Lower risk Higher risk
FlowSOM original (stratified on Stage and Grade)
Cluster p_adj HR (95% CI)
20 0.99 0.65 (−0.67 to 1.97)
11 0.99 0.50 (−0.49 to 1.49)
6 0.97 0.65 (−0.20 to 1.50)
0 0.99 0.57 (−0.37 to 1.51)
2 0.99 0.20 (−0.78 to 1.19)
1 0.99 0.50 (−0.38 to 1.38)
9 0.99 0.33 (−0.89 to 1.55)
24 0.99 0.50 (−0.33 to 1.33)
10 0.99 0.30 (−0.63 to 1.24)
5 0.98 0.76 (−0.37 to 1.89)
22 0.98 0.80 (−0.44 to 2.04)
3 0.99 0.55 (−0.46 to 1.56)
21 0.98 0.62 (−0.35 to 1.59)
7 0.99 0.62 (−0.45 to 1.70)
4 0.97 0.74 (−0.25 to 1.73)
18 1.00 0.02 (−1.11 to 1.15)
12 0.99 0.53 (−0.66 to 1.73)
15 0.99 0.36 (−0.45 to 1.16)
25 0.99 0.61 (−0.42 to 1.65)
23 0.94 0.73 (−0.16 to 1.63)
19 0.97 0.76 (−0.25 to 1.77)
14 0.98 0.58 (−0.25 to 1.40)
13 1.00 −0.02 (−1.17 to 1.13)
8 0.38 1.32 (0.22 to 2.42)
17 0.99 0.48 (−0.53 to 1.50)
16 0.98 0.55 (−0.25 to 1.34)
−4 −3 −2 −1 0 1 2 3 4
Lower risk Higher risk
Leiden latent (stratified on Stage and Grade)
Cluster p_adj HR (95% CI)
9 0.82 1.00 (−0.12 to 2.13)
10 0.91 −1.26 (−2.87 to 0.34)
6 1.00 −0.13 (−2.19 to 1.93)
21 0.72 −1.68 (−3.37 to 0.01)
16 0.99 0.49 (−0.34 to 1.32)
18 1.00 −0.11 (−1.09 to 0.88)
12 0.99 −0.64 (−1.81 to 0.52)
20 1.00 0.50 (−0.92 to 1.91)
5 0.99 −0.94 (−2.59 to 0.70)
17 1.00 −0.57 (−2.38 to 1.25)
19 0.91 0.69 (−0.17 to 1.54)
14 1.00 −0.49 (−2.07 to 1.09)
7 1.00 −0.10 (−1.09 to 0.88)
8 1.00 −0.46 (−1.95 to 1.02)
2 1.00 0.38 (−0.97 to 1.72)
1 1.00 0.48 (−0.83 to 1.78)
22 0.80 0.89 (−0.07 to 1.84)
11 0.99 0.61 (−0.60 to 1.83)
0 0.60 0.86 (0.06 to 1.67)
4 0.98 0.94 (−0.50 to 2.38)
15 0.79 0.86 (−0.06 to 1.78)
23 0.99 0.74 (−0.58 to 2.07)
24 0.91 −1.08 (−2.44 to 0.28)
3 1.00 −0.19 (−1.32 to 0.95)
13 0.60 1.00 (0.07 to 1.93)
25 1.00 0.40 (−1.41 to 2.21)
−4 −3 −2 −1 0 1 2 3 4
Lower risk Higher risk
Leiden original (stratified on Stage and Grade)
Cluster p_adj HR (95% CI)
0 1.00 0.24 (−0.68 to 1.16)
4 1.00 −0.83 (−2.03 to 0.38)
3 1.00 −0.08 (−1.47 to 1.30)
7 1.00 −0.86 (−2.09 to 0.37)
1 1.00 −0.59 (−1.52 to 0.34)
5 1.00 0.55 (−0.81 to 1.91)
11 1.00 −0.65 (−2.41 to 1.12)
12 0.99 1.43 (−0.54 to 3.41)
33 1.00 0.15 (−2.23 to 2.52)
14 1.00 0.58 (−1.13 to 2.28)
2 1.00 0.48 (−0.57 to 1.54)
20 * 1.00 −52.47 (−441.85 to 336.90)
9 1.00 −1.42 (−3.62 to 0.78)
6 0.98 1.12 (−0.27 to 2.52)
13 1.00 0.32 (−1.62 to 2.26)
23 * 1.00 −86.28 (−919.94 to 747.37)
19 1.00 0.68 (−1.30 to 2.65)
17 1.00 −0.82 (−3.51 to 1.87)
18 * 1.00 0.89 (−3.76 to 5.54)
15 1.00 −1.39 (−5.10 to 2.32)
10 0.75 1.06 (0.06 to 2.06)
8 1.00 0.72 (−0.59 to 2.03)
27 * 1.00 −19.25 (−87.52 to 49.03)
24 * 1.00 −4.16 (−27.77 to 19.45)
28 * 1.00 −298.16 (−2566.56 to 1970.24)
25 * 1.00 −6.17 (−41.07 to 28.72)
22 * 1.00 −36.13 (−111.48 to 39.22)
35 1.00 0.99 (−0.99 to 2.96)
29 * 1.00 −0.35 (−143.93 to 143.23)
32 0.91 1.85 (−0.14 to 3.85)
21 * 1.00 −4.81 (−26.00 to 16.38)
30 * 1.00 −12.98 (−3711.10 to 3685.15)
26 * 0.98 151.82 (−32.35 to 335.99)
34 0.99 1.52 (−0.46 to 3.51)
31 * 1.00 −6.17 (−61.69 to 49.36)
−4 −3 −2 −1 0 1 2 3 4
Lower risk Higher risk
Jackson-BC
FlowSOM latent (stratified on Stage and Grade)
Cluster p_adj HR (95% CI)
20 1.00 0.33 (−2.61 to 3.26)
0 1.00 0.33 (−1.57 to 2.22)
11 1.00 −1.33 (−3.76 to 1.10)
6 1.00 −0.47 (−1.95 to 1.00)
12 1.00 −0.50 (−2.02 to 1.03)
3 1.00 −0.89 (−2.99 to 1.21)
7 1.00 −0.51 (−1.82 to 0.79)
5 1.00 −0.45 (−1.89 to 1.00)
16 * 1.00 −2.84 (−7.74 to 2.06)
17 1.00 0.15 (−1.56 to 1.87)
15 1.00 −0.68 (−2.72 to 1.37)
14 * 0.98 −4.08 (−9.95 to 1.79)
19 1.00 −0.51 (−2.78 to 1.77)
10 1.00 0.00 (−1.83 to 1.83)
8 1.00 −0.41 (−2.47 to 1.65)
1 1.00 0.25 (−1.40 to 1.89)
2 1.00 0.88 (−1.66 to 3.43)
4 1.00 −0.58 (−2.95 to 1.78)
13 1.00 0.21 (−1.20 to 1.62)
18 1.00 0.02 (−1.37 to 1.42)
9 0.98 2.69 (−1.10 to 6.47)
−4 −3 −2 −1 0 1 2 3 4
Lower risk Higher risk
FlowSOM original (stratified on Stage and Grade)
Cluster p_adj HR (95% CI)
11 1.00 −0.18 (−1.42 to 1.05)
0 1.00 −1.08 (−3.12 to 0.96)
6 1.00 −0.70 (−1.93 to 0.52)
9 0.99 −0.83 (−2.15 to 0.49)
5 1.00 −0.53 (−1.62 to 0.57)
12 1.00 −0.92 (−2.57 to 0.73)
2 1.00 −0.61 (−1.82 to 0.60)
7 1.00 −0.49 (−1.63 to 0.66)
20 1.00 −0.67 (−3.39 to 2.05)
1 1.00 0.20 (−1.09 to 1.50)
3 1.00 −0.43 (−1.53 to 0.67)
15 1.00 0.42 (−0.92 to 1.77)
17 1.00 1.00 (−0.96 to 2.95)
8 1.00 −0.24 (−1.30 to 0.82)
16 1.00 −0.32 (−1.53 to 0.88)
18 1.00 −0.34 (−1.60 to 0.92)
4 1.00 0.14 (−0.96 to 1.24)
19 1.00 0.20 (−1.01 to 1.42)
10 1.00 0.31 (−1.18 to 1.81)
14 1.00 −0.27 (−1.69 to 1.14)
13 1.00 0.48 (−1.35 to 2.31)
−4 −3 −2 −1 0 1 2 3 4
Lower risk Higher risk
Leiden latent (stratified on Stage and Grade)
Cluster p_adj HR (95% CI)
4 1.00 0.24 (−1.51 to 1.98)
0 1.00 0.10 (−1.42 to 1.62)
14 1.00 0.76 (−1.34 to 2.86)
19 1.00 −1.03 (−3.03 to 0.97)
17 1.00 0.50 (−1.34 to 2.33)
3 1.00 −0.04 (−1.32 to 1.25)
10 1.00 0.77 (−1.10 to 2.63)
16 1.00 0.17 (−1.33 to 1.66)
7 0.68 −3.12 (−6.28 to 0.03)
1 0.97 −2.78 (−6.69 to 1.13)
5 * 0.97 −2.85 (−6.90 to 1.21)
12 1.00 0.27 (−1.07 to 1.60)
2 1.00 −0.25 (−1.46 to 0.97)
6 1.00 −0.28 (−1.97 to 1.41)
8 0.94 −1.24 (−2.84 to 0.37)
20 1.00 −1.28 (−4.40 to 1.85)
15 * 1.00 −1.32 (−6.02 to 3.38)
18 1.00 −0.22 (−3.11 to 2.66)
9 1.00 −0.38 (−2.57 to 1.81)
13 1.00 −0.20 (−2.46 to 2.05)
11 1.00 1.20 (−1.73 to 4.12)
−4 −3 −2 −1 0 1 2 3 4
Lower risk Higher risk
Leiden original (stratified on Stage and Grade)
Cluster p_adj HR (95% CI)
3 1.00 0.65 (−1.09 to 2.39)
2 1.00 1.11 (−0.87 to 3.09)
1 1.00 1.01 (−0.42 to 2.45)
0 1.00 −1.58 (−3.57 to 0.40)
16 * 0.94 8.58 (−0.01 to 17.17)
10 * 1.00 −2.38 (−9.21 to 4.44)
31 * 1.00 −5.52 (−28.72 to 17.67)
9 1.00 0.18 (−3.16 to 3.53)
8 0.98 1.90 (−0.19 to 4.00)
4 * 1.00 −1.41 (−6.22 to 3.40)
45 1.00 0.95 (−1.47 to 3.36)
7 * 1.00 −62.78 (−211.57 to 86.00)
24 * 1.00 −11.76 (−244.43 to 220.92)
23 * 1.00 −59.54 (−1133.39 to 1014.32)
35 * 1.00 −80.84 (−752.21 to 590.52)
33 * 1.00 −7.72 (−46.56 to 31.13)
5 1.00 −0.73 (−3.76 to 2.29)
29 1.00 2.62 (−0.50 to 5.73)
37 * 1.00 −7.26 (−19.51 to 4.98)
41 * 1.00 −1.62 (−16.95 to 13.71)
14 * 1.00 −75.68 (−254.14 to 102.78)
20 1.00 0.73 (−2.04 to 3.50)
6 1.00 −0.09 (−3.40 to 3.23)
22 * 1.00 −14.23 (−127.92 to 99.46)
32 * 1.00 −99.74 (−1609.53 to 1410.04)
17 * 0.57 5.27 (1.01 to 9.54)
18 1.00 0.28 (−1.40 to 1.95)
48 * 1.00 −122.20 (−1258.45 to 1014.04)
19 * 1.00 0.56 (−3.79 to 4.91)
12 * 1.00 −143.52 (−417.53 to 130.48)
15 * 1.00 −73.42 (−457.70 to 310.87)
13 * 1.00 −50.93 (−130.55 to 28.70)
11 1.00 1.59 (−1.26 to 4.44)
34 * 1.00 −5.01 (−39.58 to 29.57)
30 1.00 1.67 (−0.48 to 3.82)
36 * 1.00 −4.21 (−20.45 to 12.03)
53 * 1.00 −75.48 (−5489.36 to 5338.40)
51 * 1.00 9.66 (−40.44 to 59.77)
25 * 1.00 −122.05 (−1248.05 to 1003.96)
50 * 1.00 83.47 (−3325.77 to 3492.71)
42 * 1.00 −96.54 (−1922.64 to 1729.55)
21 1.00 −0.19 (−3.01 to 2.63)
46 * 1.00 −64.63 (−751.12 to 621.86)
39 * 1.00 −36.46 (−1660.80 to 1587.88)
27 * 1.00 −81.92 (−735.26 to 571.43)
28 * 1.00 −6.83 (−90.65 to 77.00)
44 * 1.00 −14.14 (−5667.16 to 5638.87)
52 * 1.00 −122.76 (−1489.01 to 1243.49)
49 * 1.00 −1134.26 (−552077.99 to 549809.47)
38 * 1.00 62.99 (−3180.45 to 3306.44)
54 * 1.00 0.00 (−8214015974.45 to 8214015974.45)
40 * 1.00 −6.75 (−94.69 to 81.18)
43 * 1.00 −4.47 (−94.38 to 85.43)
47 * 1.00 0.00 (−8214015974.45 to 8214015974.45)
−4 −3 −2 −1 0 1 2 3 4
Lower risk Higher risk

Supplementary Figure 6. CoxPH model hazard ratios with cluster prevalence per mm^2 of tissue for clusters from FlowSOM and Leiden using original and latent spaces for Ali-BC and Jackson-BC. P-values are multiple tests corrected using Benjamini-Hochberg correction. *: Circle size capped due to high standard error (se.coef > 2).

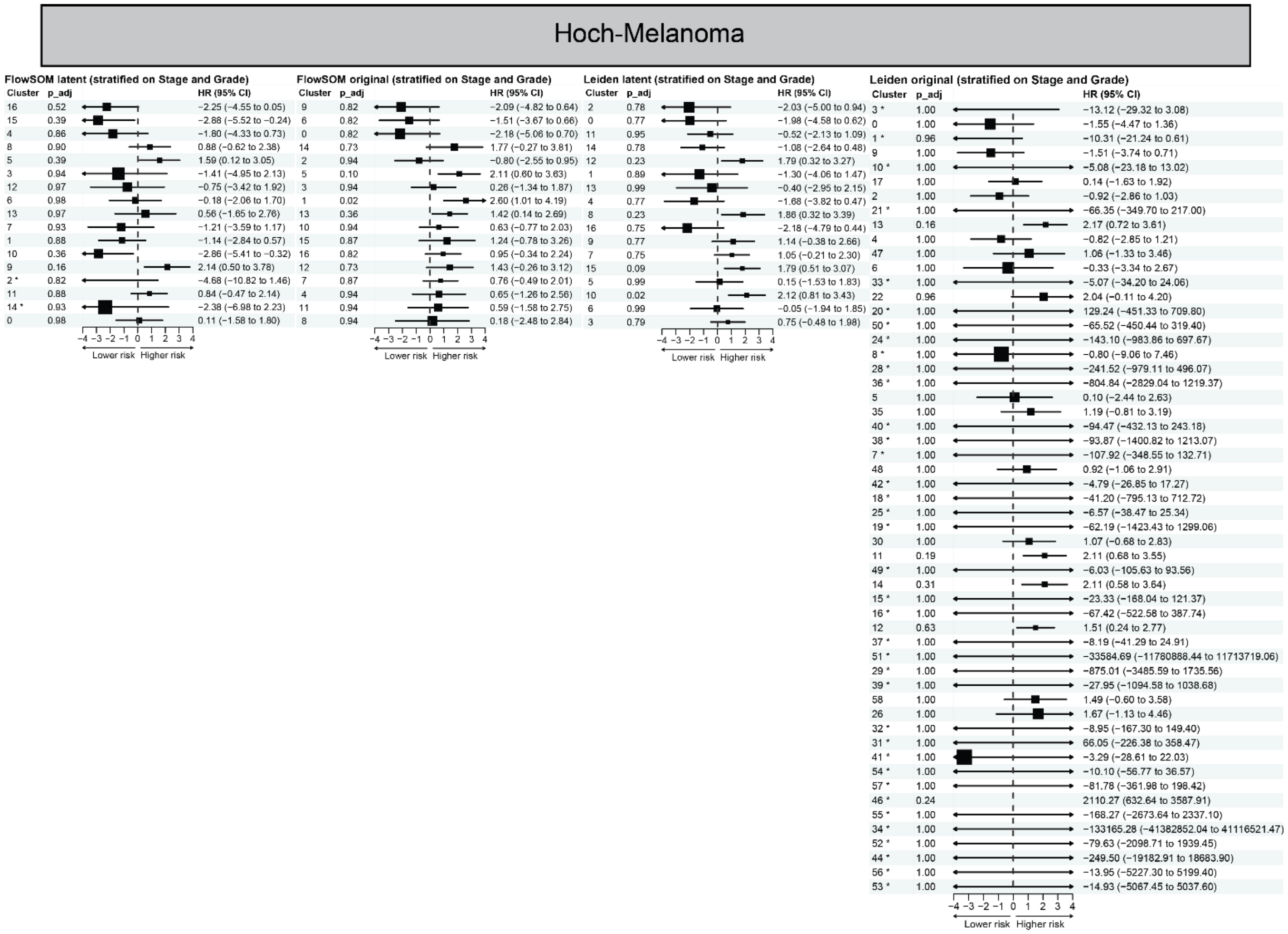

Supplementary Figure 7. CoxPH model hazard ratios with cluster prevalence per mm^2 of tissue for clusters from FlowSOM and Leiden using original and latent spaces for Hoch-Melanoma dataset. P-values are multiple tests corrected using Benjamini-Hochberg correction. *: Circle size capped due to high standard error (se.coef > 2).

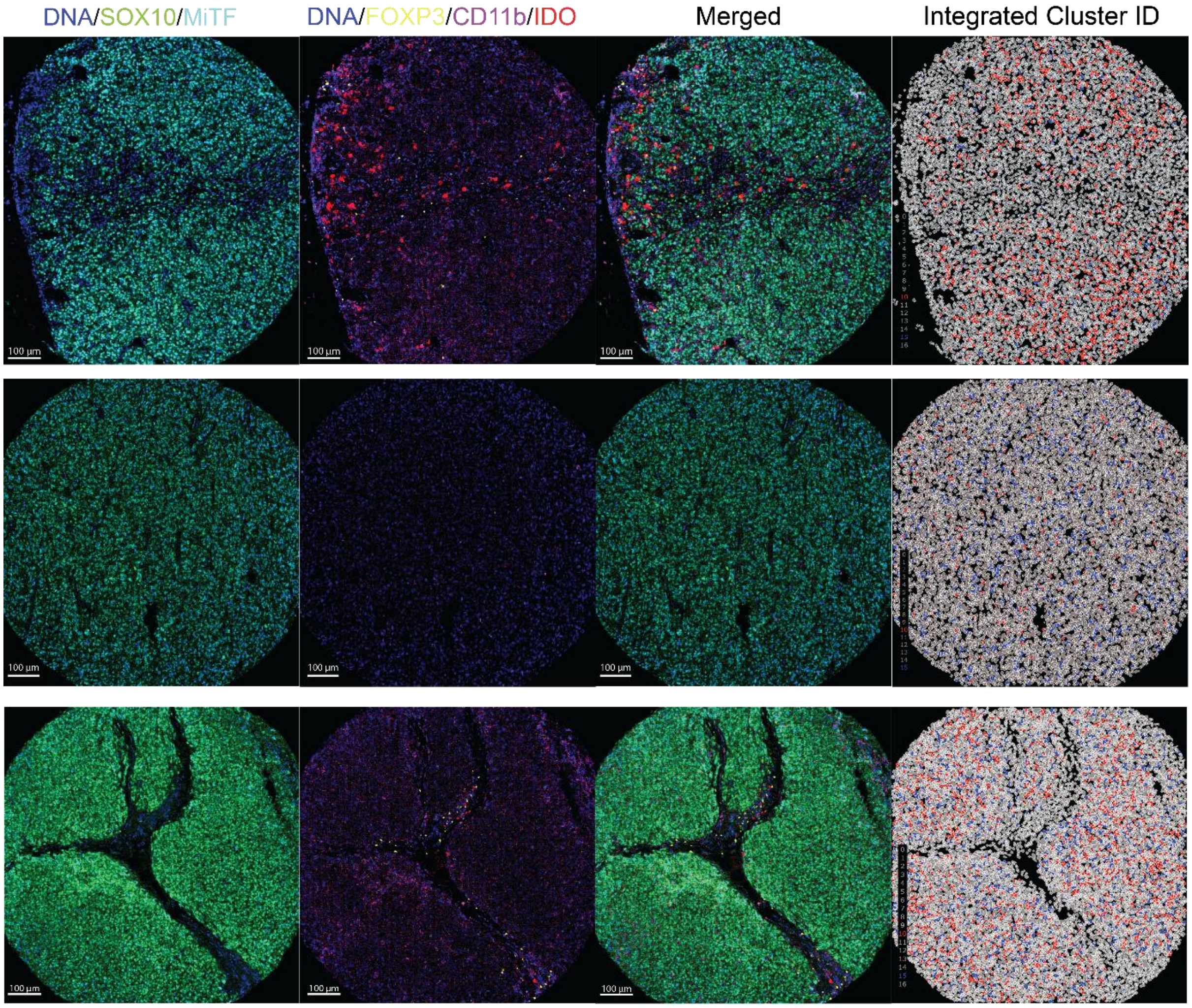

Supplementary Figure 8. IMC images from Hoch-Melanoma dataset showing patients that had a high number of cells belonging to cluster 10 (top), cluster 15 (middle) or both (bottom). SOX10 and MiTF are tumour cell markers, FOXP3, CD11b and IDO are markers for Tregs, macrophages and T cell suppressive microenvironment, respectively. Integrated cluster IDs, cluster 10 in red and cluster 15 in blue.

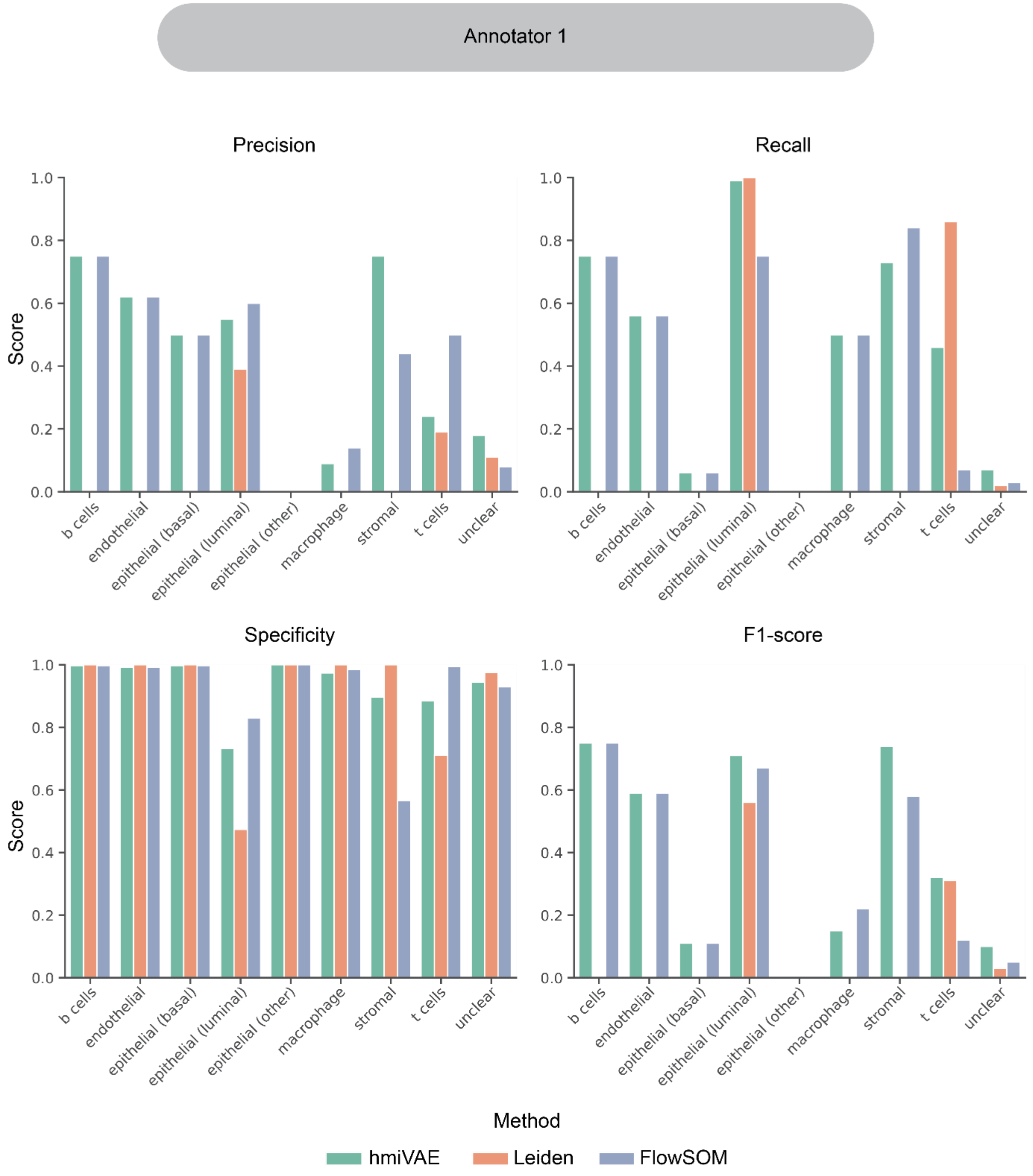

Supplementary Figure 9. Precision, Recall, Specificity and F1-Score for all methods in calling the different cell populations when compared to manually annotated ground truth labels of 500 cells annotator 1.

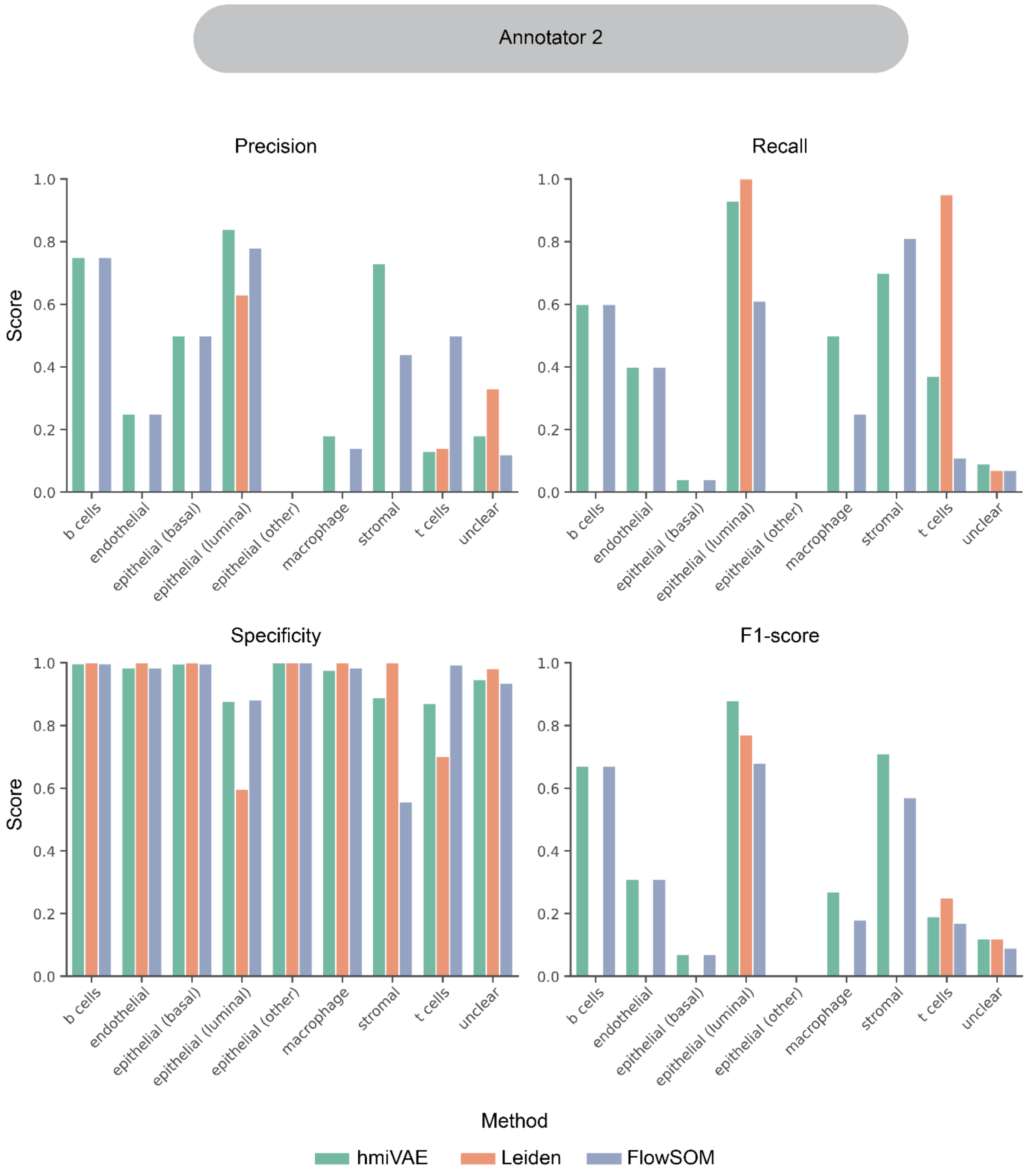

Supplementary Figure 10. Precision, Recall, Specificity and F1-Score for all methods in calling the different cell populations when compared to manually annotated ground truth labels of 500

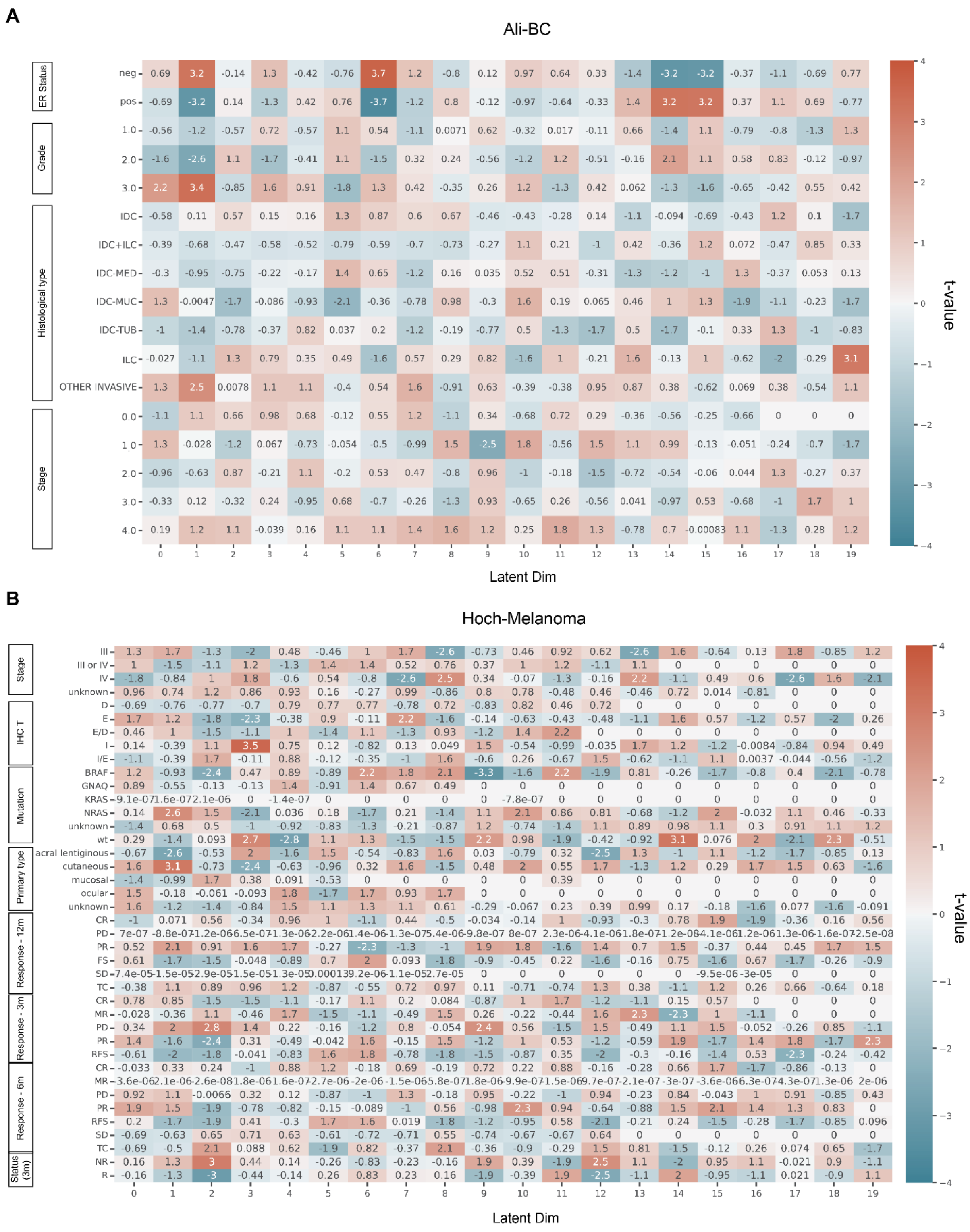

Supplementary Figure 11. Clinical association of hmiVAE latent space dimensions for Ali-BC (**A**) and Hoch-Melanoma (**B**).

A

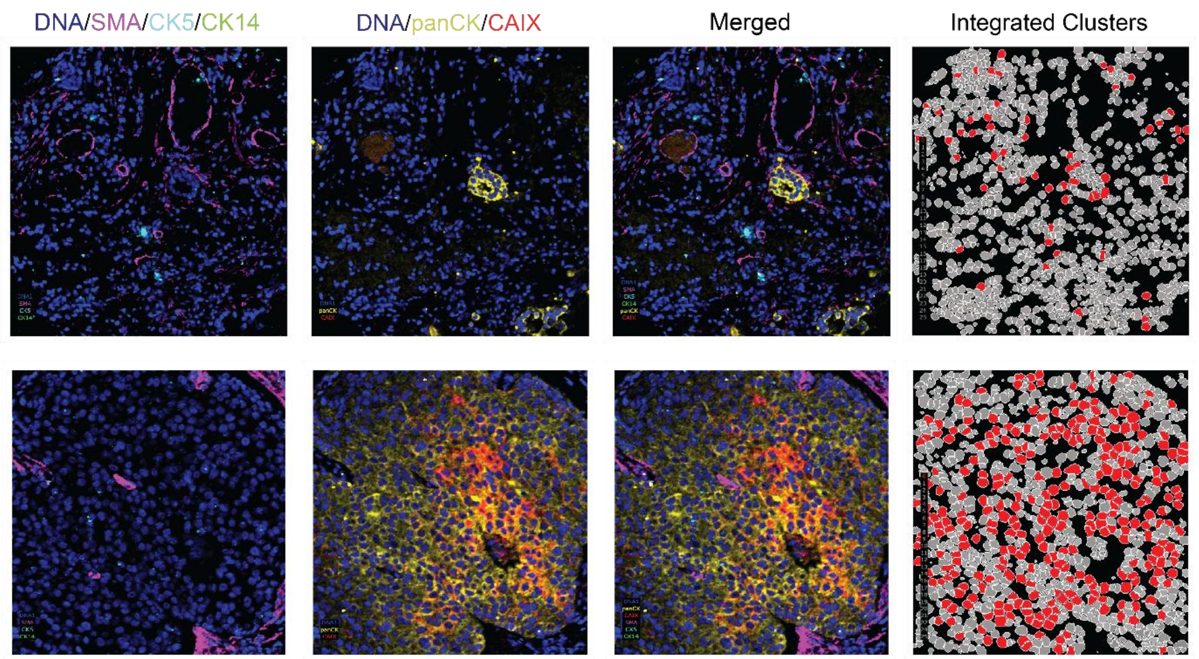

B

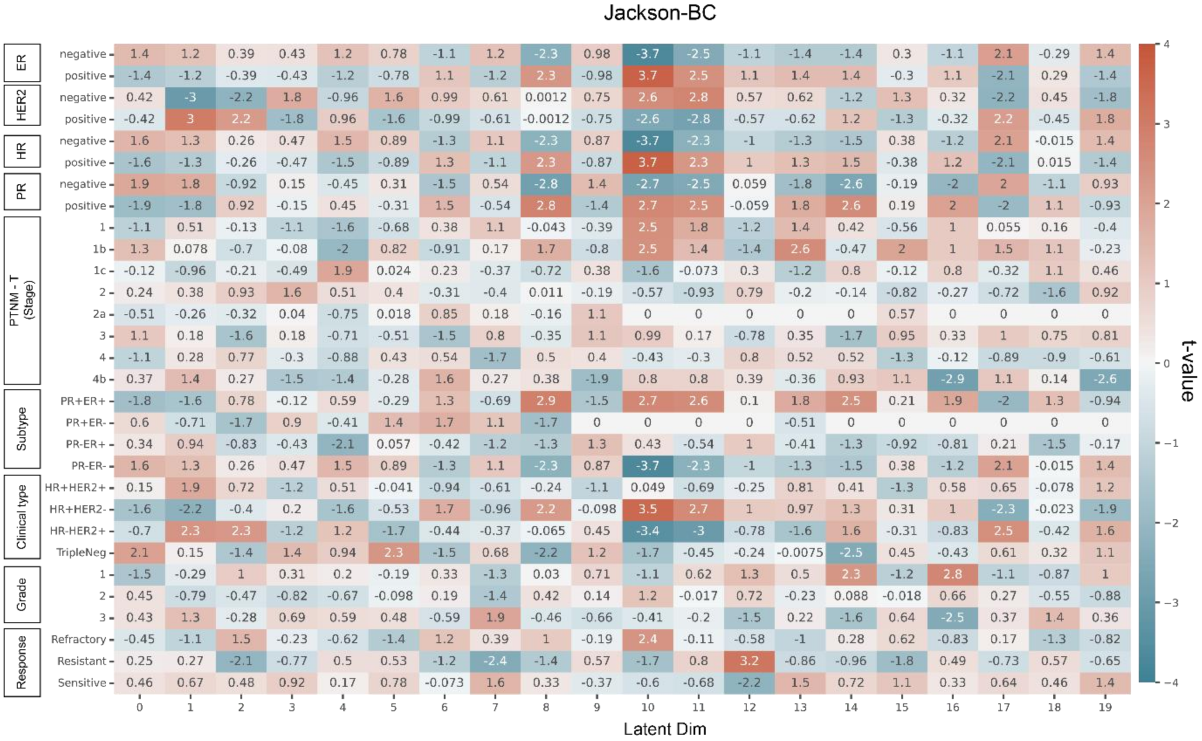

Supplementary Figure 12. A IMC images from the Ali-BC dataset showing marker expression patterns for SMA, CK5, CK14, panCK and CAIX in a patient with low number of cells belonging to cluster 0 (top) and a patient with a high number of cells belonging to cluster 0 (bottom). Cells

belonging to integrated cluster 0 are shown in red, while all other clusters are shown in shades of grey. **B** Clinical association of hmiVAE latent space dimensions for the Jackson-BC dataset.

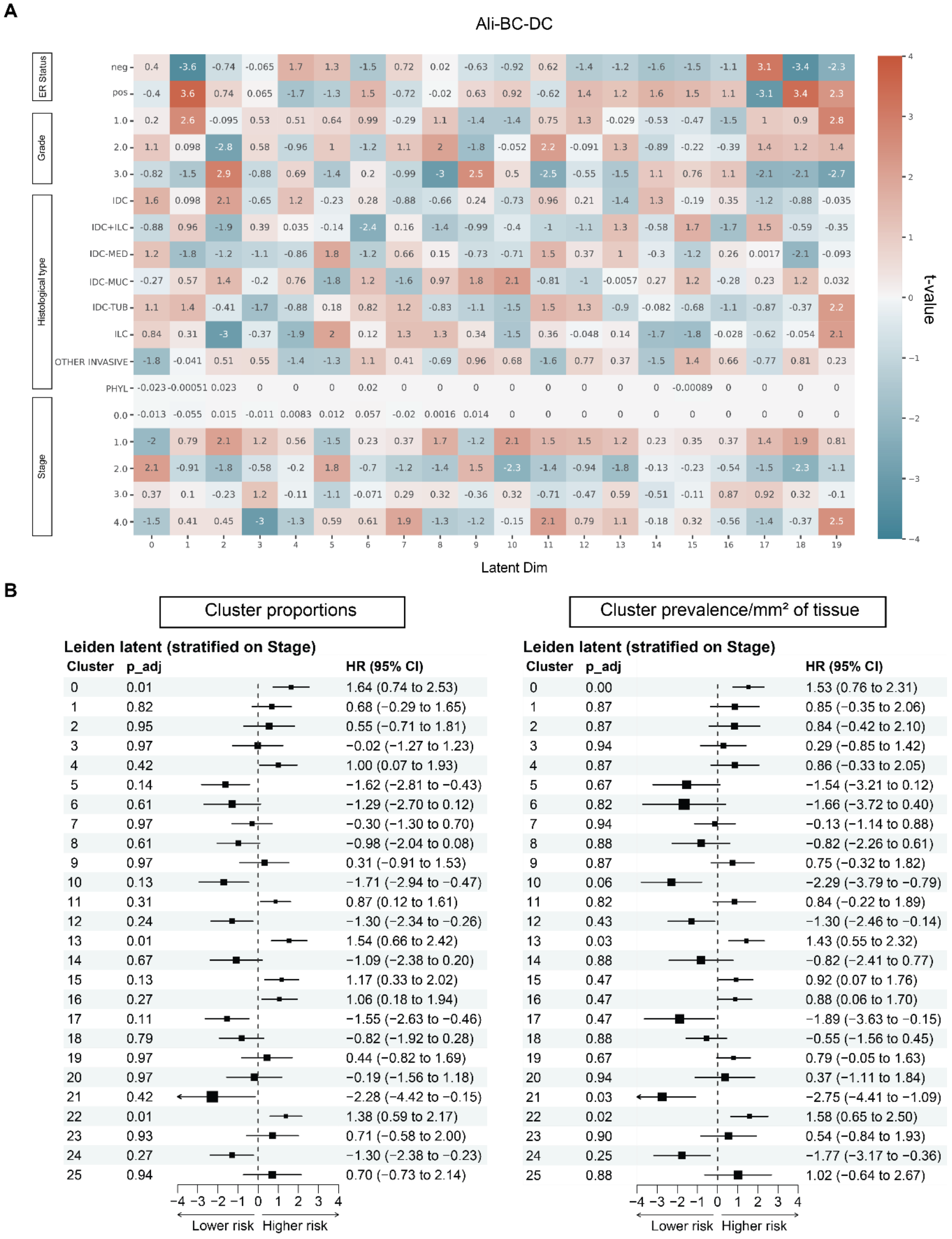

Supplementary Figure 13. A Clinical association of hmiVAE latent space dimensions for the Ali-BC-DC dataset. **B** CoxPH model hazard ratios with cluster proportion and cluster prevalence per mm^2 of tissue for clusters from the latent features from Ali-BC stratified on stage. P-values are multiple tests corrected using Benjamini-Hochberg correction.

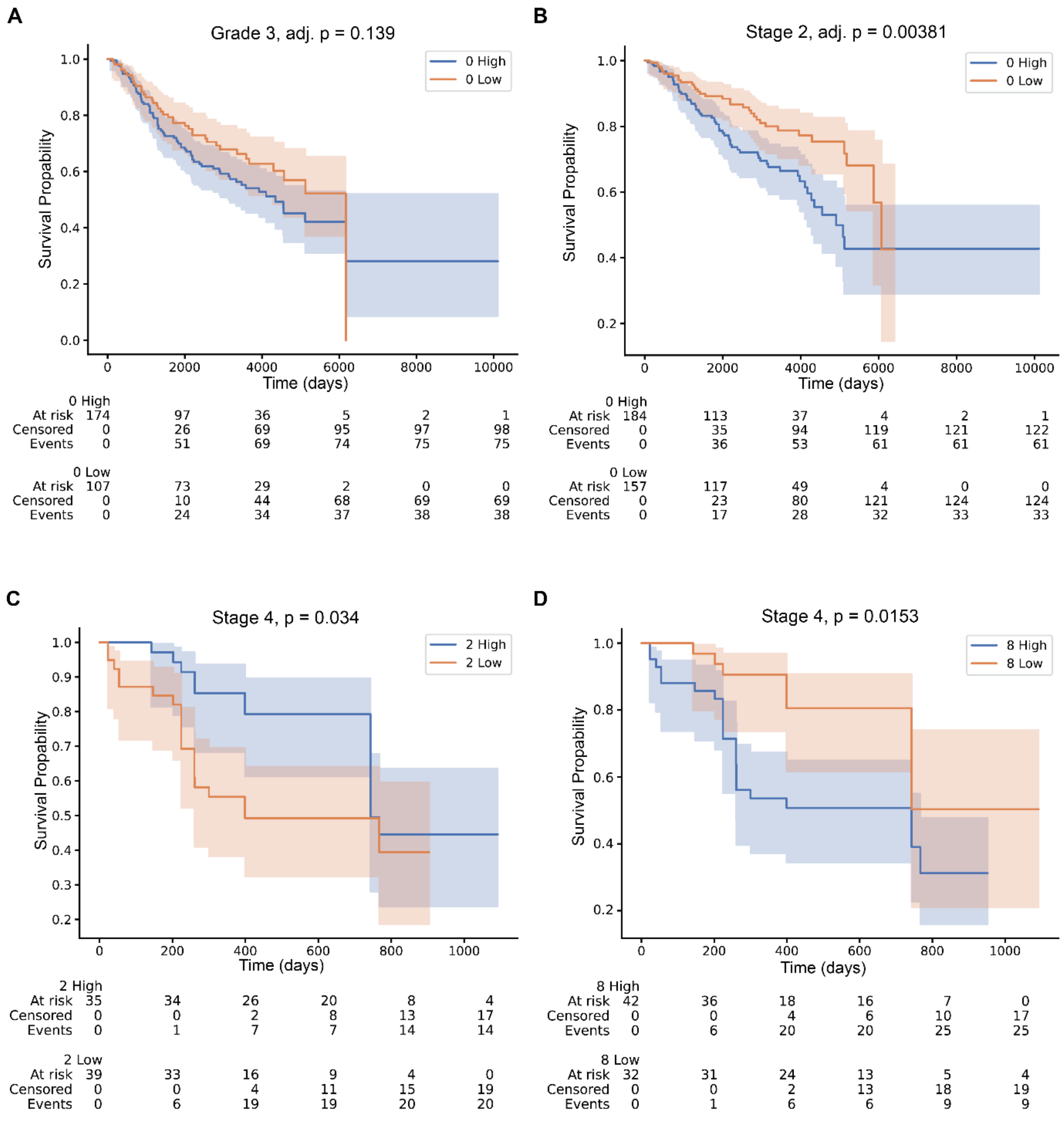

Supplementary Figure 14. Kaplan-Meier curves for integrated cluster 0 from Ali-BC, patients with cancer grade 3 (**A**) and stage 2 (**B**) and Kaplan-Meier curves for patients from Hoch-

Melanoma with cancer stage 4 for integrated cluster 2 (**C**) and 8 (**D**). P-values are from a log-rank test.

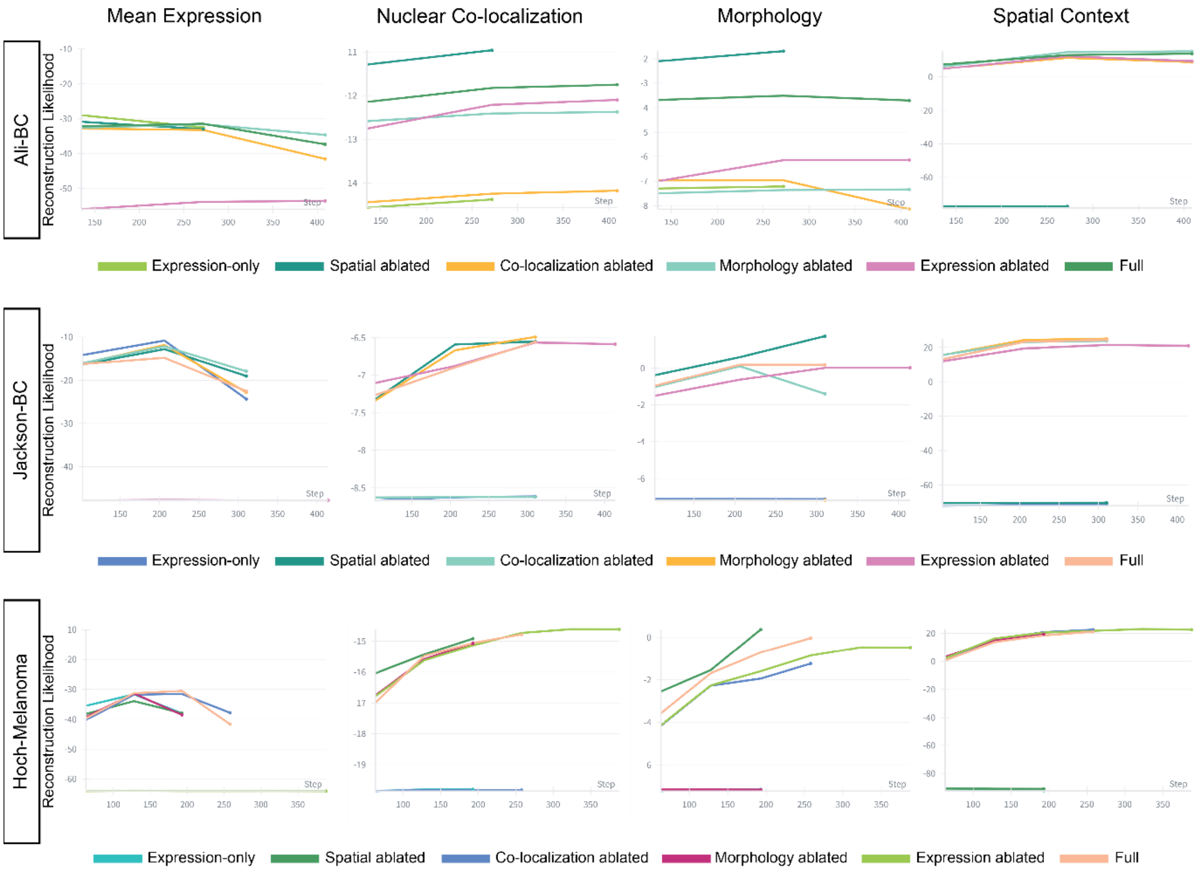

Supplementary Figure 15. Reconstruction likelihood curves for all views for all ablation runs across all three datasets.

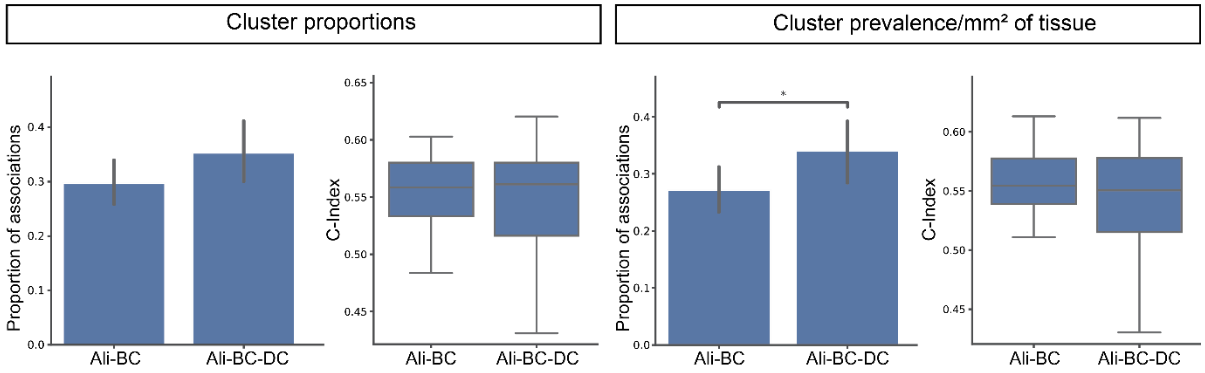

Supplementary Figure 16. Comparing clinical and survival association between two segmentations of the Ali-BC dataset. T-test for statistical comparison and p-values are multiple

tests corrected using Benjamini-Hochberg correction. Error bars indicate a 95% confidence interval. P-value annotation: *: 0.01 < p <= 0.05; **: 0.001 < p <= 0.01; ***: 0.0001 < p <= 0.001; ****: p <= 0.0001. All n.s. comparisons are omitted.

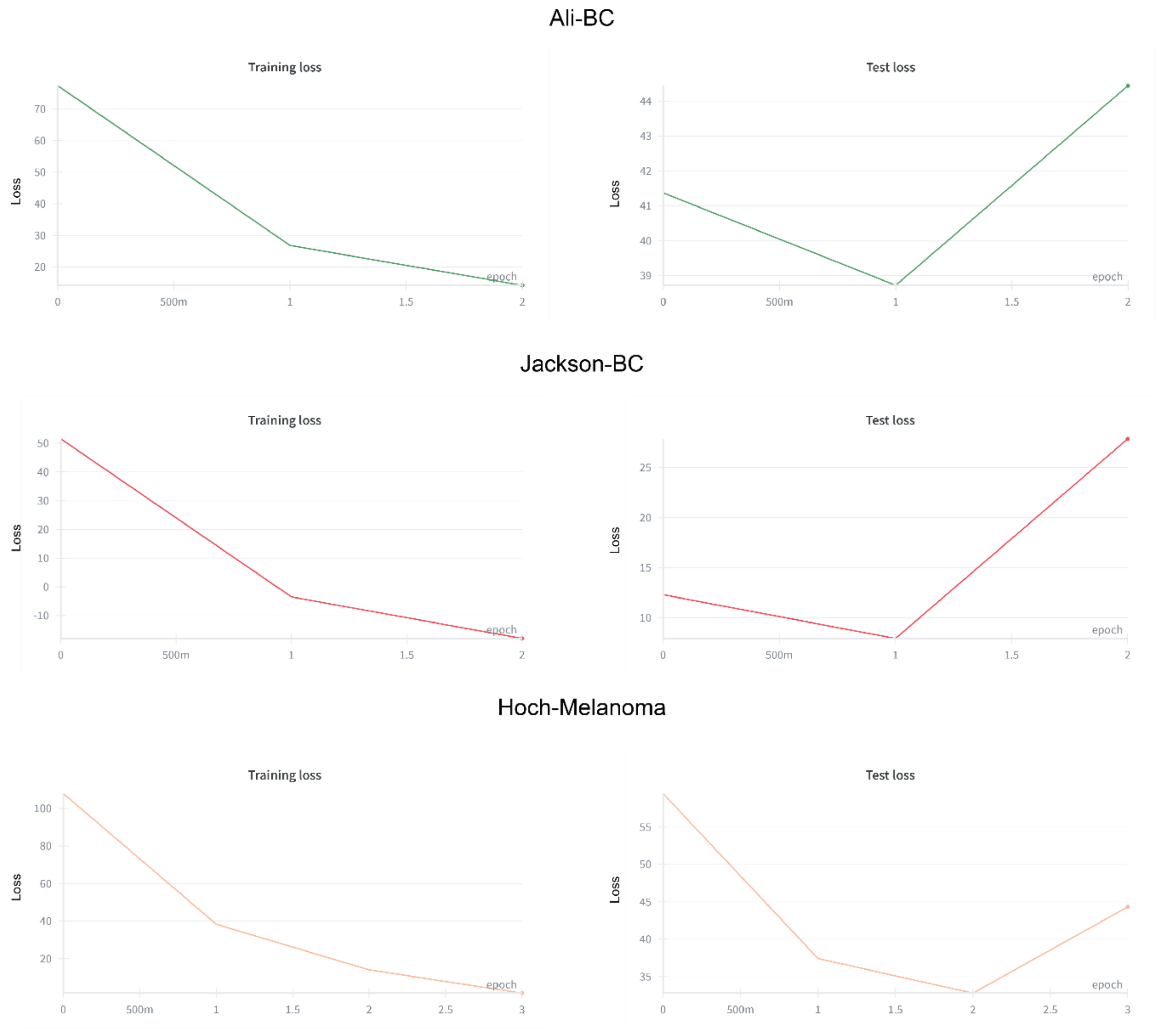

Supplementary Figure 17. Train and test loss curves using the best hyperparameter combination for all datasets.

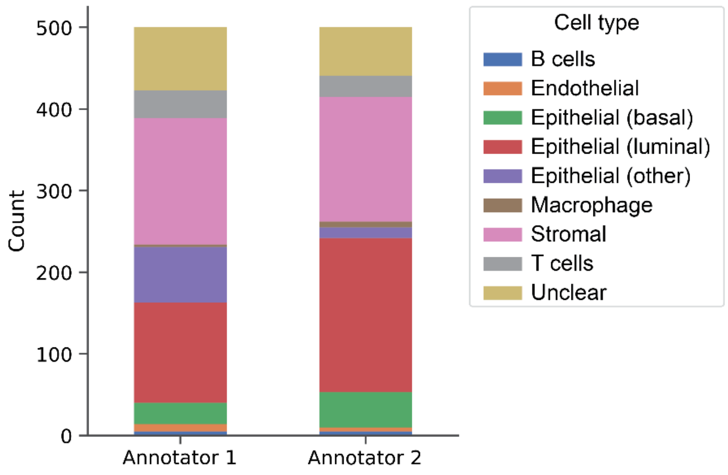

Supplementary Figure 18. Cell type distributions in the manually-annotated ground truth dataset.

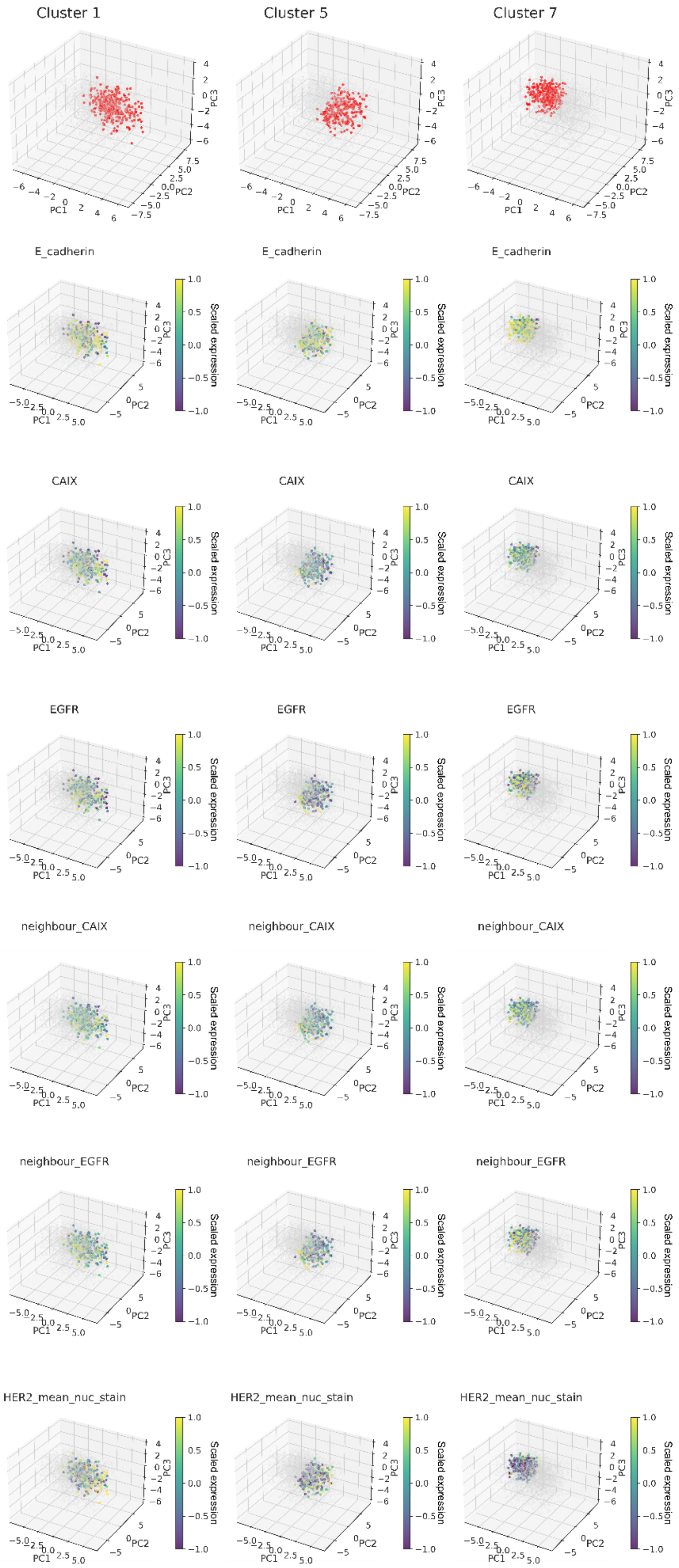
Cluster 1
Cluster 5
Cluster 7
E_cadherin
CAIX
EGFR
neighbour_CAIX
neighbour_EGFR
HER2_mean_nuc_stain
PC1
PC2
PC3
Scaled expression

Supplementary Figure 19. 3-dimensional PCA plot showing marker expressions for integrated clusters 1, 5 and 7 from the Jackson-BC dataset.

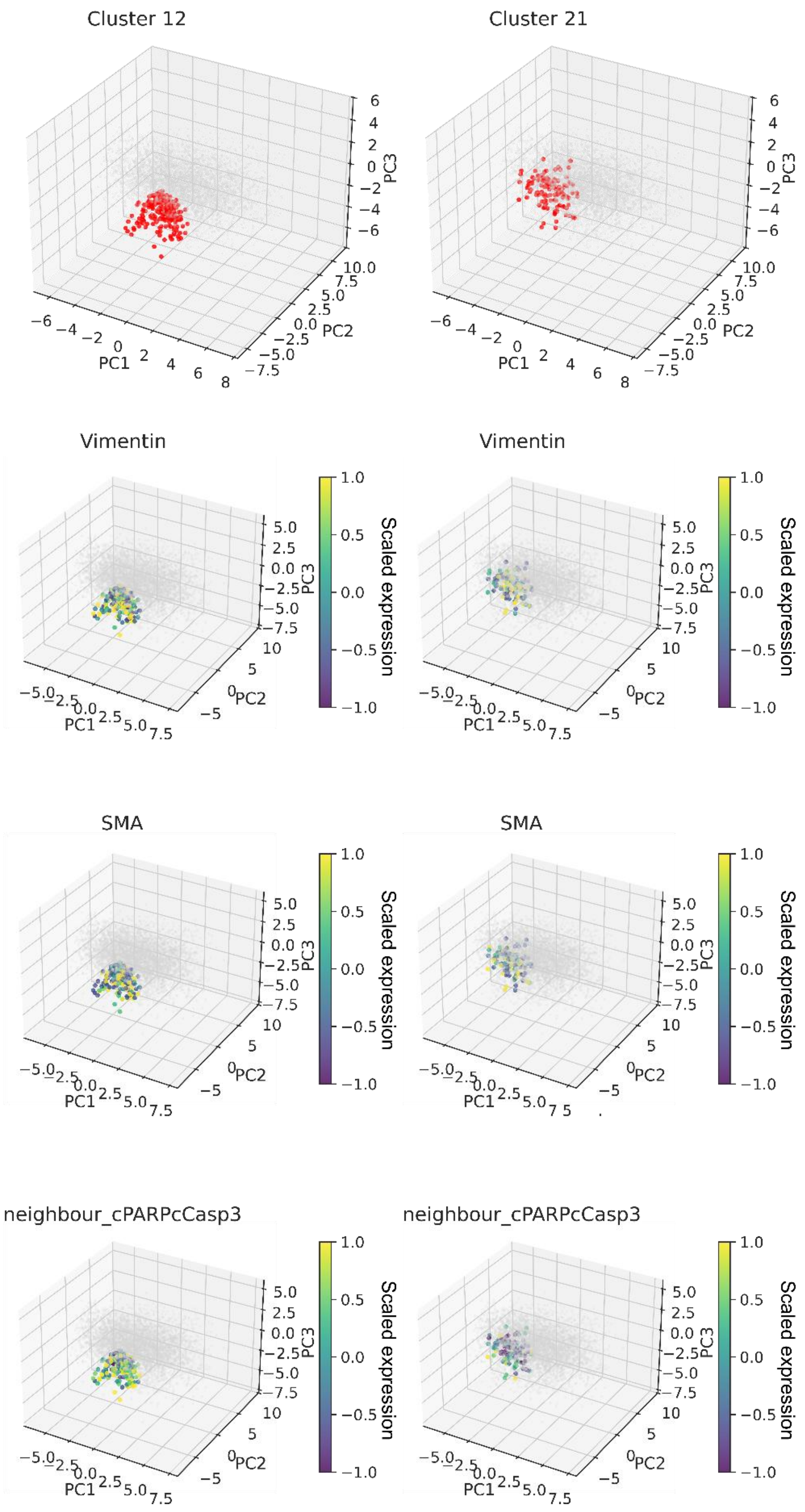

Supplementary Figure 20. 3-dimensional PCA plot showing marker expressions for integrated clusters 12 and 21 from the Ali-BC dataset.

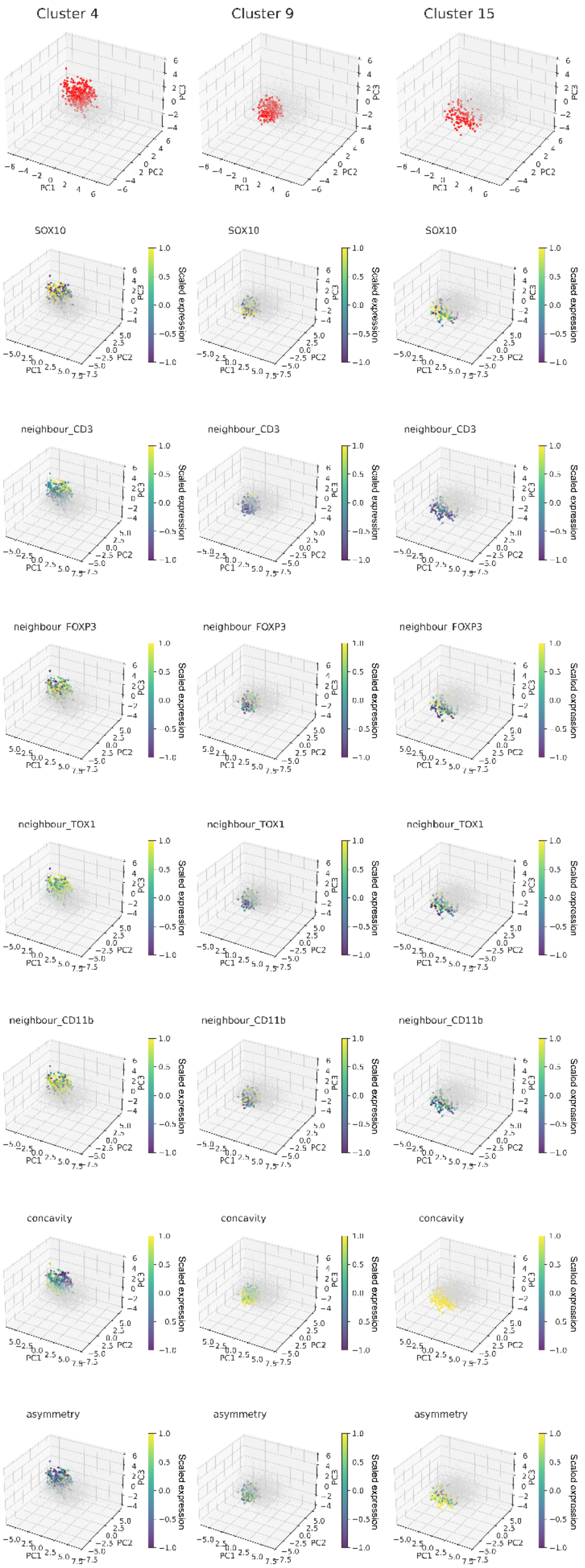
Cluster 4
Cluster 9
Cluster 15
SOX10
neighbour_CD3
neighbour_FOXP3
neighbour_TOX1
neighbour_CD11b
concavity
asymmetry
Scaled expression
PC1
PC2
PC3

Supplementary Figure 21. 3-dimensional PCA plot showing marker expressions for integrated clusters 4, 9 and 15 from the Hoch-Melanoma dataset.

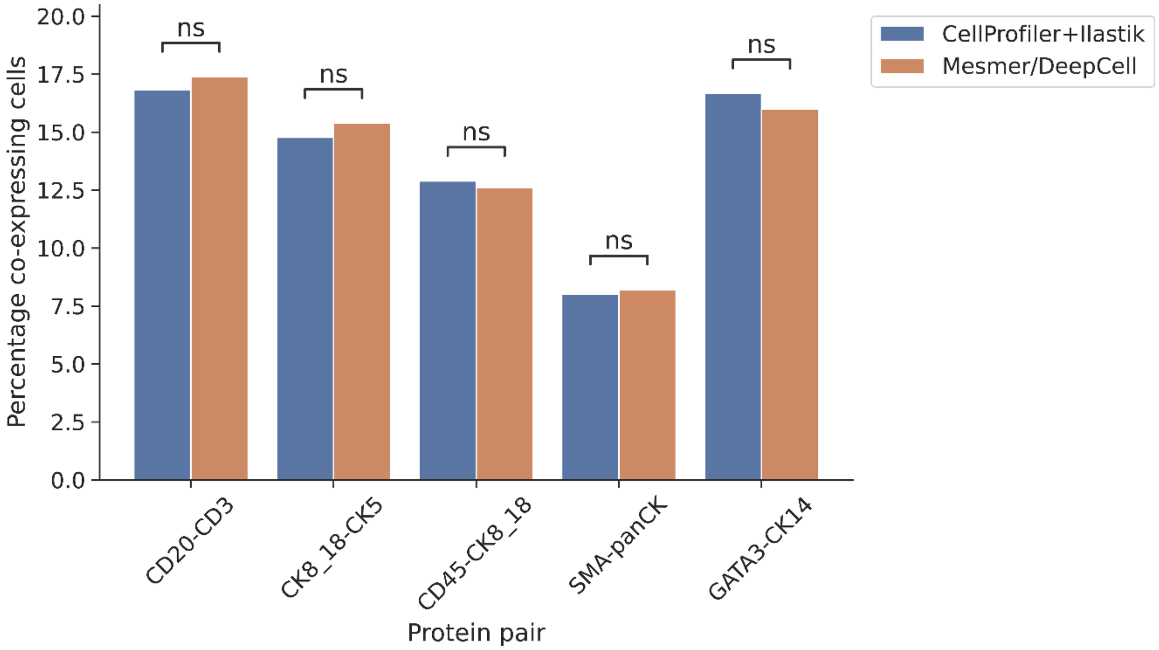

Supplementary Figure 22. Segmentation quality check by comparing number of cells co-expressing proteins known to not be co-expressed for segmentation methods CellProfiler+Ilastik and Mesmer/DeepCell.
